# Redesign of TALE proteins for DNA-templated assembly of protein fibers

**DOI:** 10.1038/s41467-026-73313-8

**Published:** 2026-05-19

**Authors:** Robbert J. de Haas, Mark D. Langowski, Andrew J. Borst, Visakh V. S. Pillai, Gwendolyn E. Hoffmann, Martin Bongers, Matthias Mulder, Suna Cheng, Catherine Treichel, Elizabeth M. Leaf, Mengyu Wu, Eric M. Lynch, Justin M. Kollman, Francesco S. Ruggeri, Carl Walkey, Renko de Vries, Neil P. King

**Affiliations:** 1 Department of Physical Chemistry and Soft Matter, Wageningen University & Research, Wageningen, The Netherlands; 2 Department of Biochemistry, University of Washington, Seattle, WA USA; 3 Institute for Protein Design, University of Washington, Seattle, WA USA; 4 Department of Organic Chemistry, Wageningen University & Research, Wageningen, The Netherlands

**Keywords:** DNA-binding proteins, Bioinspired materials, Bioinspired materials, Atomic force microscopy, Cryoelectron microscopy

## Abstract

Many viral proteins self-assemble into capsid structures, often using their genetic material as a template for assembly. To date, de novo designed capsid-like proteins do not require genetic material as a template for assembly, which can be both an advantage and a disadvantage depending on the use case. Templates are indispensable, for example, in the assembly of linear structures with well-defined lengths. As a first step towards fully de novo designed templated assembly, here we redesign proteins from the Transcription activator-like effector (TALE) family of transcriptional regulators to polymerize on double-stranded DNA (dsDNA) templates. Starting from natural TALE protein sequences, we create idealized repeat proteins with sequence-independent DNA binding properties that self-assemble to form linear protein-DNA complexes with template-controlled lengths. We use high-resolution atomic force microscopy (AFM) and cryo electron microscopy (cryo-EM) to characterize the three-dimensional structures of the DNA-protein hybrid complexes. In these structures, a protein filament helically wraps around the dsDNA similar to natural TALE proteins. As an example application of these materials, we show the system can be used for repetitive peptide antigen display at precisely controlled repeat distances, and that such immunogens elicit robust antigen-specific antibodies in mice.

## Abstract (web summary)

Viral capsids use their genome as an assembly template, but designed proteins lack this feature. Here, the authors redesign TALE proteins to polymerize on DNA, forming linear fibers that display peptide antigens and elicit antibodies in mice.

## Introduction

Viruses self-assemble into highly ordered nanoscale architectures with high fidelity, despite the presence of a complex background of host biomacromolecules and widely varying assembly conditions. One particularly effective strategy to ensure efficient encapsulation under these challenging and varying conditions is to use the virus genome as a blueprint to guide the assembly process^1^. For example, the Tobacco Mosaic Virus capsid proteins nucleate via a packaging signal in its RNA genome, which initiates a highly ordered RNA-templated assembly process^2,3^.

To date, the strategy of cargo-templated assembly has not yet been fully exploited in designing artificial capsid systems composed of de novo proteins^4^. While there are many use cases for artificial capsids that are not cargo-templated, one can also imagine many use cases where cargo-templated assembly would be beneficial. For example, protein-encapsulated dsDNA could be useful as a vaccine, with the protein serving as a multivalent antigen display scaffold^5^ and the cargo potentially acting as an adjuvant through activation of Toll-like receptors (TLRs)^6^. Another use case could be nucleic acid delivery^7,8^. Finally, from a nanomaterials design perspective, templated assembly is one of the few established mechanisms for achieving assembly into linear structures of well-defined lengths^9–12^.

Minimal DNA-binding coat proteins have been designed previously^10^, but these were based on simple polypeptide domains that are less engineerable. A number of different naturally occurring protein families have evolved to bind and, in some cases, assemble on dsDNA^12,13^, and some of these are attractive building blocks for testing novel design strategies. For example, the TALE proteins are ideal scaffolds for two reasons. First, they have a quasi-repetitive core consisting of well-defined repeat units that could serve as the basis for idealized repeat proteins. Equally important, the interfaces between the repeated motifs within each subunit could be repurposed to act as an interface that drives the self-assembly of multiple subunits to form a polymer. Second, TALE proteins are known to undergo a slight allosteric transition upon DNA-binding^14,15^, a mechanism that could be exploited to favor assembly on the DNA template and to prevent assembly in the absence of DNA templates.

Here, we leveraged these features of TALE proteins to computationally design idealized repeat proteins with sequence-independent DNA binding properties. We found that the proteins self-assembled on dsDNA templates to form highly ordered linear polymers that can be used to display peptide motifs with precisely controlled spacing.

## Results

### Design of sequence-independent DNA-templated protein polymers

**Fig. 1:**
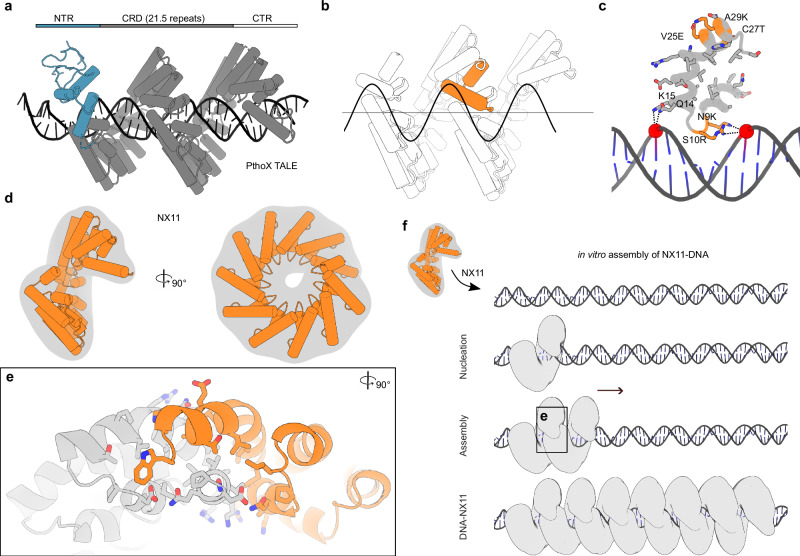
Design of sequence-independent DNA-templated protein polymers. **a** PthoX transcription activator-like effector protein (TALE). Top: domain architecture. NTR N-terminal region, CRD central repeat domain containing helix–turn–helix repeats where each CRD repeat binds to a specific DNA base pair, CTR C-terminal region. Bottom: Cylinder cartoon diagram of the crystal structure of PthoX (PDB ID: 3UGM17). Only a part of the NTR (blue) and the full CRD (gray) were crystallized. **b** A single CRD repeat is extracted, aligned to an idealized dsDNA strand, and helically symmetrized following the helical pitch of DNA of 34 Å and 10.5 monomers per turn. **c** Designed monomer, NX1, with symmetrical mutations indicated. S9R and N10K remove specificity and introduce additional non-specific interactions through ionic interactions with the phosphate backbone (red spheres) of DNA. Cys27Thr removes potential dimerization while Val25Glu and Ala29Lys mutate hydrophobic surface residues to a stabilizing salt-bridge. **d** Structure of NX11, propagated from NX1 repeats. **e** NX–NX intra-repeat interface formed by two adjacent NX proteins. This is the same interface as the inter-repeat interface and is mostly hydrophobic. **f** Hypothesized in vitro assembly of NX11 with DNA to form NX11–DNA complexes. Upon mixing, random nucleation occurs due to non-specific interactions between NX11 and DNA, followed by assembly starting from the nucleation site due to additional NX11–NX11 protein interfaces that form with multiple adjacent NX11 proteins (see (**e**)). Assembly continues until full coverage of the DNA is achieved, resulting in NX11–DNA complexes.

We began from the known sequences and structures of sequence-specific TALE DNA-binding proteins. TALEs comprise three simple structural domains: an N-terminal region (NTR), a central repeat domain (CRD), and a C-terminal region (CTR) (Fig. 1a). The NTR has been suggested to serve as the indispensable nucleation site for DNA binding^16^, while the CRD consists of a set of quasi-repetitive 33–35-residue motifs^17^. The CRD repeats follow the major groove of dsDNA and form sequence-specific interactions solely through two adjacent residues (called the repeat-variable diresidue, RVD) located in the turn of the helix hairpin. Diverse combinations of RVD confer a selective preference for binding to nucleotides A, C, T, or G^18^. Owing to their modular, repetitive structures and simple rules for DNA-binding specificity, we hypothesized that CRD repeats would make an excellent starting point for the design of repetitive sequence-independent DNA-binding proteins. We selected TALE PthoX1 as a starting point for design, since a high-resolution crystal structure was available (Fig. 1a; PDB ID: 3UGM)^17^.

We extracted the 7th CRD 33-residue repeat (residues 498–527) because it already had a deletion of one of the RVD sites. We symmetrized it following the 35 Å superhelical pitch and 10.5 monomers per turn (one repeat per base pair) of idealized B-form DNA (Fig. 1b). We then used Rosetta to symmetrically redesign the monomer (see “Methods”). Residues 9 and 10 on the helix preceding the RVD site were redesigned with constrained backbone relaxation to facilitate minor backbone movements, while the native non-specific DNA-interacting residues Gln14 and Lys15 (ref. ^19^ were restricted from repacking. Rosetta suggested Ser9Lys and Asn10Arg mutations could contribute to non-specific DNA binding via ionic interactions with phosphate groups in the DNA backbone (Fig. 1c). We also conservatively redesigned hydrophobic residues on the monomer surface to potentially improve its stability and solubility. Cys27Thr was included to remove potentially problematic disulfide bond formation, and Val25Glu and Ala29Lys introduced a stabilizing salt bridge in place of surface hydrophobes (Fig. 1c).

Next, we used Rosetta Remodel^20^ to fuse position 34 of one symmetrically arranged monomer to position 1 of its neighboring monomer (see “Methods”). Repeat proteins with defined numbers of repeats were generated by symmetrically propagating the backbone of the monomer. We refer to the resultant sequence-independent DNA-binding repeat proteins as NucleoX, or NX, followed by a number referring to the number of repeats present (e.g., NX11 has 11 repeats) (Fig. 1d).

The designed NX proteins, like the naturally occurring TALE proteins, have predominantly hydrophobic interfaces between connected CRD repeats (Fig. 1e). Unlike the TALE proteins, however, which have capping CTR and NTR domains, the NX proteins have exposed CRD repeat interfaces at their N and C termini. We hypothesized that the CRD would allow for interactions between neighboring NX proteins that would drive self-assembly and cooperative DNA binding (Fig. 1f). This hypothesis assumed that the non-specific protein-DNA interactions we engineered into the NX proteins would be sufficient to nucleate their assembly on DNA, even in the absence of the NTR domain that has this function in the natural TALE proteins^15^. Furthermore, because TALE proteins are known to undergo a conformational change upon binding to DNA^21,22^, we assumed that the structure of the intermolecular interfaces would be slightly different in solution, which would disfavor assembly prior to DNA binding.

### Assembly with DNA and nuclease protection

**Fig. 2:**
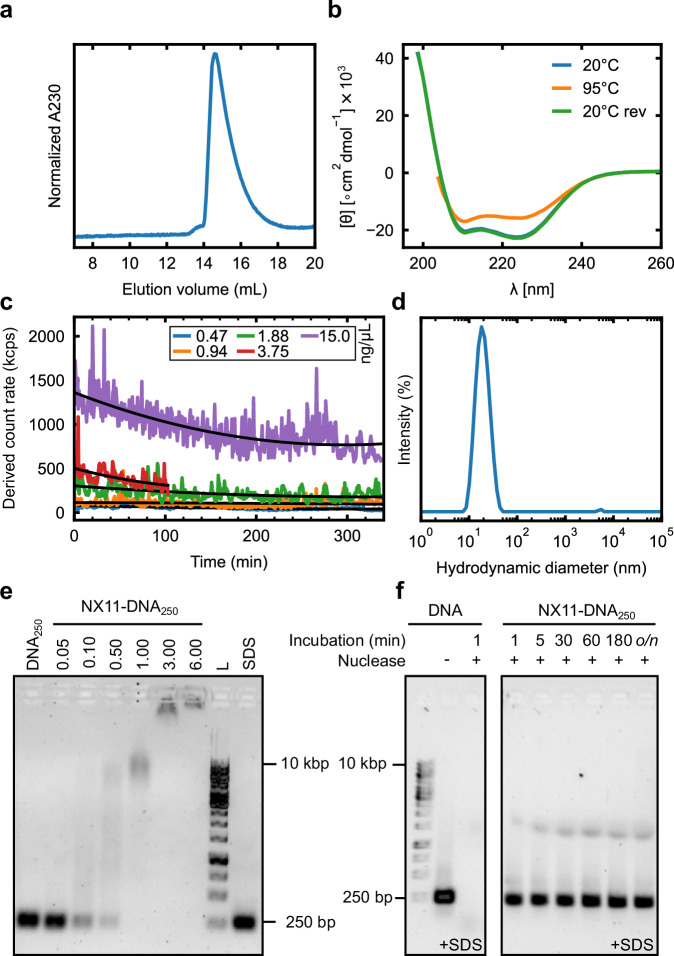
Assembly with DNA and nuclease protection. **a** Size-exclusion chromatography on Superdex 200 10/300 (Cytiva) demonstrating elution of monomeric NX11 protein at ~15 mL, corresponding to a molecular weight of ~42 kDa. **b** Circular dichroism of 0.1 mg/mL purified NX11 in PBS. Molar residue ellipticity [*θ*] is plotted as a function of wavelength, showing a predominantly α-helical profile. Heating to 95 °C led to a minor change in ellipticity, and subsequent cooling back to 20 °C (20 °C rev) gave the same profile as before, demonstrating that NX11 proteins are thermostable. **c** Assembly kinetics of NX11 with different concentrations of 250 bp DNA (DNA250), tracked with static light scattering. NX11-protein was kept at a threefold excess relative to the number of DNA250 binding sites (assuming 1 NX per bp). The derived count rate in kilo counts per second (kcps) was recorded over time as a measure of assembly. Upon mixing NX11 and DNA, the scattering intensity increases rapidly, followed by a gradual decrease. **d** Dynamic light scattering intensity vs. size of assembled NX11–DNA48 complexes in PBS following >4 h incubation at room temperature. The average hydrodynamic diameter was 18.3 nm, and the polydispersity index was 0.12. The corresponding autocorrelation function is shown in Supplementary Fig. 2. **e** Electrophoretic mobility shift assay for detecting assembly of NX11 with DNA250. NX11 was added at different ratios of excess. Due to charge neutralization and an increase in the DNA–protein complex size, the DNA migrates less far into the gel, indicating complex formation. L is a 1 kb Gene ruler (Thermo Fisher Scientific), SDS is NX11–DNA complex loaded in the presence of ~0.2% SDS in the loading dye to denature the complexes. The experiment was independently repeated more than five times with similar results. **f** Nuclease protection assay. The gel was run under denaturing conditions (~0.2% SDS in loading dye) to clearly visualize the DNA. Left: control free DNA was fully degraded in <1 min by Benzonase nuclease. Right: NX11–DNA complexes show full-length DNA250 at all incubation times, indicating robust DNA protection from Benzonase. The experiment was independently repeated twice with similar results.

To facilitate purification, we generated expression plasmids encoding NX5 and NX11 with a cleavable hexahistidine tag at the N terminus. Potential steric hindrance in the protein–protein interface was minimized by eliminating the two terminal loop residues of the final repeat (Thr and Leu). Finally, a sole Trp residue was affixed to enable protein quantification via UV/vis spectroscopy. We expressed the NX5 and NX11 proteins in *E. coli*. Both constructs expressed well, and were purified using immobilized metal affinity chromatography (IMAC) (Supplementary Fig. 1). For simplicity we will focus here on NX11, but further biochemical and assembly characterization of NX5 can be found in the Supplementary Materials (Supplementary Fig. 2). Importantly, NX11 eluted as a monomer from size-exclusion chromatography (SEC) (Fig. 2a), showing no indication of oligomerization in the absence of a DNA template. Circular dichroism (CD) performed on SEC-purified protein indicated a strong α-helical signal that was lost at 95 °C and completely regained after cooling, suggesting that the protein adopted the target α-helical structure (Fig. 2b).

We used static light scattering to follow the co-assembly kinetics of NX11 with various concentrations of 250 bp DNA (DNA_250_), maintaining a threefold molar excess of NX11 to DNA binding sites (assuming 1 NX repeat per bp). At the highest DNA_250_ concentration (15 ng/μL), assembly with NX11 led to a rapid increase in scattering intensity in the time between mixing and measurement, followed by a gradual decrease over ~4 h (Fig. 2c). This suggests a two-phase assembly, with a rapid nucleation driven primarily by electrostatic interactions between multiple NX11 molecules and DNA followed by a slow re-arrangement of NX11 on DNA.

Analysis of smaller NX11–DNA_48_ assemblies showed these had a hydrodynamic diameter of 18.3 nm and a polydispersity index of 0.12 as measured by dynamic light scattering (DLS) (Fig. 2d and Supplementary Fig. 2), demonstrating that NX11–DNA complexes are monodisperse and free of higher-order aggregates.

To ascertain whether the assembly of NX11 with DNA is cooperative, we assembled NX11-DNA complexes at increasing protein concentrations for a fixed concentration of DNA_250_. Electrophoretic mobility shift assays (EMSA) showed that upon increasing the excess of NX11 to DNA, mobility was reduced (Fig. 2e). At roughly threefold excess, the assemblies did not migrate into the gel due to essentially complete charge neutralization. The reduction in band intensity at higher NX11:DNA ratios is probably the result of steric hindrance or distortion of the DNA structure caused by the NX11 protein preventing the binding of Sybr Safe dye to the DNA. When analyzing denatured NX11-DNA assemblies using ~0.2% sodium dodecyl sulfate (SDS) surfactant in the loading dye, we observed full-length DNA_250_ with similar intensity as the control in all samples. A similar EMSA experiment for NX5 with DNA assembly also showed complex formation (Supplementary Fig. 3).

If NX11 assembles on DNA as intended, it would form a continuous protein coat that would be expected to protect the DNA template against nuclease activity. To test this, we assembled NX11-DNA complexes and exposed them to Benzonase in an optimal cleavage buffer for various incubation times before adding 50 mM EDTA to chelate Mg^2+^ and Ca^2+^ cofactors and terminate the reaction. EMSA performed in the presence of ~0.2% SDS revealed that full-length DNA_250_ could be observed even after overnight (>18 h) incubation with Benzonase, while the control DNA was fully cleaved in less than 1 min (Fig. 2f). These data indicate that NX11 indeed forms a tightly bound protective coating on the DNA template.

Next, to explore the importance of the NX11–NX11 interface for DNA coating and protection, we performed an alanine scan to identify key residues contributing to the interface strength (see Methods). We successfully purified a single-alanine mutant, NX11-1A (V25A), and a triple-alanine mutant, NX11-3A (V25A, L36A, L372A) (Supplementary Fig. 1). EMSA of NX11-DNA_250_, NX11-1A-DNA_250_, and NX11-3A-DNA_250_ assemblies demonstrated that the alanine mutants form more intermediate assembly species, and that complete assembly required a larger excess of protein (Supplementary Fig. 4). Benzonase challenge revealed that the mutant NX11-1A-DNA_250_ and NX11-3A-DNA_250_ complexes provided considerably less protection of their template compared to NX11 (Supplementary Fig. 5).

We also conducted electron microscopy with a N-terminal fused superfolder GFP (sfGFP) to NX11 assembled with DNA_250_ at 0.5× molar excess. While the N-terminal sfGFP fusion may introduce steric effects that alter assembly behavior, it served as an electron-dense label to unambiguously identify protein-bound regions by negative stain EM. The resulting micrographs revealed localized protein clusters along the DNA, but showed no consistent coating, suggesting that while NX11–NX11 interfaces likely form, these do not result in strong cooperative assembly (Supplementary Fig. 6). Together, these data show that the energy gain from the NX11–NX11 interface is crucial but not sufficient to drive a fully cooperative assembly.

### Structure of NX11–DNA complexes

**Fig. 3:**
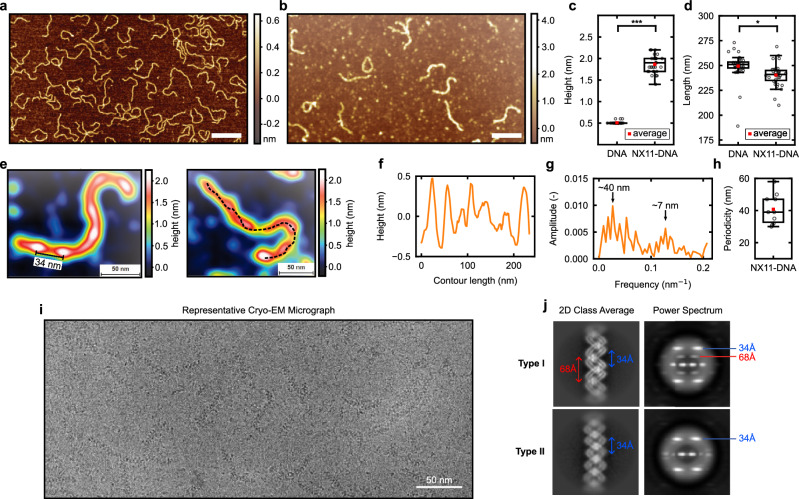
Structure of NX11–DNA complexes. **a** AFM map of DNA750. Scale bar: 200 nm. AFM imaging was independently repeated more than three times with similar results. **b** AFM map of NX11–DNA750 complexes. Excess NX11 proteins are visible in the background. Scale bar: 200 nm. AFM imaging was independently repeated more than three times with similar results. **c** Average cross-sectional height of individual DNA molecules (0.50 ± 0.05 nm) and NX11–DNA750 complexes (1.85 ± 0.40 nm). *n* = 35 individual molecules per condition, measured from a single AFM sample preparation. Statistical significance was determined by an unpaired two-tailed Student’s *t*-test with no adjustments for multiple comparisons (*p* < 0.001). The box plot center line represents the median, box bounds represent the 25th and 75th percentiles (interquartile range, IQR), and whiskers extend to Q1 − 1.5× IQR and Q3 + 1.5× IQR. **d** Average contour length of DNA (250 ± 20 nm) and NX11–DNA750 complexes (240 ± 20 nm). *n* = 35 individual molecules per condition, measured from a single AFM sample preparation. Statistical significance was determined by an unpaired two-tailed Student’s *t*-test with no adjustments for multiple comparisons (*p* < 0.05). The box plot center line represents the median, box bounds represent the 25th and 75th percentiles (IQR), and whiskers extend to Q1 − 1.5× IQR and Q3 + 1.5× IQR. **e** Zoomed in AFM images of representative individual NX11–DNA750 particles viewed by AFM. An example of a ~40 nm observed periodicity is indicated in the figure. The dotted line represents cross-sectional profiles. Representative image from a single AFM experiment. **f** Representative cross-sectional height profile of a single NX11–DNA particle, measured by AFM. **g** Representative example of the Fast Fourier Transform of the first-order derivative of a cross-sectional height profile. Two main peaks, corresponding to height periodicities on the NX11–DNA750 contours, were observed at ~40 nm and ~7 nm. **h** Analysis of *n* = 10 individual NX11–DNA750 complexes from a single AFM sample preparation showing the distribution of the main periodicity (average: 40 ± 10 nm). The box plot center line represents the median, box bounds represent the 25th and 75th percentiles (IQR), and whiskers extend to Q1 − 1.5× IQR and Q3 + 1.5× IQR. **i** Representative cryo-EM micrograph of NX11Q21.5–DNA48 complexes. Representative micrograph from a dataset of 10,595 micrographs. **j** Representative 2D class averages of two main types of complexes found (type I and type II). Type I appears to show a double-coiled assembly with apparent helical pitches of 34 Å and 68 Å, while type II only has the apparent 34 Å pitch. Pitches are determined from the X-shaped power spectra for each class average.

We used high-resolution atomic force microscopy^23^ (AFM) to characterize the structure of NX11-DNA_750_ complexes as well as the DNA_750_ template alone (Fig. 3a, b). The Z-direction noise level during imaging was 20–30 pm, sufficient to resolve height differences between DNA and NX11–DNA^24,25^. Given the tip-convoluted lateral size of DNA (~10 nm), our imaging resolution of 2 nm/pixel satisfies Nyquist-Shannon sampling criteria, ensuring accurate spatial reconstruction of molecular features.

The average cross-sectional height of DNA_750_ and NX11–DNA_750_ complexes obtained from AFM was 0.50 ± 0.1 nm and 1.9 ± 0.4 nm, respectively (Fig. 3c). These values are consistent with the expected values for cross-sectional diameters of ~2 nm for DNA and ~3 nm for NX11–DNA complexes due to the non-linear underestimation that occurs in AFM when measuring cylindrical objects with diameters smaller than ~4 nm^26^. While tip-induced deformation can affect AFM measurements, our focus was on relative height comparison. To ensure consistent deformation across samples, we used amplitude modulation AFM under phase-controlled conditions (<Δ20°), as established in previous studies^23,27–29^. This approach ensures similar energy dissipation during tip-sample contact, enabling accurate comparison.

Our quantitative single-molecule analysis demonstrated that the heights of NX11–DNA assemblies are significantly different from those of DNA_750_ alone (*p* value < 0.001), indicating that NX11 proteins are bound to the DNA. Moreover, to demonstrate that this difference is significant over the substrate roughness, we compared the line profiles of DNA and NX11–DNA samples against those of their respective surfaces (Supplementary Fig. 7a). Analyzing contour lengths revealed an average contour length of 250 ± 20 nm for DNA_750_ (3.4 Å per bp) (Fig. 3d). Contour lengths of the NX11–DNA complexes were slightly shorter than those of DNA (240 ± 20 nm; *p* < 0.05). This result indicates some level of structural rearrangement of the DNA upon NX11 binding, resulting in condensation of the DNA along the long axis of the complexes.

AFM analysis revealed that the NX11–DNA assemblies exhibited periodic fluctuations in height along the long axis (Fig. 3e). We analyzed this periodicity at the single-molecule level by acquiring height profiles along the main axis of symmetry of the NX11–DNA complexes. A first analysis of the cross-sectional profiles revealed a periodicity of ~40 nm (Fig. 3f). An accurate and unbiased analysis of the periodicity of the profiles was obtained via Fast Fourier Transform (FFT), which confirmed the presence of a major periodicity of 40 ± 10 nm and additionally revealed a smaller periodicity of 7 ± 2 nm (Fig. 3g, h and Supplementary Fig. 8). The minor periodicity (~7 ± 2 nm) likely corresponds to a single NX11 molecule, consistent with the helical pitch of one NX11 unit observed in cryo-EM. The major periodicity (~40 ± 10 nm) corresponds to approximately 5 NX11 molecules, suggesting a larger-scale repeating structural unit that may arise from a cumulative mismatch between the NX11 superhelical twist and the helical twist of the DNA template. To validate that these periodic features are specific to NX11–DNA complexes and not artifacts of the substrate or DNA alone, we performed FFT analysis on bare DNA and mica surfaces without proteins (Supplementary Fig. 7b). Consistent with previous studies^29^, these controls showed no detectable periodicity. In contrast, NX11-DNA complexes exhibited clear and reproducible periodic patterns (Supplementary Fig. 8), confirming that the observed periodicity is intrinsic to NX11 binding.

Negative stain electron microscopy (nsEM) was used to study smaller NX11–DNA complexes (<40 nm) in greater detail. When using short DNA templates (50 bp), we observed side-by-side dimerization of protein-DNA fibrils in 2D class averages. Such dimers were not observed for complexes in which longer templates (100 bp) were used (Supplementary Fig. 9). Dimerization may be due to weak yet repetitive interactions along the DNA fiber. For shorter templates, which are below the persistence length of DNA, the duplex remains relatively rigid and can engage in stackable contacts that favor dimer formation, whereas longer templates introduce flexibility that reduces the likelihood of such ordered stacking. In an attempt to block such interactions, we introduced an Arg21Gln mutation on every second repeat of NX11 (NX11^21Q.5^). Indeed, no dimerization was detected during nsEM of NX11^21Q.5^–DNA_50_ fibers. The NX11^21Q.5^–DNA_50_ 2D class averages showed a continuous polymerization of protein on DNA, as expected based on known structures of TALE–DNA complexes^17^ (Supplementary Fig. 10).

To increase resolution, we analyzed NX11^21Q.5^–DNA_48_ complexes by cryo-EM (Fig. 3i). 2D class averages clearly demonstrated heterogeneity in the particle population. Two main types of NX11^21Q.5^–DNA_48_ complexes could be distinguished: Type I complexes appeared to show a double coil (Fig. 3j), with an additional double pitch of ~68 Å relative to the expected ~34 Å pitch in the design model. Type II complexes showed only the expected ~34 Å in the class averages and a power spectrum analysis. Owing to heterogeneity within both complex types deriving from flexibility along the fiber axis, it was not possible to obtain accurate 3D reconstructions at high resolution. Nevertheless, AlphaFold2 predictions suggested that NX11^21Q.5^ could adopt an extended conformation with a helical pitch approaching 68 Å (Supplementary Fig. 11), suggesting that the double-coil assembly may be formed by similar extended polymers.

More detailed cryoEM analysis (Supplementary Fig. 12) highlights the presence of rarer distinct assembly states (Types III and IV). Type I and II assemblies account for ~50% of the total particles each, whereas Type III and IV represent <1%. Across all 2D class averages, there is a consistent absence of discernible α-helical secondary structure, suggesting that assembly occurs in a highly flexible and non-specific manner with pitches that deviate markedly from the design model.

In the absence of more detailed data, we conclude from the 2D class averages that NX11 proteins are closely packed and ordered on the DNA, suggesting that the designed protein-protein interfaces are formed. Additionally, no incomplete assemblies or assemblies of only NX11 were observed by either AFM or cryo-EM. Crucially, at high protein concentrations, we demonstrated that NX11 does not oligomerize in solution by itself (Fig. 2c), suggesting that it is indeed a conformational change upon DNA binding that triggers the assembly of NX11.

### Antigen display and immunogenicity of NX11-DNA complexes

Ordered, DNA-templated NX11–DNA fibers could be used in a range of applications where having a nucleic acid cargo and a linear, repetitive structure could be advantageous. One such example is peptide antigen display for vaccine applications. The linear NX11–DNA structures should allow for presentation of peptide epitopes at high yet controlled density, with antigen copy number determined exactly by DNA template length.

**Fig. 4:**
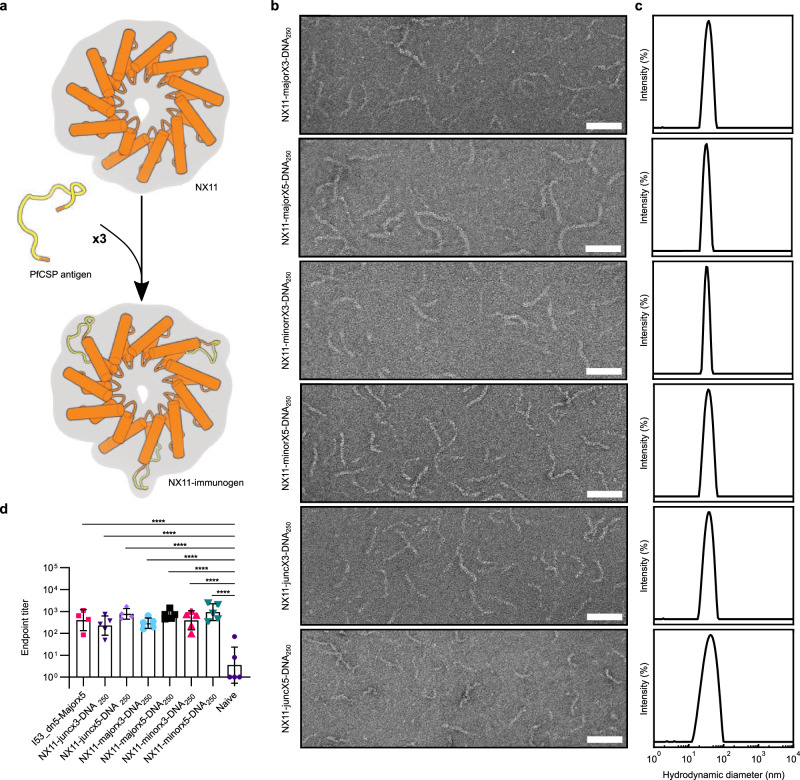
Antigen display and immunogenicity of NX11–DNA complexes. **a** Schematic of display of PfCSP tetrapeptide repeat epitopes at three locations in the NX11 protein. Repeats (×3 or ×5) of major, minor, and junctional (“junc”) epitopes were inserted in the surface-exposed loops of NX11. **b** Representative nsEM micrographs (scale bar = 100 nm). Representative micrograph selected from multiple images acquired per condition. **c** DLS of NX11–DNA250 immunogen complexes, showing a hydrodynamic diameter of ~20–30 nm. The hydrodynamic diameters, polydispersity index, and autocorrelation functions are presented in Supplementary Fig. 14. **d** Endpoint titers (OD = 1.0) of anti-PfCSP antibody in the sera of immunized mice (*n* = 5 biological replicates per group), measured by ELISA. Each immunogen group was compared to the naïve group. Data are presented as mean values ± s.d. Statistical significance was determined by ordinary one-way ANOVA with Tukey’s multiple comparisons test (****p < 0.0001).

To explore this application, we chose three peptide epitopes derived from the immunodominant repeat region of the *Plasmodium falciparum* circumsporozoite protein (PfCSP)^30^. Rosetta Remodel was used to model three and five copies of the major, minor, and junctional (junc) repeats of PfCSP inserted into the 3rd, 6th, and 10th surface loops of NX11 (majorX3, minorX5, etc.; Supplementary Table 2). This arrangement would display the epitopes with roughly equal spacing on the DNA (Fig. 4a). All NX11 immunogens were successfully purified by IMAC and SEC. The proteins were assembled with highly pure pharmaceutical-grade DNA_250_ (NoLimits, Thermo Fisher Scientific) into complexes at a 1.1× excess, and assembly was confirmed by EMSA (Supplementary Fig. 13). nsEM micrographs showed NX11–DNA_250_ complexes were formed and appeared reasonably homogenous (Fig. 4b). DLS measurements of the complexes yielded hydrodynamic diameters of 20–30 nm, which is in line with rod-shaped particles of ~6 nm diameter and ~85 nm length (Fig. 4c, Supplementary Fig. 14).

In previous work, computationally designed protein nanoparticles have been successfully used as multivalent display platforms for vaccine development^31–35^. To benchmark the performance of our linear protein–DNA immunogens against one of these, we engineered majorX5 into surface-exposed loops of the homopentameric component of a de novo two-component icosahedral nanoparticle (I53_dn5_majorX5), such that each particle displayed 60 copies of majorX5 antigen (Supplementary Fig. 15).

We immunized groups of five B6(Cg)-*Tyrc-2J*/J mice intramuscularly with 1.5 µg of the NX11–DNA_250_ immunogens or the I53_dn5_majorX5 benchmark immunogen formulated in AddaVax at weeks 0 and 3. Serum collected two weeks post-boost was analyzed for PfCSP binding by enzyme-linked immunosorbent assay (ELISA), which showed antigen-specific antibody was elicited by all immunogens (Fig. 4c). Because NX11, DNA, or the naked NX11–DNA complex are not expected to elicit PfCSP-specific immune responses in the absence of an antigen, and unassembled peptide would likely be weakly immunogenic^36^, we concentrated on comparing the antigen-displaying constructs and included benchmark and naïve groups to confirm the specificity of the response. Importantly, the NX11–DNA immunogens elicited similar antibody titers to the de novo icosahedral protein nanoparticle, indicating that the linear NX11–DNA platform shows potential as a multivalent vaccine platform (Fig. 4d).

## Discussion

In this study, we successfully redesigned a naturally occurring TALE protein to assemble into linear polymers on dsDNA templates. We leveraged the repetitive nature of the CRD domain by eliminating the naturally occurring capping domains and reusing the inter-repeat interface as a protein-protein interface. This approach is relatively straightforward and could be used to generate assemblies from other types of repeat proteins, which have proven to be versatile building blocks for designing new protein-based materials with high precision^32,37–39^. For instance, a similar strategy was recently employed to create protein building blocks that form a wide variety of architectures without extensive sampling of irregular interactions^37^. Our work extends this principle to templated assembly on nucleic acids, thereby providing a route to combining the accurate design of self-assembling proteins with DNA nanotechnology^40^.

Electron microscopy of NX11–DNA fibrils revealed a periodicity of ~68 Å in addition to the expected pitch of ~34 Å, which was confirmed here by AFM single-molecule analysis. Thus, EM and AFM suggested that two NX11 fibrils may wrap the DNA. The periodicity of around ~68 Å is consistent with the AF2-predicted pitch for the unbound case, and there is experimental evidence suggesting that non-bound or non-specifically bound TALEs can adopt configurations with a longer superhelical pitch. For example, for the dHax3 TALE, the superhelical pitch increases from 35 Å in the DNA-bound form (PDB ID: 3V67) to ~60 Å in DNA-free form (PDB ID: 3V6P)^22^. There is some evidence to suggest that TALEs first search DNA in a weakly bound long-pitch configuration, followed by a shortening of the pitch when binding more strongly to their target sequence^15,41^. We hypothesize that our NX11 proteins possess conformational flexibility similar to that observed in natural TALE proteins. Because NX11 exhibits plasticity in its super-helical pitch, allowing it to stretch and match the DNA double-helical pitch of ~64 Å, skipping every second major groove. This would create enough space for the formation of a two-start double-coil assembly.

Natural TALEs show bi-directional diffusion along the DNA during their target search mode^41^. For NX11, assemblies likely terminate when colliding fibers of opposing directionality meet. This could act as a nucleation point for the elongation of the NX11 along the DNA in either direction. Since this assembly process is stochastic and requires structural adaptation, this could introduce local defects, which we hypothesize complicates high-resolution structural studies when bound to DNA, especially for longer DNA sequences. A slight mismatch between the NX11 superhelical twist and the DNA helical twist would progressively build up frustration along the assembly, introducing periodic defects at regular intervals. This is consistent with the ~40 nm major periodicity observed by AFM, and likely also contributes to the structural heterogeneity that has prevented high-resolution 3D reconstruction by cryo-EM.

Another cause for heterogeneity could be the DNA sequence itself. Although the NoLimits DNA template used here has an undisclosed sequence, DNA sequence-dependent variations in B-form geometry—such as altered twist, roll, and groove width in pyrimidine-rich tracts or methylated regions—are well documented^42–44^ and could plausibly contribute to the deformations we observed in NX11–DNA complexes.

As we demonstrated, linear protein–DNA complexes can be an interesting modality for the precisely controlled symmetrical display of antigens at high copy numbers. In earlier studies, designed self-assembling protein nanoparticles symmetrically displaying heterologous antigens proved highly immunogenic^33,34^, and we found that NX11–DNA-based immunogens were similarly immunogenic. In contrast to those finite (i.e., bounded) assemblies, NX11–DNA complexes of many different sizes and antigen copy numbers could be generated by using different protein variants or simply altering dsDNA template length. Linear display platforms characterized to date include simple peptide-based systems^7,10,45,46^ and complex assemblies based on filamentous viruses^47–49^. Due to their modular nature and the high density of exterior-facing loops, the NX11–DNA assemblies designed here provide precise and tunable control over antigen spacing, as well as the ability to include various DNA templates that could provide a beneficial adjuvant effect^50^.

Direct assessment of NX–DNA complex stability in vivo remains technically challenging, and we anticipate that future studies, including exposure to serum, competing DNA-binding agents, and physiological ionic strength, will be needed to address this important aspect of translational development. Including additional controls in these studies, such as unassembled NX-epitope monomers and naked NX11–DNA complexes, will be valuable for quantifying the specific contribution of DNA-templated multivalent display to immunogenicity.

Lastly, we propose that variants of NX11 could be designed to ensure that nucleation occurs at specific DNA sequences by reinstating the RVD mutations, followed by elongation through the addition of sequence-independent binders or subunits (Supplementary Fig. 16 and Supplementary Fig. 17). This refinement would enable more precise control over the assembly process and further functional optimization of the designed assemblies. In concept, this strategy could allow for the creation of programmable and addressable hierarchical assembly^51,52^ and complex asymmetrical protein assemblies that use DNA as a template^53^.

## Methods

All animal experiments were conducted in accordance with the University of Washington’s Institutional Animal Care and Use Committee (IACUC) under approved protocol 4470-01.

### Computational design of NX-proteins

The 34 amino acid CRD protamer with the sequence PDQVVAIASNGGKQALETVQRLLPVLCQAHGLT was extracted from the PthoX1 crystal structure (PDB ID: 3UGM). The protamer was aligned to idealized B-form dsDNA^54^. Next, we enforced radial symmetry following the major groove of dsDNA (34 Å pitch, and 10.5 monomers per turn) and generated a symmetry definition file. Next, we used Rosetta XML scripting for redesign. Existing native non-specific DNA interactions, Gln14 and Lys15, were prevented from repacking. But other residues except for Gly and Pro near the RVD (and thus the DNA) were allowed to be symmetrically redesigned, with constrained backbone movements, resulting in S9K and N10R mutations. A second step of Rosetta design focused on optimizing idiosyncrasies in the monomer. Conservative Rosetta design of surface residues led to Cys27Thr, Val25Glu, and Ala29Lys mutations. All other residues were repacked during design (except for DNA-interacting residues). The symmetrically arranged monomers were oligomerized by fusing loops at 34 and position 1 of the next monomer using Rosetta remodel^20^. Oligomers with repeat numbers of 5, and 11 repeats were generated by propagation of torsion angles. Final protein sequences are provided in Supplementary Table 1.

For the design of NX11, displaying *pf*CSP immunogens (Supplementary Table 2), the immunogens replaced surface-exposed loops (HGLT) of the NX repeats at positions at repeat numbers 3, 6, and 10 of NX11. Using Rosetta Remodel, immunogen consequences were inserted at 114–118, 231–235, and 345–349 of NX11, and two adjacent residues were also mutated to Gly to provide additional flexibility. Immunogen sequences were inserted, and viability was tested by simulating 100 closures with Rosetta Remodel, while keeping the rest of the protein structure fixed. Final protein sequences are provided in Supplementary Table 1.

### Protein expression and purification

Synthetic genes for individual components, each with an N-terminal TEV protease cleavage site hexahistidine purification tag (MGHHHHHHGSSENLYFQGS) and a C-terminal Trp to facilitate UV/vis quantification. Genes were codon optimized for *E. coli* expression and purchased as genes from Genscript, ligated into the pET-29b(+) vector at the NdeI and XhoI restriction sites, or as g-blocks from Twist Bioscience Corp. and assembled using Golden Gate Assembly Mix (New England Biolabs) into a modified pET-24(+) vector. The proteins were expressed in BL21(DE3) (New England Biolabs) in Luria Broth (10 g Tryptone, 10 g NaCl, 5 g yeast extract) in 2 L baffled shake flasks. Cells were grown at 37 °C to an OD_600_ ~ 0.6, and induced with 1 mM Isopropyl ß-D-1-thiogalactopyranoside (IPTG; Sigma Aldrich). Expression temperature was reduced to 18 °C, and the cells were shaken for ∼18 h. The cells were harvested and lysed by sonication using a Qsonica Q125 for 15 min with 2 s pulses at 80% amplitude in 50 mM Tris pH 8.0, 400 mM NaCl, 30 mM imidazole, 1% Triton X-100, 10% glycerol, and 1 mM PMSF. Lysates were clarified by centrifugation at 30,000*g* for 30 min and applied to a 5 mL column bed of Ni-NTA resin (Qiagen) for purification by IMAC. Resin was washed with 25 mL wash buffer I (50 mM Tris pH 8.0, 400 mM NaCl, 30 mM imidazole, 10% glycerol) or wash buffer I + 0.75% CHAPS for proteins used in animal studies to reduce endotoxins, followed by 25 mL wash buffer II (50 mM Tris pH 8.0, 2 M NaCl, 30 mM imidazole, 10% glycerol) and 25 mL wash buffer I. The protein of interest was eluted using a gradient of 50 mM Tris, pH 8.0, 400 mM NaCl, 300 mM imidazole, 10% glycerol. During elution, absorbance at 280 nm was monitored, and ~20 mL eluates were pooled and dialyzed against 5 L phosphate saline buffer (PBS), pH 7.4. Dialysate was concentrated in 10 K MWCO centrifugal filters (Amicon) to 1.5–2 mg/mL, sterile filtered (0.22 μm), and applied to either a Superdex 200 Increase 10/300 (Cytiva) with calibration shown in Supplementary Fig. 18, or Superdex 75 10/300 (GE Healthcare) using PBS pH 7.4 or TRIS-buffer saline (TBS) pH 8.0 as the running buffer. NX-immunogens used in the mice study (i.e., NX11-majorX3) were tested for endotoxin levels <2 EU/mg using a Limulus amebocyte lysate assay (Charles River).

### Circular dichroism

Proteins were diluted to 0.15 mg/mL in PBS in a quartz cuvette (Hellma) with a 1 mm pathlength. On a JASCO J-715 (JASCO Corporation), spectral scans were averaged over 20 measurements with a scan rate of 50 nm/min and a response time of 2 s. Scans were performed at 20 °C, 95 °C, and after cooling back to 20 °C (20 °C rev). After reaching the temperature, the samples were incubated for >10 min to equilibrate. Data with high tension above 600 V is not shown.

### Assembly of NX–DNA complexes

Pharmaceutical grade DNA (NoLimits, Thermo Fisher Scientific)—unless stated otherwise—assembled at 15 ng/µL with NX5 or NX11 proteins at 3× excess relative to binding sites on DNA (assuming 1 NX repeat per bp) in PBS pH 7.4 or TBS pH 8.0. The assembly was allowed to complete for > 4 h at room temperature, and typically overnight at room temperature.

### Light scattering

NX11–DNA_250_ complexes were assembled in a 12 µl DLS quartz cuvette, and light scattering was recorded immediately. Light scattering was measured using a ZS-Nanosizer instrument (Malvern, UK) at a fixed angle of 173° and 20 °C. Measurements were averaged over 10 s, and the derived count rate (the raw scattering value corrected for X) was recorded using Zetasizer software version 7.13 (Malvern, UK). The ZS-Nanosizer instrument was also used to quantify the hydrodynamic diameter of NX11–DNA_48_ complexes using dynamic light scattering (DLS). Light scattering was measured at a fixed angle of 173° in a quartz cuvette at 20 °C. Hydrodynamic diameters were calculated based on the average of 50 measurements, with automatically determined optimal measuring settings by the Zetasizer software (version 7.13).

### Electron-mobility shift assay

A 1% agarose gel containing 1× Sybr Safe staining (New England Biolabs) was cast. 15 µl NX11–DNA_250_ complexes were mixed with 3 µl 6× loading dye (Thermo Fisher Scientific). In the case of loading complexes under denaturing conditions, complexes were mixed with 3 µl loading dye supplemented with 0.6% SDS (0.2% SDS final concentration). Agarose gel was run in 1× Tris–acetate–EDTA (Sigma Aldrich) buffer at 110 V for ~30 min and imaged using a GelDoc imager (Bio-Rad).

### Nuclease protection assay

NX11–DNA_250_ assemblies (2.25 µg DNA_250_ in 150 µL) were formulated in a 1× reaction buffer (10 mM Tris pH 7.5, 2.5 mM MgCl_2_, 0.1 mM CaCl_2_), and 12.5 U Benzonase (Millipore, Cat. No. 70746) was incubated 1 min, 5 min, 30 min, 1 h, 3 h, and overnight (~18 h). Benzonase retains >90% activity for several months under these conditions^55^. The reaction was stopped by adding 50 mM EDTA, pH 7.4, to chelate Mg^2+^ and Ca^2+^. Complexes were mixed with loading dye (Thermo Fisher Scientific) supplemented with ~0.2% SDS. Denatured complexes were run on a 1% agarose gel containing 1× Sybr Safe stain (New England Biolabs).

### Atomic force microscopy

DNA_750_ and NX11–DNA_750_ samples were prepared on positive functionalized mica substrates. To functionalize the surface, first cleaved the mica surface by exfoliating the top layer using adhesive tape, then we incubated it for 1 min with 10 µl of 0.1% (v/v) 3-aminopropyl-triethoxysilane (APTES; Sigma Aldrich) in Milli-Q water. The substrate was rinsed three times with 1 mL of Milli-Q water and dried with a gentle stream of nitrogen gas. Subsequently, 10 µl 0.3 ng/µl of DNA and NX11-DNA complexes in 1.5 mM Tris-HCl pH 8.0, were deposited onto the positive functionalized surface. The droplet was incubated for 10 min, rinsed with 1 mL of Milli-Q water, and dried by a gentle stream of nitrogen gas. The entire preparation process was conducted at room temperature and under laminar flow.

AFM maps of 3D morphology were acquired for both samples in a regime of constant phase change, with 2 nm/pixel resolution by means of a Multimode-8 (Bruker) operating in tapping mode and equipped with a gold-coated probe (HQ:NSC14/Cr-Au BS, 5 N/m; Bruker) with a nominal radius <8 nm and stiffness of 4.4 ± 0.1 N/m as estimated by the thermal sweep method. The cantilever’s free oscillation amplitude was approximately 30–35 nm, with an amplitude setpoint of 20–25 nm (corresponding to 70–80% of the free amplitude).

Scanning probe image processor (version 6.7.3, Image Metrology, Denmark) software was used for image processing and further analysis. Cross-sectional profiles of both DNA and NX11–DNA were extracted for statistical analysis at the level of individual molecules (*n* = 35). Additionally, a FFT of the cross-sectional profile and the gradient of the cross-sectional profile were used to derive variations in periodicity for NX11–DNA molecules (*n* = 10), allowing for observation of higher and smaller periodicity components.

### Negative stain electron microscopy collection and processing

NX11–DNA was diluted to 12 ng/μL DNA concentration prior to application of 3 μL of sample onto freshly glow-discharged 400-mesh copper grids (Ted Pella). The sample was incubated on the grid for 15–30 s before excess liquid was blotted away with filter paper (Whatman). 3 μL of 2% w/v uranyl formate (UF) stain was applied to the grid and immediately blotted away before an additional 3 μL of UF stain was applied. The stain was blotted off by filter paper, and a final 3 μL of UF stain was applied and allowed to incubate for ~30 s. Finally, the stain was blotted away, and the grids were allowed to dry for 3 min. Prepared grids were imaged using EPU 2.0 on a 120 kV Talos L120C transmission electron microscope (Thermo Fisher Scientific) at 72,000× magnification with a BM-Ceta camera. Data processing was done in CryoSPARC^56^, starting with CTF correction, particle picking, and 2D classification.

### Cryo-EM data collection

Automated data collection, totaling 10,595 movies, was performed using SerialEM at a calibrated pixel size of 0.4135 Å in super-resolution mode. Movies were recorded as 75-frame stacks with a total accumulated dose of approximately 60 e^−^/Å².

### Image pre-processing and 2D classification

All data processing was performed in CryoSPARC. Movie frames were corrected for beam-induced motion using patch motion correction, followed by CTF estimation using patch CTF estimation with default parameters.

Filament segments were automatically picked using the filament tracer, yielding an initial dataset of 1,363,928 particles. Extracted particles were subjected to iterative rounds of 2D classification to remove poorly aligned segments and to sort particles into structurally homogeneous classes.

Particles were grouped into four distinct assembly classes based on 2D class averages and corresponding power spectra: Type I (795,040 particles, approximately 58%), Type II (568,888 particles, approximately 42%), and two rare populations identified during subsequent iterative rounds of 2D classification, Type III and Type IV assemblies (representing less than 1% of the particle population). For each class, representative 2D class averages were used to compute averaged power spectra in CryoSPARC to estimate helical pitch and characterize structural periodicity.

### 3D reconstruction and model-guided refinement

Three-dimensional reconstructions were performed exclusively in C1 symmetry, and with no helical symmetry was applied at any stage. Multiple symmetry search attempts were conducted but did not yield consistent or interpretable solutions and were not pursued further, likely due to the heterogeneity and flexibility of the particles in this dataset.

Ab initio reconstruction jobs were performed in CryoSPARC using particle subsets corresponding to either Type I or Type II 2D class averages. These reconstructions yielded two distinct low-confidence 3D architectures associated with the dominant assembly classes. For Type II assemblies, heterogeneous refinement was carried out in C1. AlphaFold3 modeling for Fig. S12B, C was performed using four copies of NX11 in complex with a 100 bp DNA construct. Predicted double-coil assemblies were used as reference models in both parallel and anti-parallel configurations relative to the DNA axis for Fig. S12K, L. AlphaFold3-derived models were further refined by real-space fitting and relaxation into the cryo-EM density using ISOLDE to better account for the observed curvature of the assembly in Fig. S12J.

Despite extensive ab initio reconstruction and heterogeneous refinement attempts, the 3D maps generated in this study did not reach sufficient resolution or consistency to support atomic model building or deposition.

### Antigen-adjuvant formulations and immunizations in mice

NX11-immunogens (NX11-majorX3, NX11-majorX5, NX11-minorX3, NX11-minorX5, NX11-juncX3, NX11-juncX5) were assembled with pharmaceutical-grade DNA_250_ (NoLimits, Thermo Fisher Scientific) at a DNA concentration of 10.33 ng/μL and 1.1-fold excess of protein (assuming 1 NX protein binding to 1 bp) in TBS + 5% glycerol. The 10% excess NX11 protein was not further purified from the complexes. The Complexes were incubated overnight at room temperature, split into 12 × 5 µL, and flash frozen in −80 °C.

Directly before injection, I53_dn5_majorX5 and NX11-DNA immunogen aliquots were thawed on ice, and 1.5 µg was mixed with Addavax adjuvant in a 1:1 ratio. This vaccine formulation (in a volume of 100 µL) was injected intramuscularly in the hind legs of the mice (5 mice per group).

### I53_dn5_majorX5-L1 protein expression and purification

I53_dn5A.1_majorx5-L1 component containing a His-tag was expressed in a pET29b+ vector in BL21(DE3) *E. coli*. Inoculated cultures were expressed in Terrific Broth II (MP Biomedicals) in 2 L baffled flasks at 37 °C and 220 RPM until OD600 reached 0.6–0.8. Cultures were then induced with 1 mM IPTG, and the temperature was lowered to 18 °C and grown for 16–18 h. Cells were harvested by centrifugation and lysed by high-pressure microfluidization.

Clarified *E. coli* lysate was then applied to Nickel–NTA gravity (QIAGEN) flow column that was previously equilibrated with 50 mM Tris, 150 mM NaCl, 20 mM Imidazole, pH 8.0 (Buffer A). After sample application, the column was washed with 5 CV of Buffer A, and then with 10 CV of Buffer A + 0.75% CHAPS for endotoxin removal, and once again with 5 CV of Buffer A. Protein was eluted with 50 mM Tris, 150 mM NaCl, 300 mM Imidazole, pH 8.0 (Buffer B). The eluted fractions were then analyzed by SDS-PAGE to confirm the presence and purity of I53_dn5A.1_majorx5-L1. IMAC purified protein was then further purified by SEC using a Superdex 200 Increase 10/300 (Cytiva) equilibrated in 50 mM Tris, 150 mM NaCl, 5% Glycerol. Fractions were collected, pooled, and analyzed by SDS-PAGE to confirm purity, UV/vis spectroscopy to obtain protein concentration, and Limulus amebocyte lysate assay (Charles River) for endotoxin.

### Enzyme-linked immunosorbent assay (ELISA) for measuring binding antibody titers

96-well flat-bottom Immuno MaxiSorp plates were coated with 20 ng of *pf*CSP antigen per well in 100 µL of PBS buffer overnight at 4 °C. Coated plates were washed 3× with PBS with 0.05% Tween-20 (ELISA wash buffer). Plates were blocked with 5% Casein in ELISA wash buffer (blocking buffer) for 1 h at room temperature, and plates were subsequently washed 3× with ELISA wash buffer. Mouse sera was individually diluted 1:100 in blocking buffer and then diluted fourfold down the plate to a final dilution of 1.05E + 08 and incubated at room temperature for 45 min. Plates were washed, and then Goat anti-mouse IgG-HRP was diluted to 1:2000 in ELISA wash buffer and added to the plates for 45 minutes at room temperature. Plates were washed a final time, and 100 µL of TMB 1-component peroxidase substrate (SeraCare) was added for 5 min and subsequently neutralized with 2 N HCl. Absorbance at 450 nm was measured with a Neo2 plate reader to determine endpoint values.

### In vivo immunogenicity

Female B6(Cg)-*Tyrc-2J*/J mice were purchased from The Jackson Laboratory (strain code 000058) at 6 weeks of age. Mice were housed in a specific-pathogen-free facility within the Department of Comparative Medicine at the University of Washington, Seattle, accredited by the Association for Assessment and Accreditation of Laboratory Animal Care (AAALAC). Animal studies were conducted in accordance with the University of Washington’s Institutional Animal Care and Use Committee under protocol 4470-01. For each immunization, low-endotoxin immunogens were diluted in buffer and mixed with 1:1 v/v AddaVax adjuvant (InvivoGen vac-adx-10) to obtain a final dose of 1.5 μg of immunogen per animal, per injection. At 8 weeks of age, 5 mice per group were injected intramuscularly in the quadriceps with 50 μL of immunogen per hind leg at weeks 0 and 3. Animals were bled using the submental route at weeks 2 and 5. Whole blood was collected in serum separator tubes (BD #365967) and rested for 30 min at room temperature for coagulation. Tubes were then centrifuged for 10 min at 2000×*g*, and serum was collected and stored at −80 °C until use.

### Biolayer interferometry

Biolayer interferometry (BLI) binding assays were performed in duplicate on an Octet RED96 instrument (ForteBio) at 25 °C, shaking at 1,000 rpm. Measurements were performed in binding buffer: 25 mM Tris (pH 7 for NS20, or pH 8 for NS14), 150 mM NaCl, 0.5 w/v % Bovine Serum Albumin (BSA), 0.5 v/v % Tween-20. Depending on whether specific or random binding was being analyzed, and only in the association and dissociation steps, 10 mM MgCl_2_. Streptavidin (SA) tips (ForteBio) were hydrated for 10 min in the binding buffer without MgCl_2_. The tips were then loaded with biotinylated duplex DNA (Integrated DNA Technologies) at 100 nM until an appropriate threshold was reached, followed by a baseline for 60 s. The sensors were then immersed in a 1:2 serial dilution of the protein of interest for 300 sec, followed by a dissociation phase of 300–1500 s. Curve fitting was performed using a 1:1 or 1:2 stoichiometry binding model, where appropriate, using the ForteBio data analysis software. Dissociation constants were determined using global fits.

## Supplementary information

Supplementary Information

Transparent Peer Review file

## Source data

Source Data

## Data Availability

The computational model coordinates and design scripts generated in this study have been deposited on Zenodo (10.5281/zenodo.19547408)^57^. Protein sequences are provided in Supplementary Tables 1 and 2. Source data for figures are provided in the Source Data file. The crystal structure used as a starting point for design is available in the Protein Data Bank under accession code 3UGM. Source data are provided with this paper.

## Code availability

Rosetta design scripts used in this study are available on Zenodo (10.5281/zenodo.19547408)^57^.
