# Supplementary material for: Redesign of TALE proteins for DNA-templated assembly of protein fibers
Source: Nat Commun. 2026 May 19;17:6582. doi: 10.1038/s41467-026-73313-8 (PMC13381548; doi:10.1038/s41467-026-73313-8)
Supplement: Supplementary file 1 — Supplementary Information [file 41467_2026_73313_MOESM1_ESM.pdf]

Supplementary Information

Redesign of TALE proteins for DNA-templated assembly of protein fibers

Robbert J. de Haas[1], Mark D. Langowski[2,3], Andrew J. Borst[2,3], Visakh V. S. Pillai[1], Gwendolyn E. Hoffmann[1], Martin Bongers[1], Matthias Mulder[1], Suna Cheng[2,3], Catherine Treichel[2,3], Elizabeth M. Leaf[2,3], Mengyu Wu[2,3], Eric M. Lynch[2], Justin M. Kollman[2], Francesco S. Ruggeri[1,4], Carl Walkey[2,3], Renko de Vries[1,*], Neil P. King[2,3,*]

[1] Department of Physical Chemistry and Soft Matter, Wageningen University & Research, Wageningen, The Netherlands
[2] Department of Biochemistry, University of Washington, Seattle, WA, USA
[3] Institute for Protein Design, University of Washington, Seattle, WA, USA
[4] Department of Organic Chemistry, Wageningen University & Research, Wageningen, The Netherlands

* Corresponding authors. Email: renko.devries@wur.nl, neilking@uw.edu

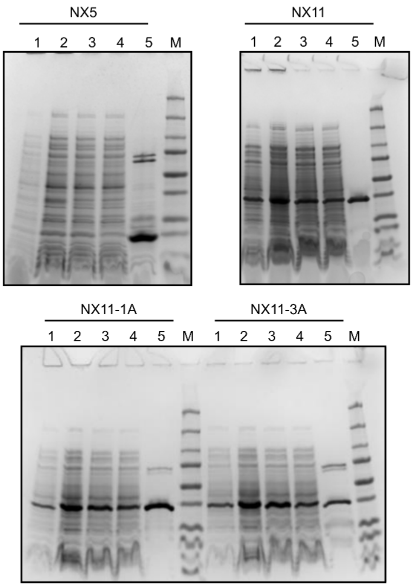

Supplementary Figure 1. SDS-PAGE of purification of NX5, NX11, NX11-1A, and NX11-3A proteins from *E. coli.*

1) Induced cell pellet, 2) cell lysate, 3) clarified lysate, 4) IMAC flow-through, and 5) IMAC eluate. M) Precision Plus Protein Dual Color Standard (250 kDa, 150 kDa, 100 kDa, 75 kDa, 50 kDa, 37 kDa, 20 kDa, 15 kDa, 10 kDa; Bio-Rad). The experiment was independently repeated more than five times with similar results.

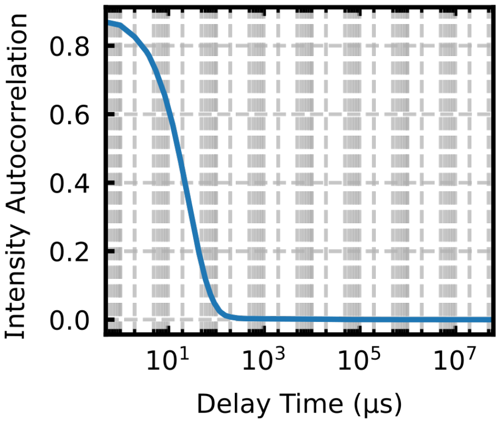

Supplementary Figure 2. Raw average autocorrelation vs. delay time for data presented in Fig. 2d.

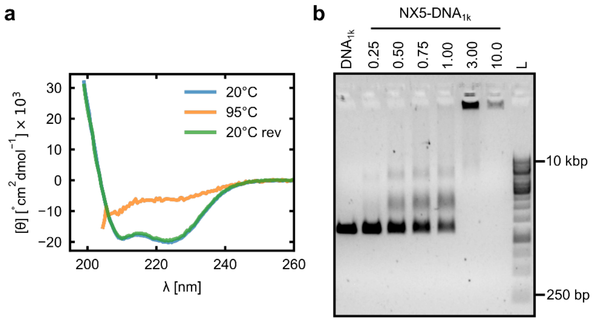

Supplementary Figure 3. Biochemical characterization and assembly of NX5 with DNA.
(a) Circular Dichroism of purified NX5 in PBS. Molar residue ellipticity [θ] is plotted as a function of wavelength. NX5 was largely unfolded at 95 °C, but upon cooling back to 20 °C (20 °C rev) re-folded to its original conformation. **(b)** EMSA showing co-assembly of NX5 with 15 ng/μL DNA_{1k} at various NX5:DNA_{1k} ratios. Due to charge neutralization and increased size, the NX5-DNA complexes migrated slower, indicating assembly. The DNA_{1k} lane contained a DNA-only sample. L was a 1 kb gene ruler (ThermoFisher) with minimal and maximal size indicated.

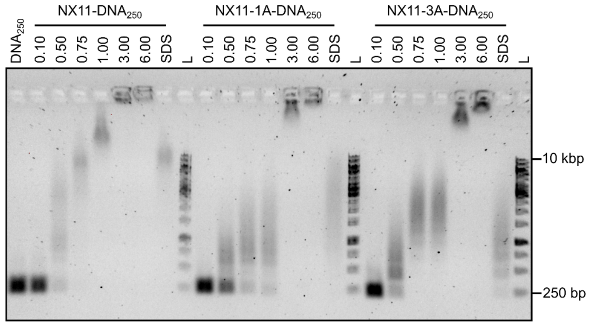

Supplementary Figure 4. EMSA comparing co-assembly of NX11, NX11-1A, and NX11-3A with DNA_{250} at various ratios of protein to DNA.

All three proteins fully assembled at excess above 3.00. However, alanine interface mutants NX11-1A and NX11-3A appeared to shift at higher excess of protein:DNA, indicating that weaker interfaces result in a less stable assembly. The “SDS” lane contained a 3-fold excess assembly of NX11-DNA loaded in presence of ~0.2% SDS to denature the complexes. In all cases the complexes were not fully denatured as they appear to smear on the gel. “L” was a 1 kb gene ruler (ThermoFisher) with minimal and maximal size indicated.

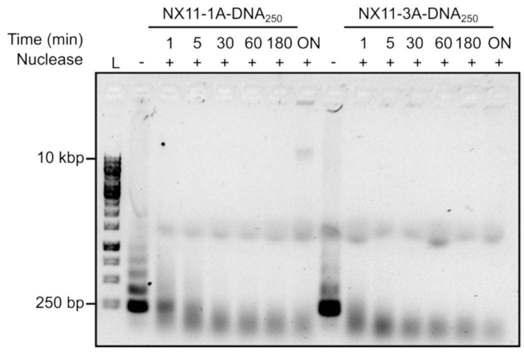

Supplementary Figure 5. Benzonase assay of NX11-1A and NX11-3A assembled with DNA_{250}. Benzonase nuclease was added to the protein-DNA complexes in reaction buffer (10 mM Tris pH 7.5, 2.5 mM $MgCl_2$, 0.1 mM $CaCl_2$) and incubated for 1 min, 5 min, 30 min, 60 min, 180 min, and overnight (ON). A control DNA-only sample lacking Benzonase was loaded as reference. Benzonase cleavage was stopped by addition of a stopping buffer (50 mM EDTA pH 7.4). Samples were loaded in the presence of 0.2% SDS to fully denature the complexes and observe if the 250bp template DNA was cleaved. This appeared to be the case for both NX11-1A and NX11-3A: compared to the DNA-only sample, less intense bands were observed and the DNA was shorter than 250bp. “L” was a 1 kb gene ruler (ThermoFisher) with minimal and maximal size indicated.

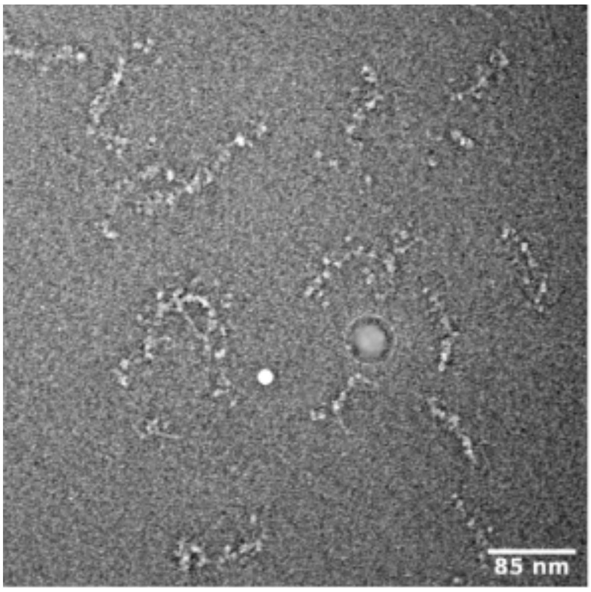

Supplementary Figure 6: Negative stain electron micrograph of N-terminal fused superfolder GFP (sfGFP) to NX11 protein (sfGFP-NX11) complexed with 250 bp DNA (1.5 ng/μL) assembled at 0.5X molar excess in PBS and incubated overnight at room temperature. Representative micrograph from a single experiment.

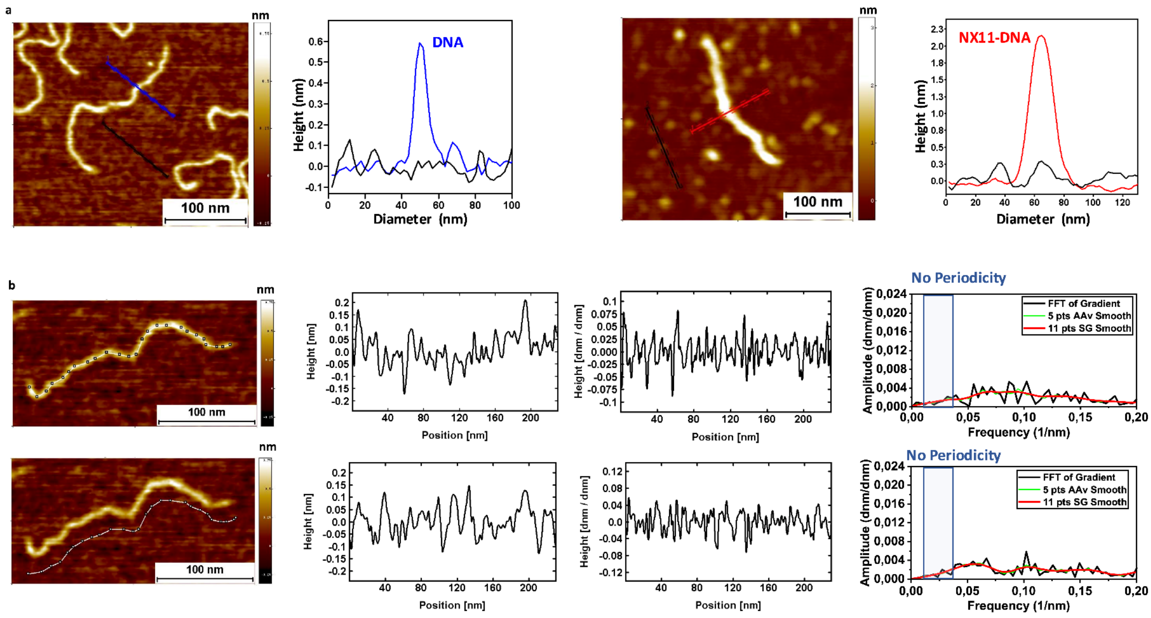

Supplementary Figure 7. (a) Line profiles on the mica substrate for both DNA and NX11-DNA samples. **(b)** FFT analysis on DNA and bare mica without proteins.

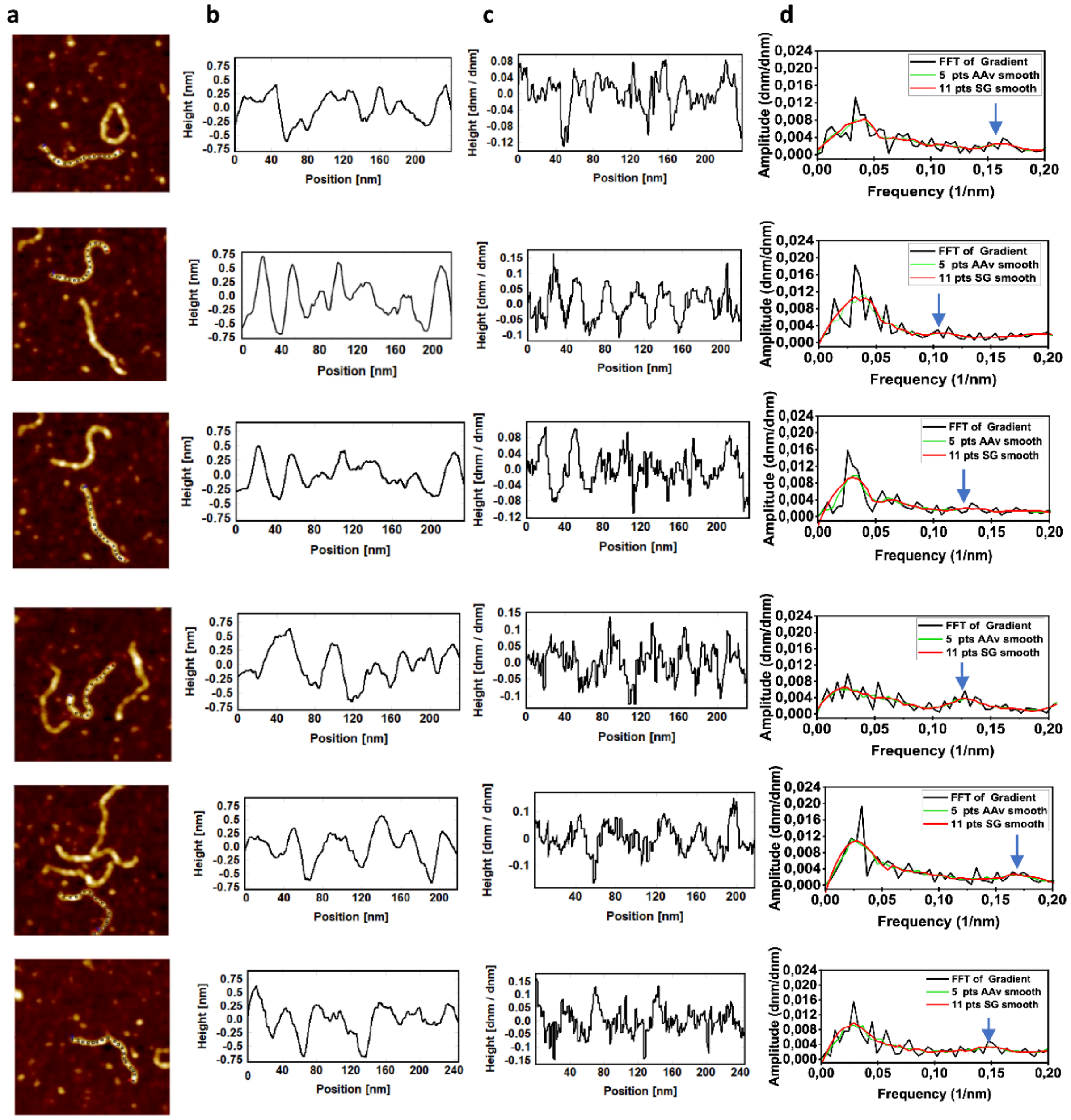

Supplementary Figure 8. AFM periodicity analysis traces n = 6 individual NX11-DNA particles. **(a)** AFM morphology maps, **(b)** line profiles, **(c)** gradients of the line profiles, and **(d)** fourier transforms of the gradient profiles. Blschue arrow indicates the minor periodicity corresponding to ~7 nm.

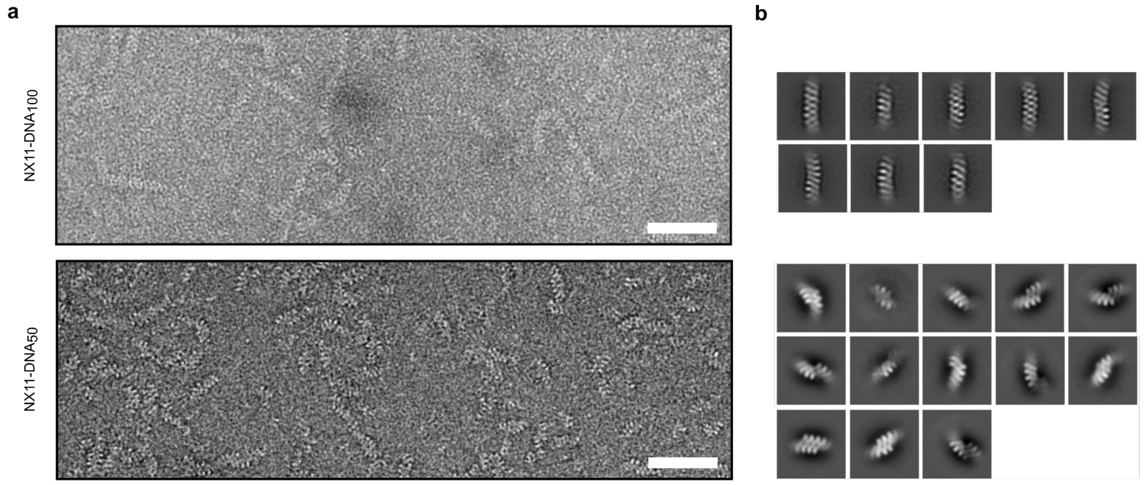

Supplementary Figure 9. nsEM of NX11-DNA$_{100}$ and NX11-DNA$_{50}$ complexes.
(a) Representative negatively stained electron micrographs. Both assemblies produce fibrous complexes of the approximate expected contour length. Representative micrographs from a single experiment. **(b)** 2D class averages demonstrate that in the case of shorter DNA lengths (< 100 bp), NX11-DNA complexes tend to stack side-by-side as can be seen from a selected subset of 2D class averages. In the case of DNA > 100 bp, complexes do not stack side-by-side, likely because they are less rigid. Scale bars: 50 nm.

a

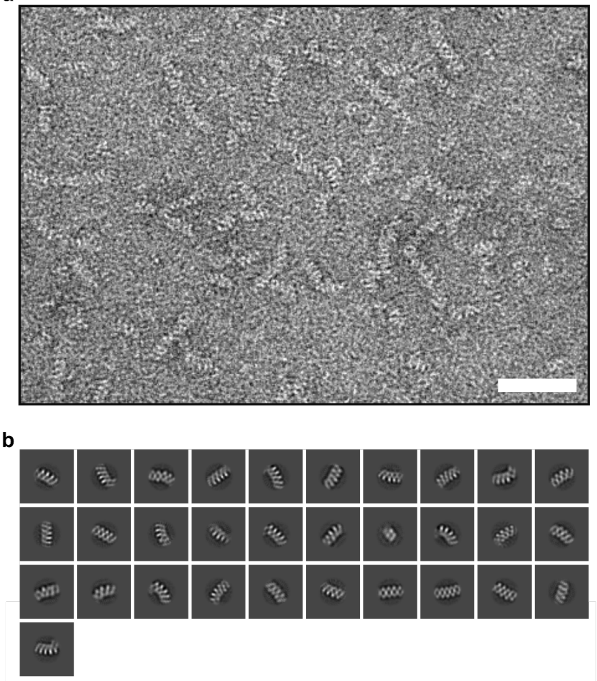

Supplementary Figure 10. nsEM of NX11$^{21Q.5}$-DNA$_{50}$ complexes. (a) Negatively stained electron micrographs. Scale bar: 50 nm. Representative micrograph from a single experiment. (b) Selected 2D class averages.

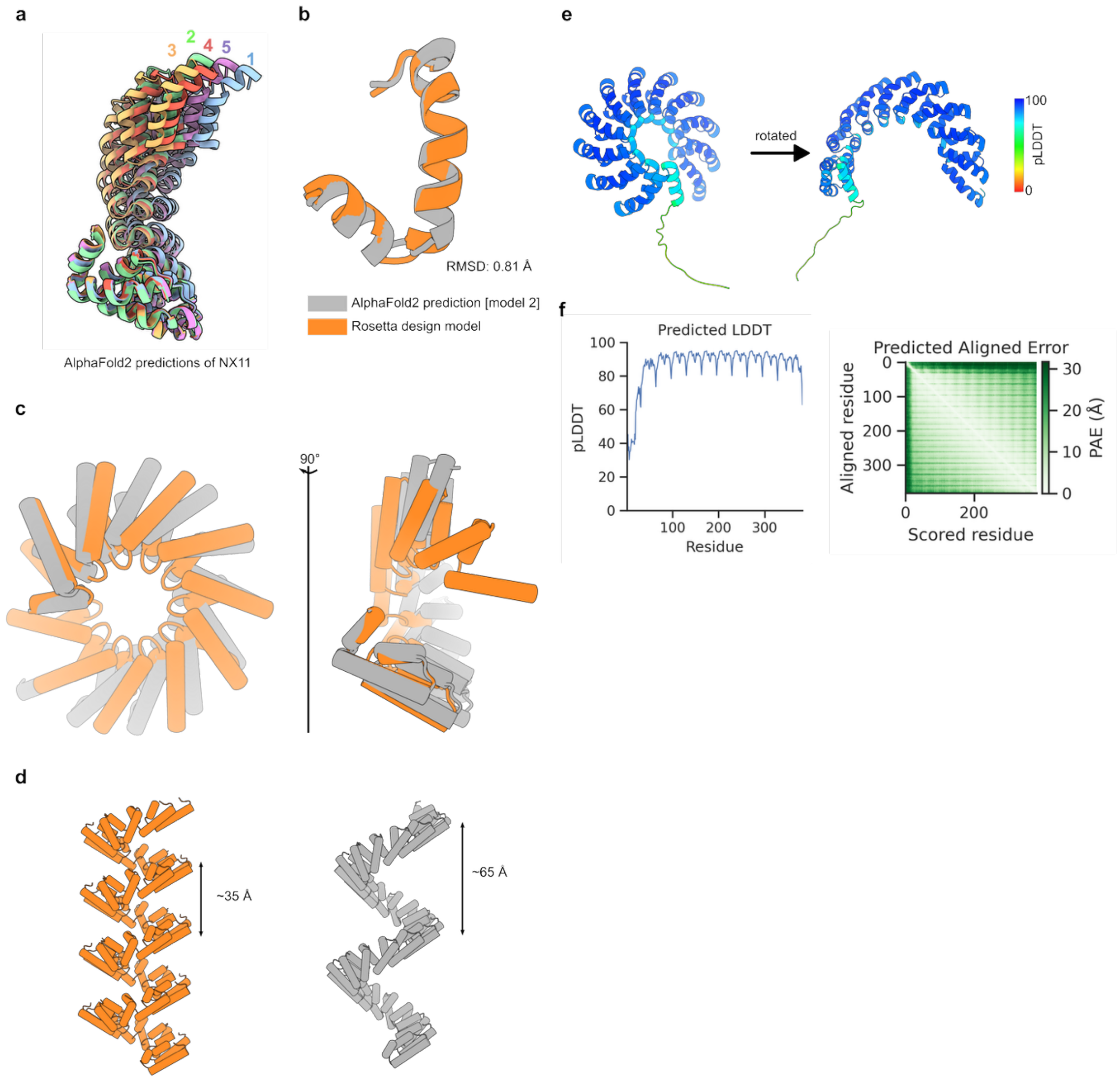

Supplementary Figure 11. Comparison of NX11 Rosetta design model and AlphaFold2 predictions.

(a) AlphaFold2 predictions of NX11 were generated using a local installation of AlphaFold2 (version 2.1) in single-sequence mode with all five parameter models. The input sequence is provided in **Supplementary Table 1.** The different AF2 models show some plasticity with respect to the apparent pitch of the NX11. **(b)** Alignment of a single repeat from from NX11, demonstrating that at the single repeat level the AF2 model 2 prediction agrees with the Rosetta model. **(c)** Alignment of the first repeat of the NX11 protein. **(d)** Repeat propagation using helical symmetry, where the symmetry parameters are inferred from two neighboring repeats in the design model or the AF2 prediction. Here the difference in helical rise in the predicted AF2 model 2 becomes apparent. The Rosetta design model follows the DNA helix rise of ~34 Å, but the AF2 model 2 predicts an extended rise of ~65 Å. (**e**) pLDDT scores colored on model 2 from two viewing angles. Colorbar shows pLDDT values. (**f**) pLDDT values vs residues and Predicted aligned error (PAE) plot for model 2.

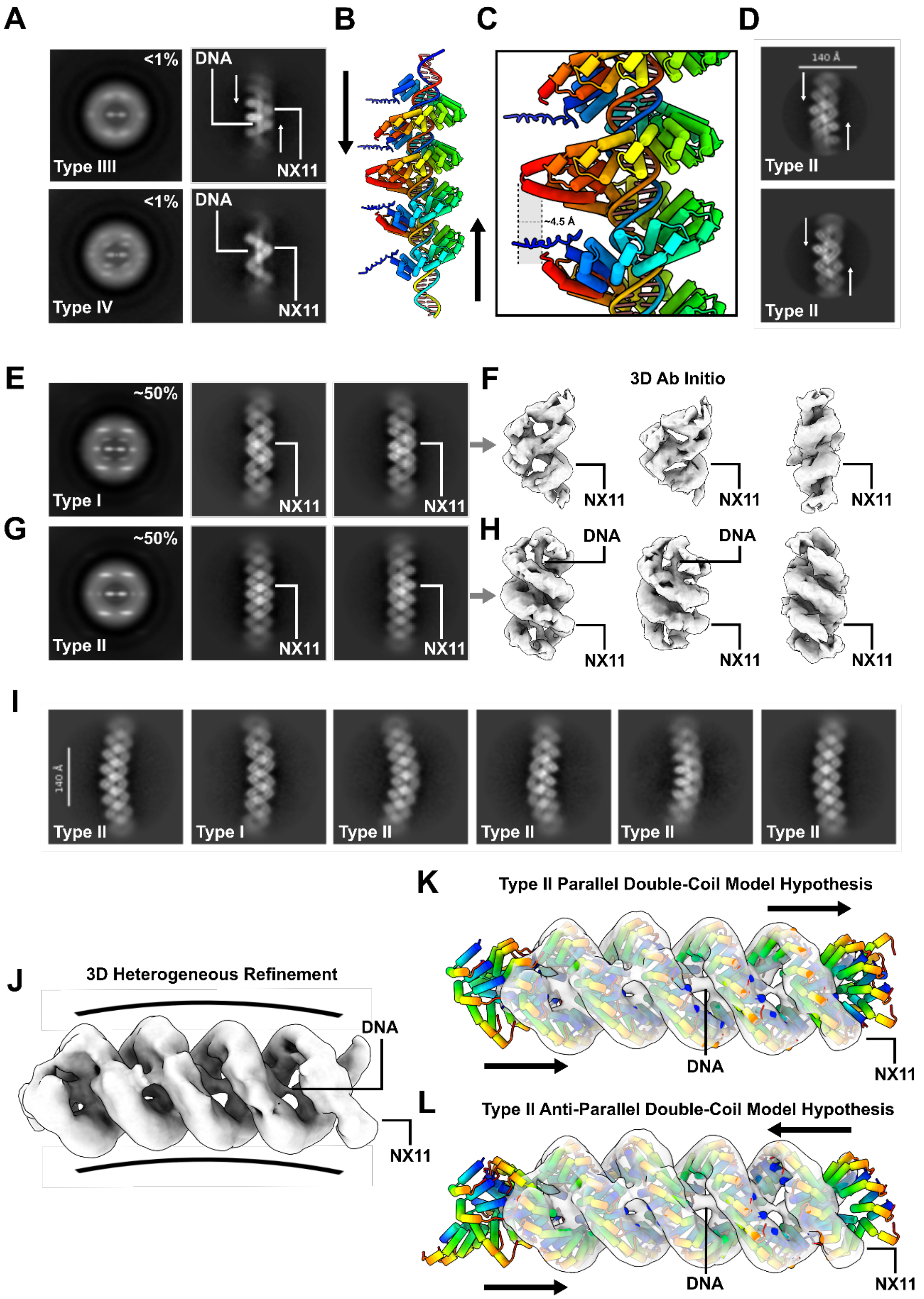

Supplementary Figure 12. Cryo-EM analysis of NX11–DNA assemblies. (A) Representative 2D class averages of two rare assembly types (<1% of total particles). *Top*: Type III assemblies, which display unique power spectra resembling the intended design model but exhibit "switching" of NX11 assembly directionality. *Bottom*: Type IV assemblies, which also show unique power spectra and appear related to Type III, but with a longer apparent helical pitch, suggestive of an intermediate assembly state prior to formation of a double-coil geometry. **(B)** AlphaFold3 prediction of NX11 in complex with the DNA template used in this study, revealing bi-directional assembly propagation along

the DNA. Assemblies terminate when colliding fibers of opposing directionality meet. **(C)** Zoomed-in view of the predicted termination point between colliding NX11 fibers, highlighting a ~4.5 Å difference in height of NX11 C-termini relative to adjacent fibers along the DNA major groove. **(D)** Select 2D class averages of Type II assemblies showing bi-directional assembly consistent with AlphaFold3 predictions, including subtle differences in fiber height. This may underlie the periodic height variation observed in AFM measurements of NX11–DNA assemblies. **(E)** Representative 2D class averages and power spectra of Type I assemblies, viewed along two principal axes, showing apparent helical pitches of 34 Å and 68 Å. **(F)** Ab initio 3D reconstruction of Type I assemblies, which appear flattened along one axis due to poor angular sampling; no DNA density is observed. **(G)** Additional 2D class averages and power spectra of Type I assemblies from multiple tilted views. **(H)** Ab initio 3D reconstruction of Type II assemblies, showing clear double-coil architecture with DNA density at the fiber core. **(I)** Evidence of flexibility in Type I and Type II assemblies, with continuous variation in fiber geometry. **(J)** Heterogeneous refinement performed in C1 symmetry using ab initio maps, yielding reconstructions consistent with Type II double-coil assemblies but also revealing bends along the fiber. (K–L) Schematic hypotheses for Type II assembly states. **(K)** *Parallel Double-Coil* model, in which both fibers propagate in the same direction. (L) *Anti-Parallel Double-Coil* model, in which opposing fibers propagate toward each other, terminating upon collision. Taken together, these data highlight the presence of multiple distinct assembly states (Type I, II, III, IV) with substantial structural heterogeneity. Type I and II assemblies account for ~50% of the total particles each, whereas Type III and IV represent <1%. Across all 2D class averages, there is a consistent absence of discernible α-helical secondary structure, suggesting that assembly occurs in a highly flexible and non-specific manner with pitches that deviate markedly from the design model.

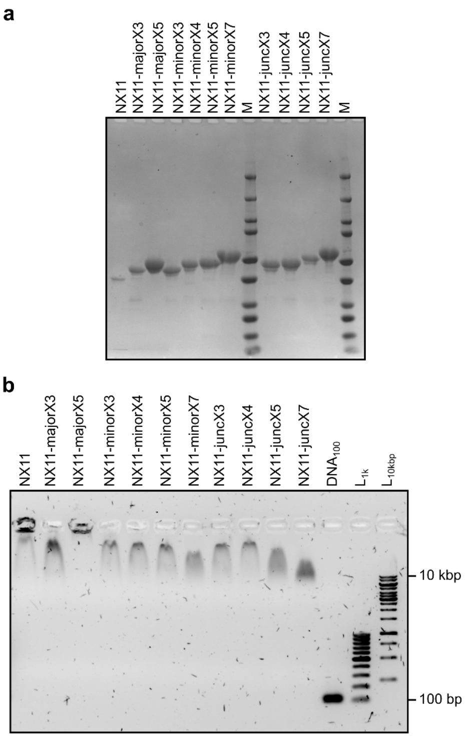

Supplementary Figure 13. Biochemical characterization of epitope-bearing NX11 variants.
(a) SDS-PAGE of NX11 variants displaying various malaria antigens (major, minor, or junctional) at different repeat numbers. NX11 carrying no antigens was loaded as a reference. The experiment was performed once. **(b)** EMSA of NX11 variants displaying malaria antigens assembled with DNA$_{100}$ (NoLimits, Thermo Fisher Scientific) at 1.1-fold excess. In all cases the DNA migration was slower due to assembly of protein. "L$_{1k}$" is a 100 bp gene ruler (ThermoFisher) and "L$_{10kbp}$" is a 1 kbp gene ruler (ThermoFisher) with maximal and minimal sizes indicated. The experiment was performed once.

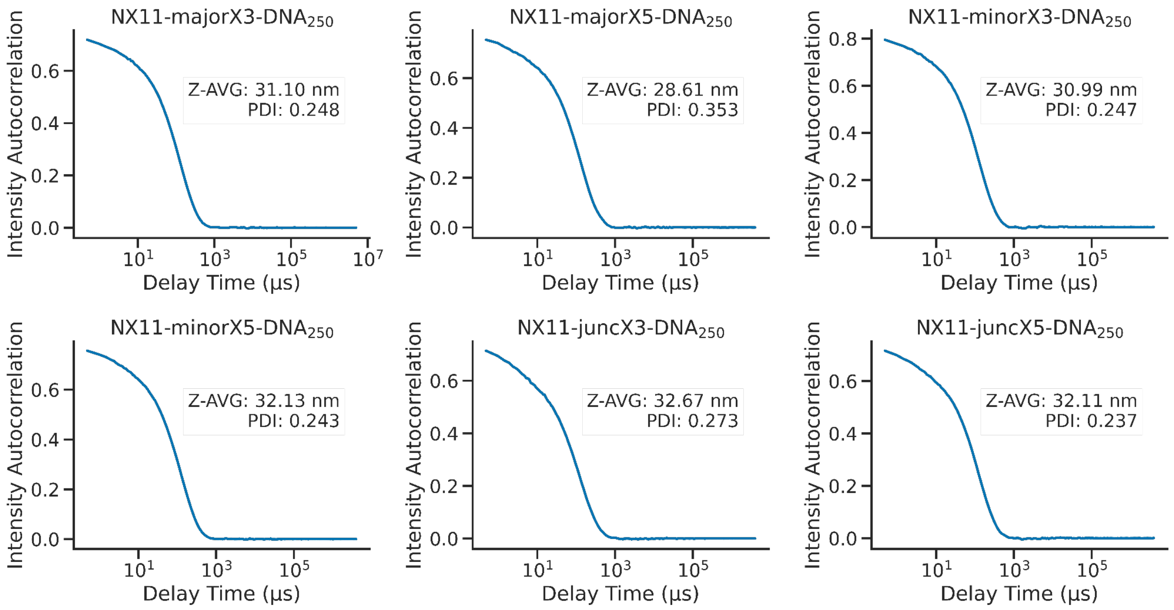

Supplementary Figure 14. Raw average autocorrelation vs. delay time for data presented in Fig. 4c. Z-AVG diameter and PDI are indicated in the plot.

a

b

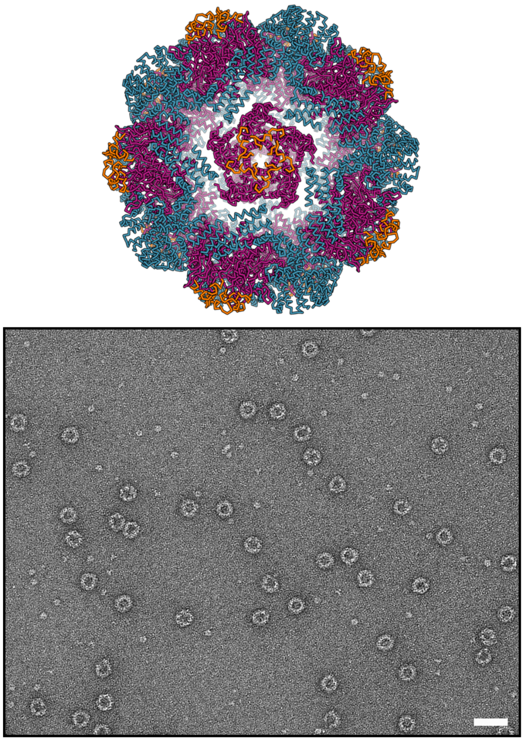

Supplementary Figure 15. Design model and electron micrographs of I53_dn5_majorX5.
(a) Design model of I53_dn5_majorX5 with I53_dn5B highlighted in blue, I53_dn5A highlighted in purple, and majorX5 insertion highlighted in orange. Each particle displays 60 copies of the majorX5 antigen. **(b)** Representative negative stain electron micrograph of *in vitro* assembled I53_dn5_majorX5. Scale bar: 50 nm. More than three images were acquired per condition with similar results.

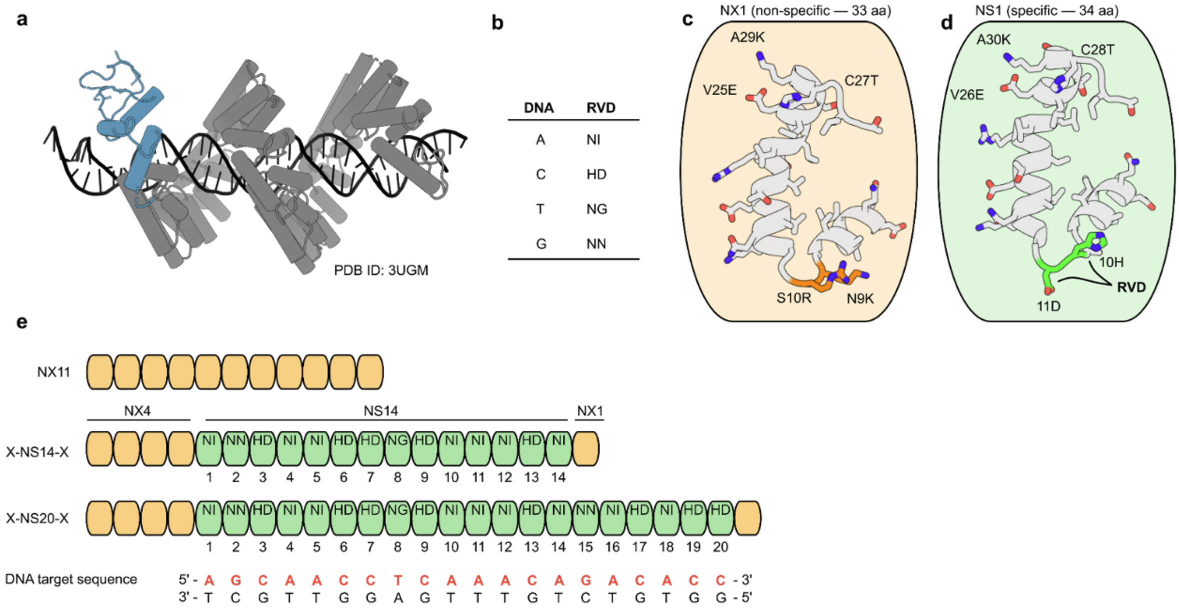

Supplementary Figure 16. **Design concept of Nucleo Specific (NS) proteins. (a)** CrystalTALE PthXo1 structure bound to its target (PDB ID: 3UGM). Blue: N-terminal region (NTR), and gray are the central repeat domains (CRD). **(b)** TALE residue di-variable (RVD) to DNA nucleotide specificity. **(c)** Previously designed DNA non-specific NX-repeat with mutations (S10R and N9K) that remove specific binding, and increase non-specific binding via electrostatics with DNA phosphate backbone. (**d**) NS-repeat, with its native residue divariable (RVD) site in green. Mutations A29K, V25E and C27T mutations from NX-repeat are conserved. But the RVD was re-inserted to bind to specific nucleotides A,C, T, G via different combinations of amino acids at this position. **(e)** Overview of designs generated in this study. In native TALEs the NTR is crucial to initiate DNA binding. We replaced the role of NTR by replacing it with NX4 to initiate non-specific binding to DNA. At the C-terminus a terminal NX1 was included so that potential oligomerization occurs through NX-NX interfaces previously confirmed by cryo-EM. RVD sites were chosen according to the table in panel **(b)**, two designs were generated: X-NS14-X and an extended X-NS20-X. The DNA target sequence is specific below, with red indicating the target nucleotides.

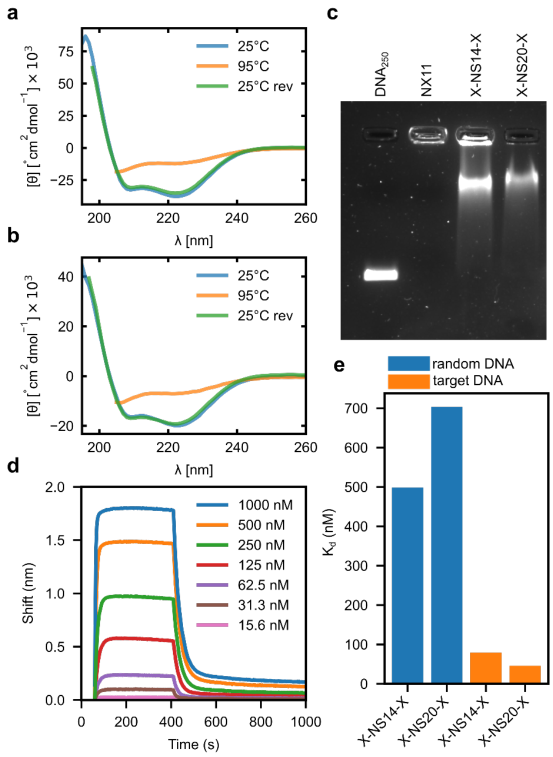

Supplementary Figure 17. Stability and binding specificity of NX4-NS14/20-NX1 proteins. (a) Circular dichroism of **(b)** NX4-NS14-NX1 and **(c)** NX4-NS20-NX1. Molar residue ellipticity [θ] is plotted over wavelength, showing a predominantly α-helical profile. Heating to 95 °C leads to a minimal change in ellipticity, and subsequent cooling to back to 20 °C (20°C rev) gives the same profile as the original at 20 °C, demonstrating that both proteins are thermostable > 95 °C. **(d)** Example biolayer interferometry (BLI) traces of biotinylated target DNA functionalized at the probe, and various concentrations of NX4-NS14-NX1 in solution. Association phase starts at ~50 sec, and dissociation at ~400 sec. Measurements are performed in TBS + 10 mM $MgCl_2$ + 0.5% BSA + 0.5% Tween-20. **(e)** Dissociation constant (K_d) from global fits of NX4-NS14-NX1 and NX4-NS20-NX1 for BLI experiments with target DNA and random DNA.

Column:	Superdex 200 Increase 10/300 GL
Sample:	1. Thyroglobulin (M_r 669 000), 3 mg/mL<br>2. Ferritin (M_r 440 000), 0.3 mg/mL<br>3. Aldolase (M_r 158 000), 3 mg/mL<br>4. Conalbumin (M_r 75 000), 3 mg/mL<br>5. Ovalbumin (M_r 44 000), 3 mg/mL<br>6. Carbonic anhydrase (M_r 29 000), 3 mg/mL<br>7. Ribonuclease A (M_r 13 700), 3 mg/mL
Sample volume:	500 µL
Buffer:	PBS (0.01 M phosphate buffer, 0.14 M NaCl, pH 7.4)
Flow rate:	0.5 mL/min
System:	ÄKTA explorer

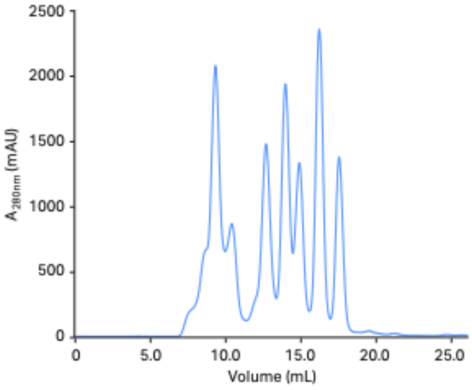

Supplementary Figure 18. Superdex S200 Increase 10/300 gl (Cytiva) column calibration data. From: *"SIZE EXCLUSION CHROMATOGRAPHY: Superdex 200 Increase columns."* Cytiva, MilliporeSigma, [no publication date], https://cdn.cytivalifesciences.com/api/public/content/digi-16542-original. Accessed 17 Aug. 2025.

Supplementary Table 1. Novel protein sequences generated in this study.

Design	Protein sequence
NX11	MGHHHHHHGSSENLYFQGSMPDQVVAIAKRGGKQALETVQRLLPELTQKHG<br>LTPDQVVAIAKRGGKQALETVQRLLPELTQKHGLTPDQVVAIAKRGGKQALET<br>VQRLLPELTQKHGLTPDQVVAIAKRGGKQALETVQRLLPELTQKHGLTPDQV<br>VAIAKRGGKQALETVQRLLPELTQKHGLTPDQVVAIAKRGGKQALETVQRLLP<br>ELTQKHGLTPDQVVAIAKRGGKQALETVQRLLPELTQKHGLTPDQVVAIAKRG<br>GKQALETVQRLLPELTQKHGLTPDQVVAIAKRGGKQALETVQRLLPELTQKH<br>GLTPDQVVAIAKRGGKQALETVQRLLPELTQKHGLTPDQVVAIAKRGGKQAL<br>ETVQRLLPELTQKHGW
NX5	MGHHHHHHGSSENLYFQGSMPDQVVAIAKRGGKQALETVQRLLPELTQKHG<br>LTPDQVVAIAKRGGKQALETVQRLLPELTQKHGLTPDQVVAIAKRGGKQALET<br>VQRLLPELTQKHGLTPDQVVAIAKRGGKQALETVQRLLPELTQKHGLTPDQV<br>VAIAKRGGKQALETVQRLLPELTQKHGW
NX11-1A	MGHHHHHHGSSENLYFQGSMPDQVAAIAKRGGKQALETVQRLLPELTQKHG<br>LTPDQVVAIAKRGGKQALETVQRLLPELTQKHGLTPDQVVAIAKRGGKQALET<br>VQRLLPELTQKHGLTPDQVVAIAKRGGKQALETVQRLLPELTQKHGLTPDQV<br>VAIAKRGGKQALETVQRLLPELTQKHGLTPDQVVAIAKRGGKQALETVQRLLP<br>ELTQKHGLTPDQVVAIAKRGGKQALETVQRLLPELTQKHGLTPDQVVAIAKRG<br>GKQALETVQRLLPELTQKHGLTPDQVVAIAKRGGKQALETVQRLLPELTQKH<br>GLTPDQVVAIAKRGGKQALETVQRLLPELTQKHGLTPDQVVAIAKRGGKQAL<br>ETVQRLLPELTQKHGW
NX11-3A	MGHHHHHHGSSENLYFQGSMPDQVAAIAKRGGKQAAETVQRLLPELTQKH<br>GLTPDQVVAIAKRGGKQALETVQRLLPELTQKHGLTPDQVVAIAKRGGKQAL<br>ETVQRLLPELTQKHGLTPDQVVAIAKRGGKQALETVQRLLPELTQKHGLTPD<br>QVVAIAKRGGKQALETVQRLLPELTQKHGLTPDQVVAIAKRGGKQALETVQR<br>LLPELTQKHGLTPDQVVAIAKRGGKQALETVQRLLPELTQKHGLTPDQVVAIA<br>KRGGKQALETVQRLLPELTQKHGLTPDQVVAIAKRGGKQALETVQRLLPELT<br>QKHGLTPDQVVAIAKRGGKQALETVQRLLPELTQKHGLTPDQVVAIAKRGGK<br>QALETVQRALPELTQKHGW
NX11-21.Q5	MGHHHHHHGSSENLYFQGSMPDQVVAIAKRGGKQALETVQRLLPELTQKHG<br>LTPDQVVAIAKRGGKQALETVQQLLPELTQKHGLTPDQVVAIAKRGGKQALET<br>VQRLLPELTQKHGLTPDQVVAIAKRGGKQALETVQQLLPELTQKHGLTPDQV<br>VAIAKRGGKQALETVQRLLPELTQKHGLTPDQVVAIAKRGGKQALETVQQLLP<br>ELTQKHGLTPDQVVAIAKRGGKQALETVQRLLPELTQKHGLTPDQVVAIAKRG<br>GKQALETVQQLLPELTQKHGLTPDQVVAIAKRGGKQALETVQRLLPELTQKH<br>GLTPDQVVAIAKRGGKQALETVQQLLPELTQKHGLTPDQVVAIAKRGGKQAL<br>ETVQRLLPELTQKHGW
NX11-majorX3	MGHHHHHHGSSENLYFQGSMPDQVVAIAKRGGKQALETVQRLLPELTQKHG<br>LTPDQVVAIAKRGGKQALETVQRLLPELTQKHGLTPDQVVAIAKRGGKQALET<br>VQRLLPELTQGNANPNANPNANPGDQVVAIAKRGGKQALETVQRLLPELTQK<br>HGLTPDQVVAIAKRGGKQALETVQRLLPELTQKHGLTPDQVVAIAKRGGKQA<br>LETVQRLLPELTQGNANPNANPNANPGDQVVAIAKRGGKQALETVQRLLPEL<br>TQKHGLTPDQVVAIAKRGGKQALETVQRLLPELTQKHGLTPDQVVAIAKRGG<br>KQALETVQRLLPELTQKHGLTPDQVVAIAKRGGKQALETVQRLLPELTQGNA<br>NPNANPNANPGDQVVAIAKRGGKQALETVQRLLPELTQKHGW
NX11-majorX5	MGHHHHHHGSSENLYFQGSMPDQVVAIAKRGGKQALETVQRLLPELTQKHG<br>LTPDQVVAIAKRGGKQALETVQRLLPELTQKHGLTPDQVVAIAKRGGKQALET<br>VQRLLPELTQGNANPNANPNANPNANPNANPGDQVVAIAKRGGKQALETVQ<br>RLLPELTQKHGLTPDQVVAIAKRGGKQALETVQRLLPELTQKHGLTPDQVVAI<br>AKRGGKQALETVQRLLPELTQGNANPNANPNANPNANPNANPGDQVVAIAK<br>RGGKQALETVQRLLPELTQKHGLTPDQVVAIAKRGGKQALETVQRLLPELTQ

	KHGLTPDQVVAIAKRGGKQALETVQRLLPELTQKHGLTPDQVVAIAKRGGKQ ALETVQRLLPELTQGNANPNANPNANPNANPNANPGDQVVAIAKRGGKQAL ETVQRLLPELTQKHGW
NX11-minorX3	MGHHHHHHGSSENLYFQGSMPDQVVAIAKRGGKQALETVQRLLPELTQKHG LTPDQVVAIAKRGGKQALETVQRLLPELTQKHGLTPDQVVAIAKRGGKQALET VQRLLPELTQGNPNVDPNANPNVGDQVVAIAKRGGKQALETVQRLLPELTQK HGLTPDQVVAIAKRGGKQALETVQRLLPELTQKHGLTPDQVVAIAKRGGKQA LETVQRLLPELTQGNPNVDPNANPNVGDQVVAIAKRGGKQALETVQRLLPEL TQKHGLTPDQVVAIAKRGGKQALETVQRLLPELTQKHGLTPDQVVAIAKRGG KQALETVQRLLPELTQKHGLTPDQVVAIAKRGGKQALETVQRLLPELTQGNP NVDPNANPNVGDQVVAIAKRGGKQALETVQRLLPELTQKHGW
NX11-minorX4	MGHHHHHHGSSENLYFQGSMPDQVVAIAKRGGKQALETVQRLLPELTQKHG LTPDQVVAIAKRGGKQALETVQRLLPELTQKHGLTPDQVVAIAKRGGKQALET VQRLLPELTQGNPNVDPNANPNVDPNAGDQVVAIAKRGGKQALETVQRLLP ELTQKHGLTPDQVVAIAKRGGKQALETVQRLLPELTQKHGLTPDQVVAIAKRG GKQALETVQRLLPELTQGNPNVDPNANPNVDPNAGDQVVAIAKRGGKQALE TVQRLLPELTQKHGLTPDQVVAIAKRGGKQALETVQRLLPELTQKHGLTPDQ VVAIAKRGGKQALETVQRLLPELTQKHGLTPDQVVAIAKRGGKQALETVQRLL PELTQGNPNVDPNANPNVDPNAGDQVVAIAKRGGKQALETVQRLLPELTQK HGW
NX11-minorX5	MGHHHHHHGSSENLYFQGSMPDQVVAIAKRGGKQALETVQRLLPELTQKHG LTPDQVVAIAKRGGKQALETVQRLLPELTQKHGLTPDQVVAIAKRGGKQALET VQRLLPELTQGNPNVDPNANPNVDPNANPNVGDQVVAIAKRGGKQALETVQ RLLPELTQKHGLTPDQVVAIAKRGGKQALETVQRLLPELTQKHGLTPDQVVAI AKRGGKQALETVQRLLPELTQGNPNVDPNANPNVDPNANPNVGDQVVAIAK RGGKQALETVQRLLPELTQKHGLTPDQVVAIAKRGGKQALETVQRLLPELTQ KHGLTPDQVVAIAKRGGKQALETVQRLLPELTQKHGLTPDQVVAIAKRGGKQ ALETVQRLLPELTQGNPNVDPNANPNVDPNANPNVGDQVVAIAKRGGKQAL ETVQRLLPELTQKHGW
NX11-minorX7	MGHHHHHHGSSENLYFQGSMPDQVVAIAKRGGKQALETVQRLLPELTQKHG LTPDQVVAIAKRGGKQALETVQRLLPELTQKHGLTPDQVVAIAKRGGKQALET VQRLLPELTQGNPNVDPNANPNVDPNANPNVDPNANPNVGDQVVAIAKRGG KQALETVQRLLPELTQKHGLTPDQVVAIAKRGGKQALETVQRLLPELTQKHGL TPDQVVAIAKRGGKQALETVQRLLPELTQGNPNVDPNANPNVDPNANPNVD PNANPNVGDQVVAIAKRGGKQALETVQRLLPELTQKHGLTPDQVVAIAKRGG KQALETVQRLLPELTQKHGLTPDQVVAIAKRGGKQALETVQRLLPELTQKHGL TPDQVVAIAKRGGKQALETVQRLLPELTQGNPNVDPNANPNVDPNANPNVD PNANPNVGDQVVAIAKRGGKQALETVQRLLPELTQKHGW
NX11-juncX3	MGHHHHHHGSSENLYFQGSMPDQVVAIAKRGGKQALETVQRLLPELTQKHG LTPDQVVAIAKRGGKQALETVQRLLPELTQKHGLTPDQVVAIAKRGGKQALET VQRLLPELTQGNPDPNANPNVDPNAGDQVVAIAKRGGKQALETVQRLLPELT QKHGLTPDQVVAIAKRGGKQALETVQRLLPELTQKHGLTPDQVVAIAKRGGK QALETVQRLLPELTQGNPDPNANPNVDPNAGDQVVAIAKRGGKQALETVQR LLPELTQKHGLTPDQVVAIAKRGGKQALETVQRLLPELTQKHGLTPDQVVAIA KRGGKQALETVQRLLPELTQKHGLTPDQVVAIAKRGGKQALETVQRLLPELT QGNPDPNANPNVDPNAGDQVVAIAKRGGKQALETVQRLLPELTQKHGW
NX11-juncX4	MGHHHHHHGSSENLYFQGSMPDQVVAIAKRGGKQALETVQRLLPELTQKHG LTPDQVVAIAKRGGKQALETVQRLLPELTQKHGLTPDQVVAIAKRGGKQALET VQRLLPELTQGNPDPNANPNVDPNANPNVGDQVVAIAKRGGKQALETVQRL LPELTQKHGLTPDQVVAIAKRGGKQALETVQRLLPELTQKHGLTPDQVVAIAK RGGKQALETVQRLLPELTQGNPDPNANPNVDPNANPNVGDQVVAIAKRGGK QALETVQRLLPELTQKHGLTPDQVVAIAKRGGKQALETVQRLLPELTQKHGLT PDQVVAIAKRGGKQALETVQRLLPELTQKHGLTPDQVVAIAKRGGKQALETV

	QRLLPELTQGNPDPNANPNVDPNANPNVGDQVVAIAKRGGKQALETVQRLL<br>PELTQKHGW
NX11-juncX5	MGHHHHHHGSSENLYFQGSMPDQVVAIAKRGGKQALETVQRLLPELTQKHG<br>LTPDQVVAIAKRGGKQALETVQRLLPELTQKHGLTPDQVVAIAKRGGKQALET<br>VQRLLPELTQGNPDPNANPNVDPNANPNVDPNAGDQVVAIAKRGGKQALET<br>VQRLLPELTQKHGLTPDQVVAIAKRGGKQALETVQRLLPELTQKHGLTPDQV<br>VAIAKRGGKQALETVQRLLPELTQGNPDPNANPNVDPNANPNVDPNAGDQV<br>VAIAKRGGKQALETVQRLLPELTQKHGLTPDQVVAIAKRGGKQALETVQRLLP<br>ELTQKHGLTPDQVVAIAKRGGKQALETVQRLLPELTQKHGLTPDQVVAIAKRG<br>GKQALETVQRLLPELTQGNPDPNANPNVDPNANPNVDPNAGDQVVAIAKRG<br>GKQALETVQRLLPELTQKHGW
NX11-juncX7	MGHHHHHHGSSENLYFQGSMPDQVVAIAKRGGKQALETVQRLLPELTQKHG<br>LTPDQVVAIAKRGGKQALETVQRLLPELTQKHGLTPDQVVAIAKRGGKQALET<br>VQRLLPELTQGNPDPNANPNVDPNANPNVDPNANPNVDPNAGDQVVAIAKR<br>GGKQALETVQRLLPELTQKHGLTPDQVVAIAKRGGKQALETVQRLLPELTQK<br>HGLTPDQVVAIAKRGGKQALETVQRLLPELTQGNPDPNANPNVDPNANPNV<br>DPNANPNVDPNAGDQVVAIAKRGGKQALETVQRLLPELTQKHGLTPDQVVAI<br>AKRGGKQALETVQRLLPELTQKHGLTPDQVVAIAKRGGKQALETVQRLLPEL<br>TQKHGLTPDQVVAIAKRGGKQALETVQRLLPELTQGNPDPNANPNVDPNAN<br>PNVDPNANPNVDPNAGDQVVAIAKRGGKQALETVQRLLPELTQKHGW
X-NS14-X	MGHHHHHHGSSENLYFQGSMPDQVVAIAKRGGKQALETVQRLLPELTQKHG<br>LTPDQVVAIAKRGGKQALETVQRLLPELTQKHGLTPDQVVAIAKRGGKQALET<br>VQRLLPELTQKHGLTPDQVVAIAKRGGKQALETVQRLLPELTQKHGLTPDQV<br>VAIASNIGGKQALETVQRLLPELTQKHGLTPDQVVAIASNNGGKQALETVQRL<br>LPELTQKHGLTPDQVVAIASHDGGKQALETVQRLLPELTQKHGLTPDQVVAIA<br>SNIGGKQALETVQRLLPELTQKHGLTPDQVVAIASNIGGKQALETVQRLLPELT<br>QKHGLTPDQVVAIASHDGGKQALETVQRLLPELTQKHGLTPDQVVAIASHDG<br>GKQALETVQRLLPELTQKHGLTPDQVVAIASNGGGKQALETVQRLLPELTQK<br>HGLTPDQVVAIASHDGGKQALETVQRLLPELTQKHGLTPDQVVAIASNIGGKQ<br>ALETVQRLLPELTQKHGLTPDQVVAIASNIGGKQALETVQRLLPELTQKHGLT<br>PDQVVAIASNIGGKQALETVQRLLPELTQKHGLTPDQVVAIASHDGGKQALET<br>VQRLLPELTQKHGLTPDQVVAIASNIGGKQALETVQRLLPELTQKHGLTPDQV<br>VAIAKRGGKQALETVQRLLPELTQKHGW
X-NS20-X	MGHHHHHHGSSENLYFQGSMPDQVVAIAKRGGKQALETVQRLLPELTQKHG<br>LTPDQVVAIAKRGGKQALETVQRLLPELTQKHGLTPDQVVAIAKRGGKQALET<br>VQRLLPELTQKHGLTPDQVVAIAKRGGKQALETVQRLLPELTQKHGLTPDQV<br>VAIASNIGGKQALETVQRLLPELTQKHGLTPDQVVAIASNNGGKQALETVQRL<br>LPELTQKHGLTPDQVVAIASHDGGKQALETVQRLLPELTQKHGLTPDQVVAIA<br>SNIGGKQALETVQRLLPELTQKHGLTPDQVVAIASNIGGKQALETVQRLLPELT<br>QKHGLTPDQVVAIASHDGGKQALETVQRLLPELTQKHGLTPDQVVAIASHDG<br>GKQALETVQRLLPELTQKHGLTPDQVVAIASNGGGKQALETVQRLLPELTQK<br>HGLTPDQVVAIASHDGGKQALETVQRLLPELTQKHGLTPDQVVAIASNIGGKQ<br>ALETVQRLLPELTQKHGLTPDQVVAIASNIGGKQALETVQRLLPELTQKHGLT<br>PDQVVAIASNIGGKQALETVQRLLPELTQKHGLTPDQVVAIASHDGGKQALET<br>VQRLLPELTQKHGLTPDQVVAIASNIGGKQALETVQRLLPELTQKHGLTPDQV<br>VAIASNNGGKQALETVQRLLPELTQKHGLTPDQVVAIASNIGGKQALETVQRL<br>LPELTQKHGLTPDQVVAIASHDGGKQALETVQRLLPELTQKHGLTPDQVVAIA<br>SNIGGKQALETVQRLLPELTQKHGLTPDQVVAIASHDGGKQALETVQRLLPEL<br>TQKHGLTPDQVVAIASHDGGKQALETVQRLLPELTQKHGLTPDQVVAIAKRG<br>GKQALETVQRLLPELTQKHGW

Supplementary Table 2. PfCSP epitopes used in the mice study.

Immunogen	Epitope sequence
majorX3	NANPNANPNANP
majorX5	NANPNANPNANPNANPNANP
minorX3	NPNVDPNANPNV
minorX5	NPNVDPNANPNVDPNANPNV
juncX3	NPDPNANPNVDPNA
juncX5	NPDPNANPNVDPNANPNVDPNA

Supplementary Table 3. DNA sequences used in bio-layer interferometry experiments. Bold indicates expected binding of proteins. With the NS-repeat targeting sequence highlighted in red. Biotin was functionalized on one of the oligo's at the 5' including a 6 basepair spacer (AAAAAA) and a two bp spacer (GA) at the 3'.

Design	DNA	DNA duplex sequence
X-NS14-X	Target	5′-biotin-AAAAAA**ATCTAGCAACCTCAAACAG**GA-3′<br>3′- TTTTTTTAGATCGTTGGAGTTTGTCCT-5′
	Random	5′-biotin-AAAAAAATCAGACCGACATCTAATCGA-3′<br>3′- TTTTTTTAGTCTGGCTGTAGATTAGCT-5′
X-NS20-X	Target	5′-biotin-AAAAAA**ATCTAGCAACCTCAAACAGACACCA**GA-3′<br>3′- TTTTTTTAGATCGTTGGAGTTTGTCTGTGGTCT-5′
	Random	5′-biotin-AAAAAAATCAGACCGACATCTAATCCGCAACGA-3′<br>3′- TTTTTTTAGTCTGGCTGTAGATTAGGCGTTGCT-5′
