# Supplementary material for: Redesign of TALE proteins for DNA-templated assembly of protein fibers
Source: Nat Commun. 2026 May 19;17:6582. doi: 10.1038/s41467-026-73313-8 (PMC13381548; doi:10.1038/s41467-026-73313-8)
Supplement: Supplementary file 2 — Transparent Peer Review file [file 41467_2026_73313_MOESM2_ESM.pdf]

nature portfolio

Peer Review File

Redesign of TALE proteins for DNA-templated assembly of protein fibers

Corresponding Author: Professor Neil King

This file contains all reviewer reports in order by version, followed by all author rebuttals in order by version.

Version 0:

Reviewer comments:

Reviewer #1

(Remarks to the Author)
Based on a fragment derived from a DNA-binding protein, the authors designed a filamentous complex of DNA-protein using DNA as a template, characterized the complex by biochemical and structural approaches, and explored its application as an antigen display platform. Using DNA as a template to control assembly of protein molecules is an interesting attempt in the field of macromolecular materials, but from the experimental point of view, there are still some problems to be solved in this paper.

Major problems:
1) The article emphasizes several times that the cooperative binding of NX-proteins on DNA strands, but from Figure 2e, the binding is not cooperative. For cooperative binding, we would expect that after one DNA250 is bound by one NX11, more NX11 binds to that DNA250 more readily, and NX11 does not bind to another DNA molecule until all sites on this DNA strand are occupied by NX11. The cooperative binding (protein-protein interactions during DNA binding) may indeed improve the efficiency of interactions between protein and DNA; however, experimental evidence is needed to verify this in the manuscript.
2) About AFM analysis, the author mentioned "confirmed the presence of a major periodicity of 40 ± 10 nm and additionally revealed a smaller periodicity of 7 ± 2 nm (Fig. 3g,h and Fig. S5)". Is the "a periodicity" referred to here being “pitch” (Y-axis coordinates in Figure 3h)? Does “pitch” here mean the same thing as helical pitch (Figure 3j, 34 Å or 68 Å) for cryo-EM? Why is there such a big difference? How many NX11s does "a periodicity" correspond to?
3) About cryo-EM analysis, although the authors have collected a considerable number of electron microscopy movies, the structural analysis has not given a definitive result. The current data do not support "a protein filament helically wraps around the dsDNA using a binding mode similar to that of natural TALE proteins" (abstract, lines 30-32). It is recommended to show the "double-coiled assembly" in detail, which is significantly different from the previous crystal structure (Figure 1a). In fact, in the case of Type II assemblies, it may also be "double-coiled assembly" (a two-start helix). As mentioned by authors, there are "150 2D class averages" (Line 456). What percentage of segments does the 2D classification shown in Figure 3j represent? I suggest authors improve the quality of the cryo-EM data and do a 3D reconstruction (or local reconstruction) with sufficient resolution to elucidate the relationship between the protein helix and the DNA helix. In addition, “pitch” is usually applied only for one helix, not directly between two helixes (there is confusion about the use of “pitch” in Figure 3j).

Minor issues:
Figure 1a: Where is the CTR domain on the illustration?
Lines 128-129: To support “eluted as a monomer from size-exclusion chromatography (SEC)”, please use SEC protein standard for comparison.
Line 257: It is also preferable to use a platform-free antigen for direct comparison.

Reviewer #2

(Remarks to the Author)
The study by de Haas et al. reports on the design, production and characterization of sequence-independent DNA-templated proteins for controllable antigen display. The authors first (re)designed a sequence-independent DNA-binding repeat protein by altering the sequence-specific DNA-binding domain of a TALE protein. Then, the assembly of monomers was templated

using dsDNA and ultimately, after insertion of epitopes between the repeats, the applicability of the complexes for antigen display was studied.

The linear dsDNA templated assembly of proteins into tubular nanostructures is particularly common in filamentous viruses. In vitro, similar structures have been previously shown with native capsid proteins (e.g. doi.org/10.1021/ja056656f). Furthermore, linear helical protein structures of well-defined lengths have been assembled by using scaffolded DNA nanostructures (e.g. doi.org/10.1038/s41565-023-01443-x). The novelty of the current manuscript is therefore claimed to be in the use of de novo proteins to achieve the same (Page 2, line 45). However, the approach of de Haas et al. does not rely on fully de novo designed proteins, but rather engineered TALE proteins, which show less sequence specific DNA binding. These together reduce the conceptual novelty of the manuscript.

While de Haas et al. show the versatility of the templating effect by using DNA with different lengths, the choice of the particular length (48, 50, 250, 750 or 1k bp) used is not reasoned, nor if the length potentially impacts the quality of the assembly and antigen properties. Following their assumption of 1 NX per bp, none of the chosen templates matches exactly with NX11, which makes one wonder about the protein coverage especially at the end of the dsDNA template. Additionally, a possible mismatch between the helical twist of NX11 and the dsDNA is proposed. How does the frequency of such mismatch occurrence depend on the length of the template DNA? This mismatch could be one of the reasons preventing 3D reconstruction.

By introducing mutations on the protein-protein interface, the authors demonstrated its importance for the propagation of the assembly. The assembly was proposed to consist of two phases, rapid nucleation and reorganization. Since the assembly is sequence-independent, what is the driving force of the nucleation? Comparing the excess screening gels of NX11 and NX5 for excesses below 3× showed smear for NX11 but rather discrete bands for NX5. Why do the two repeat proteins behave differently and how does it influence the assembly? Moreover, do the proteins have a directionality which would contribute to the observed mismatches? A more insightful discussion on the assembly would be beneficial for the manuscript.

The comparison of Type I and Type II assemblies is somewhat confusing, and no actual reason is given. The authors simply indicate that Rosetta and AlphaFold2 disagree in their predictions in a way that happens to match the experimental finding, but it does not explain the underlaying reason why two different types are observed.

The controls are poorly designed in the immunogenicity studies. For example, NX11 alone, JuncX3/5 alone, DNA alone, plain NX11-DNA250 complex, a commercially established positive control and a negative control must be added alongside the studied samples. Furthermore, the authors do not present any attempts to characterize the in vivo stability of the NX-DNA complexes. Establishing the structural integrity of the studied complexes is crucial for the validating the results that organizing the NXs matters.

Below are listed some more technical points for further improving the manuscript.

1. DLS data is not properly presented. Why is the y-axis Amplitude %? This should be volume distribution or preferably scattering intensity (intensity distribution). All measure distributions in Figures 2 and 4 should be accompanied by Z-average size and PDI values as well as the measured autocorrelations.
2. If assemblies templated by DNA with lengths shorter than 50 bp are susceptible to side-by-side dimerization, how conclusive is the DLS data obtained for NX11-DNA48? Can the authors elaborate on why this phenomenon was only observed for short DNA templates, since the "weak yet repetitive interactions along the DNA fibre" would expectedly be also present in longer DNA-protein hybrids.
3. Fig. 2f: How long is Benzonase active at the tested conditions, i.e., how informative is for instance the ON incubation?
4. The data in Figure 3c shows very few data points and none above 2 nm. Yet, judging from the AFM images some of the showed structures are higher. In panels e/f the height profile should also include the bare substrate at the beginning and end of the profile to allow comparison to panel c. Now the data fluctuates between +/- 0.5 nm, which is close to the observed difference between DNA and NX11-DNA samples. Could the authors comment on this?
5. Page 6, line 246-249: "...DNA250 into complexes at a 1.1× excess, and the assembly was confirmed by EMSA (Fig. S9)". Figure S9b in contrast refers to "...assembled with DNA100 at 3-fold excess." Please clarify the discrepancy.
6. The analysis of NX1121Q.5-DNA48 complexes shows two main particle populations. With which frequency does either of them occur, is there a preference for one of the populations? How do the authors imagine the formation of two NX11 fibrils wrapping the DNA and what causes it?
7. Figure 4 shows multiple nsTEM images, which are not fully convincing. The quality seems lower than with AFM imaging. Why was AFM or cryo-EM not utilized for these samples?
8. The assembly experiments and complex characterizations have been performed with 3× excess. It did not get entirely clear to me, why the excess was decreased to 1.1× for the antigen display studies. Following the argumentation given for NX11-1A/3A and looking at Fig. 2e/S3, NX11 appears with a slight smear at 1× excess which might suggest the presence of intermediate assembly species. How do complexes with 1.1× excess compare to complexes with 3× excess?
9. Along the line, does the insertion of the epitopes into the loop region interfere with the assembly, for instance with the coverage of the protein on the DNA template or the coiling parameters?
10. Figure 4d, were the injected samples purified from excess/unbound protein?
11. Nucleic acid delivery is mentioned as a potential application. How strongly are the proteins bound to the DNA and how do the authors imagine the disassembly of the complexes/release of the DNA?

Reviewer #3

(Remarks to the Author)
I co-reviewed this manuscript with one of the reviewers who provided the listed reports. This is part of the Nature Communications initiative to facilitate training in peer review and to provide appropriate recognition for Early Career Researchers who co-review manuscripts.

Reviewer #4

(Remarks to the Author)
The manuscript titled “Computational redesign of TALE proteins for DNA-templated assembly of protein fibers” describes the creation and characterization of engineered TALE proteins. The authors thoroughly characterize the proteins as well as their assembly on DNA. Convincing evidence is provided that the proteins polymerize along the DNA in a well-ordered manner. Furthermore, the authors demonstrated promising use of the DNA-protein complex for vaccine applications.
Generally, the work is relevant and well presented and experimental evidence is convincing, however, some points could be improved regarding the atomic force microscopy analysis.

(1) Page 5, lines 181-184: The discussion regarding the measured height of the DNA and DNA-protein complex deviating from the expected height is too shallow and possibly inaccurate. The authors claim that for samples (“cylindrical objects”) with a height less than 4 nm “non-linear underestimation” occurs, referencing Ref. 24. However, there is no such claim or proof made in that reference. Ref. 24 shows among other things several possible influences to the measured height in AFM, none of which are mentioned in the manuscript or allow for the assumption that every object with height of 4 nm is underestimated. Most likely the two main causes for the height discrepancy are (a) the measurement in air and (b) too high tapping forces. (a) It was previously shown that measurement in air could cause an apparent lower height of DNA compared to measurement in liquid [F. Moreno-Herrero et al. Ultramicroscopy 96(2003)167–174]. (b) If the sample is not rigid, even in tapping mode, the force can significantly deform the sample, reducing the height. To get accurate height measurements of biological molecules with AFM, very soft cantilevers (spring constant ~0.1 N/m) are often used [T. Ando et al., PNAS, 98 (22) 12468-12472]. The authors used cantilevers with 5 N/m spring constant. It is not unreasonable to assume that this also could cause significant deformation of the DNA and protein complexes.

(2) Point (1) does not per se invalidate the authors findings, but it is possible that significant deformation during imaging could negatively influence the ability to detect such small features (e.g. 7 nm periodicities). Electron microscopy results seem convincing and fit well with the AFM results, but some additional analysis could help to strengthen the results. (a) Performing the FFT analysis on DNA without NX11 proteins and (b) performing the FFT analysis along random paths on the mica substrate between the DNA (without proteins). From Fig. 3a, it seems that the bare DNA is also not of uniform height and it would be good to confirm that the periodicity is not something that comes from these corrugations. Furthermore, the mica substrate might also have some height fluctuations that could propagate and cause apparent height differences in the protein complexes. It is not too clearly visible, but there seem to be some corrugations of the mica, although the resolution of the image is not good enough to be sure.

(3) Page 11, Line 413: “cleaved the mica surface by etching” I am not familiar with this procedure of cleaving by etching. It might be a good idea to provide a reference. Typically, mica is cleaved by exfoliating the top layers by use of adhesive tape.

(4) Page 11, Line 421-424: The authors describe that the samples were imaged in a regime of “constant phase change”. Do the authors mean that the phase shift was constant? Meaning the imaging was performed by keeping the phase shift at a set value? I.e. phase modulation instead of the more common amplitude modulation? If so, why? Moreover, it would be good to provide the cantilever’s oscillation amplitude (in nm, if possible) and the amplitude set-point (which can be used to estimate tapping forces). Additionally, since cantilevers actual spring constants can deviate strongly from nominal values, it might be a good idea to actually calibrate the cantilever (e.g. Thermal Sweep or Sader’s method) and provide results in method section.

(5) The authors use the term “high-resolution” AFM to describe their measurements, but what is “high-resolution” AFM exactly? While I do agree that the resolution of the AFM images is adequate for the authors’ samples, it is not particularly high resolution. Generally, AFM can achieve atomic resolution (in specific conditions) so 2 nm/pixel is not that high. I would suggest to remove the “high-resolution” modifier or justify its use appropriately.

Minor points:

- Ref. 32 is the same as Ref 27
- Fig. S5 has very low resolution and/or small text. It is impossible to read the legends in (d) and, moreover, there seem to be three lines/curves according to the legends, but it is difficult to see that clearly and identify what the curves represent.
- Lines 348-352 have a different font and line spacing compared to the rest of the text.

Reviewer #5

(Remarks to the Author)

This is very interesting and innovative study that seeks to de novo engineer DNA-templated protein fibers utilizing TALE proteins as a template. Overall there is high degree of novelty, and the application of such proteins for potential uses is clear. The data adequately supports the conclusion and the manuscript is well written and very easy to understand. I have just two editorial suggestions prior to publication:
1. The sequence of the DNA is not discussed in the results or discussion. Does this matter? Are there some sequences that work better than others? Also, to what extent would it be possible to engineer DNA sequence specificity into the TALE fibril?
2. I am assuming that "peptide alone" would have induced no or very poor antibody response in Figure 4D? Please elaborate on this.

Version 1:

Reviewer comments:

Reviewer #1

(Remarks to the Author)
The authors have adequately addressed the issues I raised. I support the publication of the revised paper.

Reviewer #2

(Remarks to the Author)
The authors have carried out revisions and answered some of the comments satisfactorily. However, in many cases the text and experimental revisions are not sufficient to clarify the original issues. Carrying out the following revisions would aid to validate the manuscript claims.

1. Discussion on the assembly mechanisms and defects remains unclear. Revised discussion on introducing local defects is taken out of context and does, at the point where it is mentioned, in no instance refer to problems that a possible mismatch in the helical twist of NX11 and the DNA cause. Instead, speculations about the mismatch are raised when talking about the different periodicities observed in AFM. As the response of 7 +/- 2 nm corresponding most likely to a single NX11 molecule given to Reviewer 1, this would be important to mention in the text.
2. The assembly process itself is suggested to be cooperative and driven by nucleation followed by elongation. How does the added experiment on assembling a fusion protein of NX11-sfGFP give information on the assembly process, especially if conducted at excesses that are too low for a full coating (at least judging from the EMSA gel given in Fig. S24 for NX11)? Also, wouldn't the fusion protein possibly interfere with the assembly process?
3. The authors mention that "NX11-NX11 interactions may slow down the diffusion and this is a nucleation event for elongation of NX11 on the DNA fiber". Does this mean that there first exists protein-protein interaction in solution, followed by nucleation on the DNA substrate? If there are already NX11-NX11 interactions, does this mean that more than one protein could be expected to move along the DNA filament as a unit before nucleation happens? In that case, what's the critical number of proteins for nucleation?
4. How representative are the DLS results for nucleation, if DNA48 was used for the experiments, which can have up to 4 NX11 units assembled?
5. Regarding the immunogenicity studies, the authors assume that NX11 alone, DNA alone and NX11-DNA complexes do not elicit an immune response, and added I53.dn5_majorX5 as positive control. These controls are still not sufficient to back up the claim that organizing the antigens makes a difference. Each of the NX-epitope variants must be tested for their respective response alone, especially since the complexes have not been purified from excess proteins (the authors state that these could contribute to immunogenicity: the reason why the excess was reduced to 1.1x in the antigen display studies).
6. If in vivo stability is too difficult to study, the authors should at least investigate the stability of the complexes in simulated conditions (presence of serum, nucleases, competing DNA binding agents etc.).

Reviewer #3

(Remarks to the Author)
I co-reviewed this manuscript with one of the reviewers who provided the listed reports. This is part of the Nature Communications initiative to facilitate training in peer review and to provide appropriate recognition for Early Career Researchers who co-review manuscripts.

Reviewer #4

(Remarks to the Author)
The manuscript "Computational redesign of TALE proteins for DNA-templated assembly of protein fibers" by Robbert J. de Haas et al. was thoroughly revised. I am fully convinced that the AFM images are devoid of artifacts that could influence the analysis and that the measured periodic fluctuations are indeed caused by NX11 proteins.
I found no further issues and fully support the publication of the manuscript.

Reviewer #5

(Remarks to the Author)

The authors have responded productively to reviewer concerns. I have no further suggestions.

Version 2:

Reviewer comments:

Reviewer #2

(Remarks to the Author)
The authors have responded to my comments satisfactorily.

Open Access This Peer Review File is licensed under a Creative Commons Attribution 4.0 International License, which permits use, sharing, adaptation, distribution and reproduction in any medium or format, as long as you give appropriate credit to the original author(s) and the source, provide a link to the Creative Commons license, and indicate if changes were made.
In cases where reviewers are anonymous, credit should be given to 'Anonymous Referee' and the source.
The images or other third party material in this Peer Review File are included in the article's Creative Commons license, unless indicated otherwise in a credit line to the material. If material is not included in the article's Creative Commons license and your intended use is not permitted by statutory regulation or exceeds the permitted use, you will need to obtain permission directly from the copyright holder.
To view a copy of this license, visit https://creativecommons.org/licenses/by/4.0/

Comments from Reviewer 1:

> **Reviewer 1:** The cooperative binding (protein-protein interactions during DNA binding) may indeed improve the efficiency of interactions between protein and DNA; however, experimental evidence is needed to verify this in the manuscript.

We appreciate the reviewer's comment regarding the need for experimental evidence of cooperative binding. While we observed several indicators suggestive of protein-protein interactions during DNA binding such as high benzonase stability and electron microscopy showing NX11 proteins tightly positioned along DNA, we agree with the reviewer that these experiments do not directly prove cooperativity. In response we conducted electron microscopy using a GFP fusion NX11 construct, assembled with 250 bp DNA at 0.5X molar excess to the DNA. Micrographs revealed localized protein clusters along the DNA, but no continuous coating (**Fig. S2**). This supports the point that while NX11-DNA interactions occur, they do not result in strong cooperativity, as there are no fully formed fibers visible.

Changes made:

- We have revised the main text and removed any overstatement with regards to presumed cooperative behavior.
- Added paragraph to Results (lines 178): "*We also conducted electron microscopy with a N-terminal fused superfolder GFP (sfGFP) to NX11 assembled with* DNA_{250} *at 0.5X molar excess. The resulting micrographs revealed localized protein clusters along the DNA, but showed no consistent coating, suggesting that while NX11-NX11 interfaces likely form, these do not result in strong cooperative assembly (**Fig. S2**). Together, these data show that the energy gain from the NX11-NX11 interface is crucial but not sufficient to drive a fully cooperative assembly.*"
- Added **new Fig. S2** showing electron micrograph of sfGFP-NX11 at 0.5X molar excess.

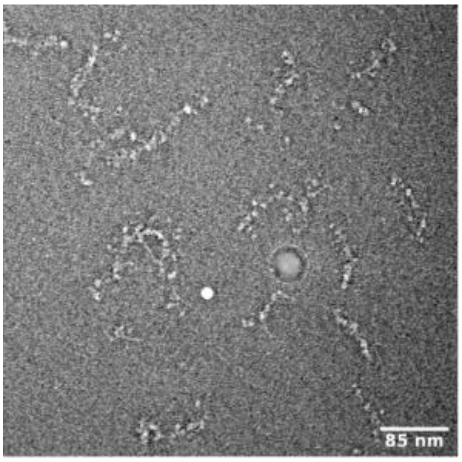

Figure S2: Negative stain electron micrograph of N-terminal fused superfolder GFP (sfGFP) to NX11 protein (sfGFP-NX11) complexed with 250 bp DNA (1.5 ng/μL) assembled at 0.5X molar excess in PBS and incubated overnight at room temperature. Scale bar: 85 nm.

> **Reviewer 1:** Is the "a periodicity" referred to here being “pitch” (Y-axis coordinates in Figure 3h)? In addition, “pitch” is usually applied only for one helix, not directly between two helixes (there is confusion about the use of “pitch” in Figure 3j).

We thank the reviewer for raising this point. We acknowledge that the term “pitch” is typically used to describe helices, which may have led to some confusion. The “periodicity” mentioned in the AFM analysis (**Fig. 3h**) indeed corresponds to the “pitch” and refers to the major “periodicity” observed.

Changes made:

- We have updated the y-axis label of **Fig. 3h** to "periodicity" for consistency.

> **Reviewer 1:** Does “pitch” here mean the same thing as helical pitch (Figure 3j, 34 Å or 68 Å) for cryo-EM? Why is there such a big difference?

We agree with the reviewer: “pitch” is not the correct term here and should be “periodicity”. The periodicity observed in Figure 3j is not the same as the helical pitch measured by cryo-EM. In AFM, the periodicity refers to larger-scale structural features detected along the assembled fibers, whereas in cryo-EM, the helical pitch corresponds to the super helical pitch of the NX11 proteins. The observed difference arises because AFM captures topographical patterns over extended lengths because longer fibers are studied, while cryo-EM resolves the intrinsic helical organization at the atomic level.

> **Reviewer 1:** How many NX11s does "a periodicity" correspond to?

The minor periodicity (~7 ± 2 nm) measured in AFM likely corresponds to a single NX11 molecule bound to DNA. Since a single NX11 makes almost a full helical turn, this minor periodicity matches the helical pitch of NX11 observed in cryo-EM. The major periodicity (~40 ± 10 nm) appears to correspond roughly equivalent to ~5 NX11 molecules, reflecting a larger repeating structural unit along the assembled fibers.

> **Reviewer 1:** The current data do not support "a protein filament helically wraps around the dsDNA using a binding mode similar to that of natural TALE proteins" (abstract, lines 30-32).

We acknowledge the reviewer’s concern and agree that current resolution of the data does not provide direct evidence for the binding mode of NX11-DNA. While our data suggest a helical assembly around DNA, the exact binding mode is not confirmed.

Changes made:

- <u>Lines 30-32 in abstract revised to</u>: "*In these structures, a protein filament helically wraps around the dsDNA similar to natural TALE proteins.*"

> **Reviewer 1:** It is recommended to show the "double-coiled assembly" in detail, which is significantly different from the previous crystal structure (Figure 1a). In fact, in the case of Type II assemblies, it may also be "double-coiled assembly" (a two-start helix).

We thank the reviewer for this suggestion. The reviewer is correct that we cannot exclude the option that type II is a two-start helix forming a double-coil assembly. In response to the reviewer's suggestion, we have expanded our analysis of NX11–DNA assemblies to include additional data analysis to show rare assembly types as well as models illustrating the possible "double-coiled" architecture for type II.

Changes made:

- Included **new Fig. S12** including expanded cryo-EM analysis of NX11-DNA assemblies.
    - Cryo-EM 2D class averages of all observed assembly types (I, II, and rare types III and IV, <1%) are shown, with their corresponding power spectra and distinguishing features highlighted (**Fig. S12A, D, E, G, I**).
    - AlphaFold3 predictions of NX11–DNA assemblies, revealing bi-directional propagation and termination events (**Fig. S12B–C**).
    - Ab initio 3D reconstructions of Type I and Type II assemblies (**Fig. S12F, H, J**), including evidence for a double-coil architecture in Type II.
    - Schematic models for parallel versus anti-parallel double-coil Type II assemblies (**Fig. S12K–L**).
- Added section to Results section (lines 261-266): "*More detailed cryoEM analysis (**Fig. S12**) highlights the presence of more rare distinct assembly states (Type III, IV). Type I and II assemblies account for ~50% of the total particles each, whereas Type III and IV represent <1%. Across all 2D class averages, there is a consistent absence of discernible α-helical secondary structure, suggesting that assembly occurs in a highly flexible and non-specific manner with pitches that deviate markedly from the design model.*"

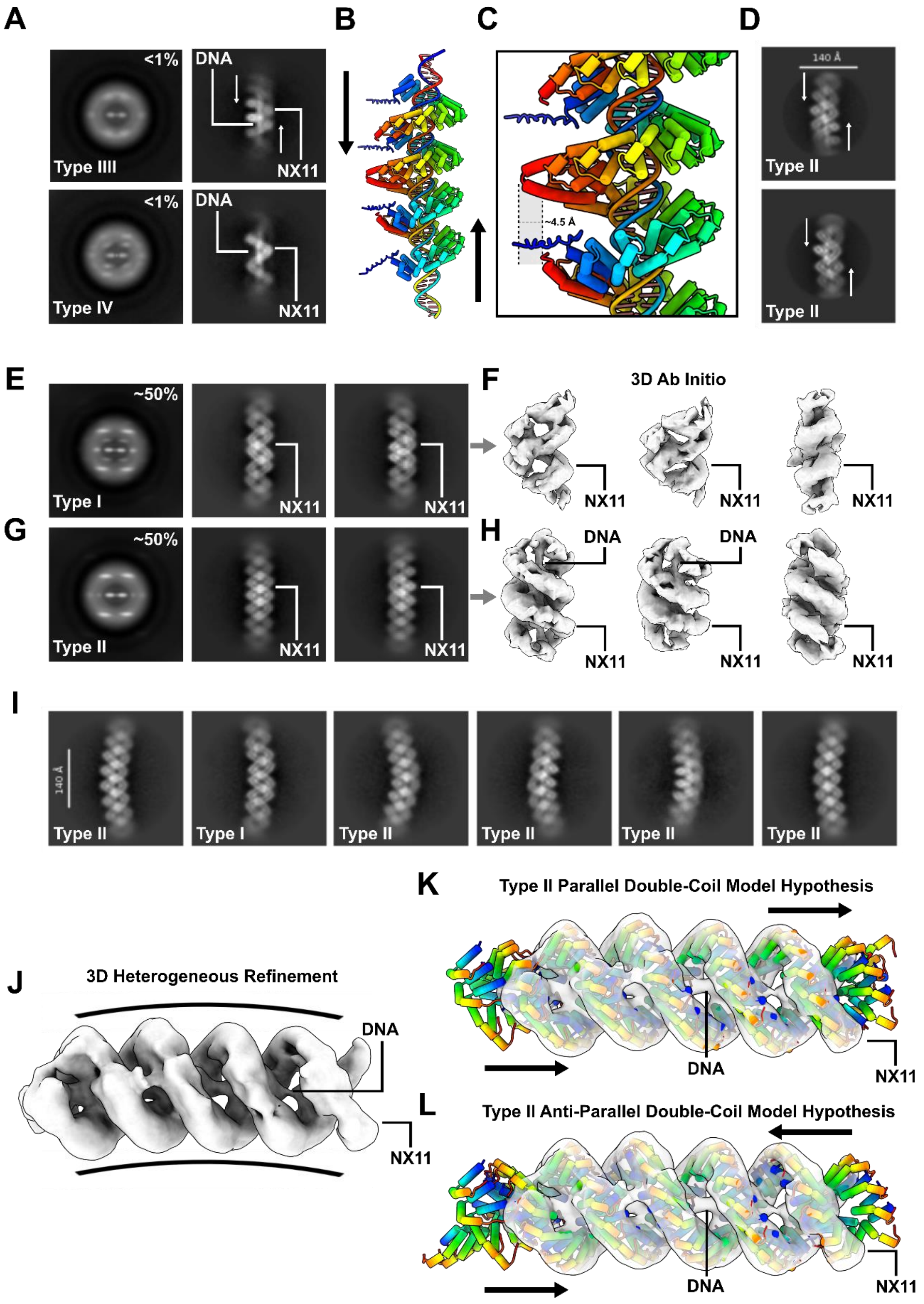

Figure S12. Cryo-EM analysis of NX11–DNA assemblies. (A) Representative 2D class averages of two rare assembly types (<1% of total particles). *Top*: Type III assemblies, which display unique power spectra resembling the intended design model but exhibit "switching" of NX11 assembly directionality. *Bottom*: Type IV assemblies, which also show unique power spectra and appear related to Type III, but with a longer apparent helical pitch, suggestive of an intermediate assembly state prior to formation of a double-coil geometry. **(B)** AlphaFold3 prediction of NX11 in complex with the DNA template used in this study, revealing bi-directional assembly propagation along the DNA. Assemblies

terminate when colliding fibers of opposing directionality meet. **(C)** Zoomed-in view of the predicted termination point between colliding NX11 fibers, highlighting a ~4.5 Å difference in height of NX11 C-termini relative to adjacent fibers along the DNA major groove. **(D)** Select 2D class averages of Type **II** assemblies showing bi-directional assembly consistent with AlphaFold3 predictions, including subtle differences in fiber height. This may underlie the periodic height variation observed in AFM measurements of NX11–DNA assemblies. **(E)** Representative 2D class averages and power spectra of Type **I** assemblies, viewed along two principal axes, showing apparent helical pitches of 34 Å and 68 Å. **(F)** Ab initio 3D reconstruction of Type **I** assemblies, which appear flattened along one axis due to poor angular sampling; no DNA density is observed. **(G)** Additional 2D class averages and power spectra of Type **I** assemblies from multiple tilted views. **(H)** Ab initio 3D reconstruction of Type **II** assemblies, showing clear double-coil architecture with DNA density at the fiber core. **(I)** Evidence of flexibility in Type **I** and Type **II** assemblies, with continuous variation in fiber geometry. **(J)** Heterogeneous refinement performed in C1 symmetry using ab initio maps, yielding reconstructions consistent with Type **II** double-coil assemblies but also revealing bends along the fiber. (K–L) Schematic hypotheses for Type **II** assembly states. **(K)** *Parallel Double-Coil* model, in which both fibers propagate in the same direction. (L) *Anti-Parallel Double-Coil* model, in which opposing fibers propagate toward each other, terminating upon collision. Taken together, these data highlight the presence of multiple distinct assembly states (Type **I**, **II**, **III**, IV) with substantial structural heterogeneity. Type **I** and **II** assemblies account for ~50% of the total particles each, whereas Type **III** and IV represent <1%. Across all 2D class averages, there is a consistent absence of discernible α-helical secondary structure, suggesting that assembly occurs in a highly flexible and non-specific manner with pitches that deviate markedly from the design model.

> **Reviewer 1:** As mentioned by authors, there are "150 2D class averages" (Line 456). What percentage of segments does the 2D classification shown in Figure 3j represent?

We thank the reviewer for pointing this out. The reference to "150 2D class averages" was an error. In fact, the final 2D classification used to generate the power spectra was performed with 100 classes, of which 87 contained particles and 13 were empty. The 2D class averages shown in **Fig. 3j** represents 1,363,928 particles in total, of which 795,040 (58%) were Type I and 568,888 (42%) were Type II. A very small fraction (<1%) of particles remained unassigned due to poor quality. Thus, the averages in **Fig. 3j** represents essentially the entire dataset (99%).

Changes made:

- Line 476 in Results section revised to: "*..., representing 1,363,928 particles in total, of which 795,040 (58%) were Type I and 568,888 (42%) were Type II.*"

> **Reviewer 1:** I suggest authors improve the quality of the cryo-EM data and do a 3D reconstruction (or local reconstruction) with sufficient resolution to elucidate the relationship between the protein helix and the DNA helix.

We appreciate the reviewer's suggestion and fully recognize the importance of elucidating the relationship between the protein and DNA helices. Despite considerable efforts — including the acquisition of extensive cryo-EM datasets and multiple rounds of both global and local 3D reconstructions for Type I and Type II assemblies, and consultations with several cryo-EM experts— we have not achieved the resolution necessary to unambiguously resolve either the DNA or protein structures. We believe this challenge appears to stem from inherent structural heterogeneity and/or flexibility within the assemblies. In particular, our data suggest the presence of multiple distinct assembly states, varying tilt angles of the

helices that make it hard to distinguish between them, and evidence of non-processive initiation events, where assembly start points differ rather than extending from a uniform nucleation site. These features, combined with considerable internal flexibility, result in both discrete and continuous heterogeneity that deviates substantially from the design model. As a consequence, achieving a high-resolution reconstruction by cryo-EM is extremely challenging for one state, let alone the ensemble of possible states in this system. As we point out in the discussion section, natural TALE proteins are known to exhibit intrinsic structural plasticity in solution (lines 319-329 and ref. 22), which may also explain some of the heterogeneity we observe.

> **Reviewer 1:** Figure 1a: Where is the CTR domain on the illustration?

We thank the reviewer for pointing this out. We have now added a label to **Figure 1a** that indicates the CTR in the illustration.

> **Reviewer 1:** Lines 128-129: To support “eluted as a monomer from size-exclusion chromatography (SEC)”, please use SEC protein standard for comparison.

We appreciate the reviewer’s suggestion. To support the statement that the protein elutes as a monomer in size-exclusion chromatography (SEC) we have included a calibration curve from Cytiva on a Superdex 200 Increase 10/300 gl column used.

Changes made:

- Included **new Fig. S18**, which shows several high molecular weight protein standards from on a Superdex S200 Increase 10/300 gl (Cytiva) columns.

Column:	Superdex 200 Increase 10/300 GL
Sample:	1. Thyroglobulin (M_r 669 000), 3 mg/mL<br>2. Ferritin (M_r 440 000), 0.3 mg/mL<br>3. Aldolase (M_r 158 000), 3 mg/mL<br>4. Conalbumin (M_r 75 000), 3 mg/mL<br>5. Ovalbumin (M_r 44 000), 3 mg/mL<br>6. Carbonic anhydrase (M_r 29 000), 3 mg/mL<br>7. Ribonuclease A (M_r 13 700), 3 mg/mL
Sample volume:	500 µL
Buffer:	PBS (0.01 M phosphate buffer, 0.14 M NaCl, pH 7.4)
Flow rate:	0.5 mL/min
System:	ÄKTA explorer

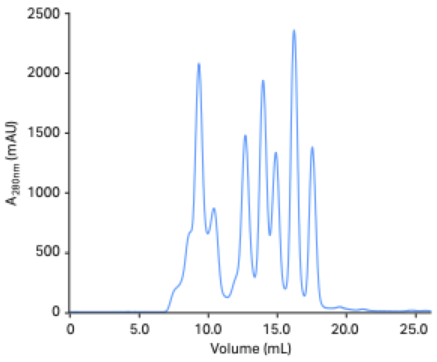

Figure S18. Superdex S200 Increase 10/300 gl (Cytiva) column calibration data. *From: “SIZE EXCLUSION CHROMATOGRAPHY: Superdex 200 Increase columns.”* Cytiva, MilliporeSigma, [no

publication date], https://cdn.cytivalifesciences.com/api/public/content/digi-16542-original. Accessed 17 Aug. 2025.

> **Reviewer 1:** Line 257: It is also preferable to use a platform-free antigen for direct comparison.

We acknowledge the reviewer's point. We do not expect significant immunogenic response from platform-free antigen as we expect the peptide to be cleared rapidly. We have included a platform-free control that carries a similar antigen on an icosahedron nanoparticle (I53.50_dn5-Majorx5). In addition, we have now also included a negative control (naive) mice group showing that titers elicited are a result of immunogens introduced.

Changes made:

- Included sentence to Results section (lines 307-311): "*Because NX11, DNA, or the naked NX11–DNA complex are not expected to elicit PfCSP-specific immune responses in the absence of an antigen, and unassembled peptide would likely be weakly immunogenic (ref. 36), we concentrated on comparing the antigen-displaying constructs and included benchmark and naïve groups to confirm the specificity of the response.*"
- Added ref. 36: Bachmann, M. F. & Jennings, G. T. Vaccine delivery: a matter of size, geometry, kinetics and molecular patterns. *Nat Rev Immunol* **10**, 787–796 (2010).
- Updated **Fig. 4d** with the negative control (naive) mice group.

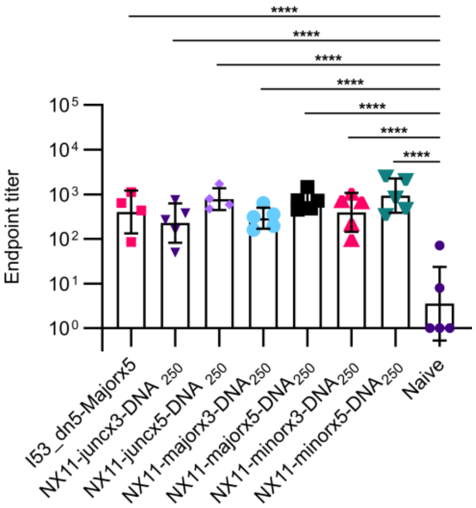

Figure 4d. Endpoints titers (OD=1.0) of anti-PfCSP antibody in the sera of immunized mice (n=5 per group), measured by ELISA. Statistical significance: **** $p < 0.0001$ (unpaired two-tailed Student's t-test).

Comments from Reviewer 2:

> **Reviewer 2:** While de Haas et al. show the versatility of the templating effect by using DNA with different lengths, the choice of the particular length (48, 50, 250, 750 or 1k bp) used is not reasoned, nor if the length potentially impacts the quality of the assembly and antigen properties.

We appreciate the reviewer's comment and acknowledge we did not provide rationale for the choice of DNA lengths used.

Long DNA fragments (>250 bp, NoLimits Thermo Fisher) were chosen for AFM and EMSA, as they are easier to visualize, while 48bp DNA HPLC-grade oligomers (IDT) were preferred for cryo-EM, as their increased stiffness, because of to the short persistence length of DNA, improves particle alignment and averaging. Similarly, for light scattering experiments, shorter DNA was chosen because it behaves more like a spherical-like particle. For immunogenicity studies, we selected pharmaceutical grade with NoLimits DNA of 250bp because it had low endotoxin levels, forms particles with lengths (~40 nm) similar to diameter of I53.50 nanoparticles and present a comparable number of immune epitopes per particle (~70 for NX11-DNA_{250} versus 60 for I53_dn5_majorX5).

We acknowledge and agree that larger lengths of DNA structures may introduce higher possibility of defects in the protein coating, as we observe in AFM (**Fig. 3** shows a major periodicity of ~40 nm). These defects may be indeed expected to impact assembly quality and antigen properties.

Changes made:

- <u>Revised sentence (lines 213-216) to</u>: "*We speculate that the former is an effect of slight mismatch between the NX11 helical twist and the helical twist of the DNA, which potentially builds up frustration in the DNA double helix, ultimately leading to increased heterogeneity in the assembly*."

> **Reviewer 2:** Following their assumption of 1 NX per bp, none of the chosen templates matches exactly with NX11, which makes one wonder about the protein coverage especially at the end of the dsDNA template.

The reviewer is correct that the chosen DNA templates do not exactly match the expected NX11 binding footprint. This may leave a small number of base pairs at the DNA ends exposed, or result in NX11 binding only partially at those positions. However, we do not expect these minor mismatches to negatively impact the assembly, since the majority of the footprint is preserved and NX11 binding remains stable overall.

> **Reviewer 2:** Additionally, a possible mismatch between the helical twist of NX11 and the dsDNA is proposed. How does the frequency of such mismatch occurrence depend on the length of the template DNA? This mismatch could be one of the reasons preventing 3D reconstruction.

We agree with the reviewer that a possible mismatch between the helical twist of NX11 and dsDNA indeed likely contributes to structural heterogeneity potentially hindering successful 3D reconstruction.

We reason that larger DNA lengths lead to more defects, as we have seen in our AFM studies (see ~40 nm major periodicities in Figure 3). Therefore for high-resolution cryo-EM work we selected a HPLC-grade DNA template of 48 bp (for 4 NX11 proteins). Still, our cryo-EM analysis and identification of Type I and Type II 2D class averages, suggest different binding modes of NX11, which we attribute to a high degree of inherent structural plasticity of NX11 proteins.

Changes made:

- Expanded the Discussion section (lines 321-326): "*Since this assembly process is stochastic and requires structural adaptation this could introduce local defects, which we hypothesize complicates high-resolution structural studies when bound to DNA, especially for longer DNA sequences.* "

> **Reviewer 2:** The assembly was proposed to consist of two phases, rapid nucleation and reorganization. Since the assembly is sequence-independent, what is the driving force of the nucleation?

The driving force of nucleation in the assembly process are primarily ionic and hydrogen bonding interactions on NX11 between residues Lys15, Arg10, Lys9 and Gln15 and phosphate atoms on the DNA backbone (**Fig. 1**).

Changes made:

- Inserted (line 143): *"(driven primarily by electrostatic interactions)"*

> **Reviewer 2:** Comparing the excess screening gels of NX11 and NX5 for excesses below 3× showed smear for NX11 but rather discrete bands for NX5. Why do the two repeat proteins behave differently and how does it influence the assembly?

The more pronounced smearing observed for NX11 on EMSA suggests greater heterogeneity in its DNA-bound states compared to NX5. Structurally, NX11 binds nearly a full turn around the DNA helix. In contrast, NX5 engages with only about half a turn of the DNA and has lower DNA-binding affinity per monomer. This reduced affinity may cause NX5 to more efficiently re-organize on the DNA and achieve more-defined coverage and therefore charge neutralization.

> **Reviewer 2:** Moreover, do the proteins have a directionality which would contribute to the observed mismatches? A more insightful discussion on the assembly would be beneficial for the manuscript.

The 1D diffusion behavior of native TALE proteins on DNA has been shown to be unbiased directional (no preferred direction) via direct observation in single molecule fluorescence

experiments. The authors show that TALEs slide and hop on the DNA while searching their specific target (ref. 42).

We expect similar behaviour of NX11 proteins. But at high local concentrations on the DNA, NX11-NX11 interactions may slow down the diffusion and this is a nucleation event for elongation of NX11 on the DNA fiber in either direction. We expect that given the plasticity of the NX11 structure, some structural re-organization is required to efficiently form protein-protein interactions. Because the assembly is a stochastic process, mismatches and slow structural re-organization can lead to the heterogeneity observed in AFM and EM.

Changes made:

- Added to discussion section (lines 317-322): "*Natural TALEs show bi-directional diffusion along the DNA during their target search mode (ref. 42). For NX11, assemblies likely terminate when colliding fibers of opposing directionality meet. This could act as a nucleation point for elongation of the NX11 along the DNA in either direction. Since this assembly process is stochastic and requires structural adaptation this could introduce local defects, which we hypothesize complicates high-resolution structural studies when bound to DNA, especially for longer DNA sequences.*"

> **Reviewer 2:** The comparison of Type I and Type II assemblies is somewhat confusing, and no actual reason is given. The authors simply indicate that Rosetta and AlphaFold2 disagree in their predictions in a way that happens to match the experimental finding, but it does not explain the underlaying reason why two different types are observed.

We acknowledge the reviewer's concern and agree that the underlying reason for the coexistence of Type I and Type II assemblies requires further discussion.

Due to the intrinsic flexibility of native TALE-like structures, this may allow NX11 to adopt different super-helical pitches (discussion lines: 300-307). Our hypothesis is that plasticity in the super-helical pitch allows NX11 to stretch and match the DNA double-helical pitch of ~64 Å, skipping every second major groove. This would create enough space for the formation of a parallel or anti-parallel double-coil fiber, leading to a Type II assembly. In contrast, Type I assembly follows a single major groove pitch of ~34 Å (as demonstrated by its power spectrum) and is likely a two-start helix. We have expanded our cryo-EM data analysis which shows that type II are likely also double-coil assemblies, either parallel or anti-parallel.

Changes made:

- Included **new Fig. S12** including expanded cryo-EM analysis of NX11-DNA assemblies.
    - Cryo-EM 2D class averages of all observed assembly types (I, II, and rare types III and IV, <1%) are shown, with their corresponding power spectra and distinguishing features highlighted (**Fig. S12A, D, E, G, I**).

    - AlphaFold3 predictions of NX11–DNA assemblies, revealing bi-directional propagation and termination events (**Fig. S12B–C**).
    - Ab initio 3D reconstructions of Type I and Type II assemblies (**Fig. S12F, H, J**), including evidence for a double-coil architecture in Type II.
    - Schematic models for parallel versus anti-parallel double-coil Type II assemblies (**Fig. 12K–L**).
- <u>Added to Discussion section (lines 312-316)</u>: "*Because NX11 exhibits plasticity in its super-helical pitch, allowing it to stretch and match the DNA double-helical pitch of ~64 Å, skipping every second major groove. This would create enough space for the formation of a double-coil fiber, leading to the Type II assembly.*"

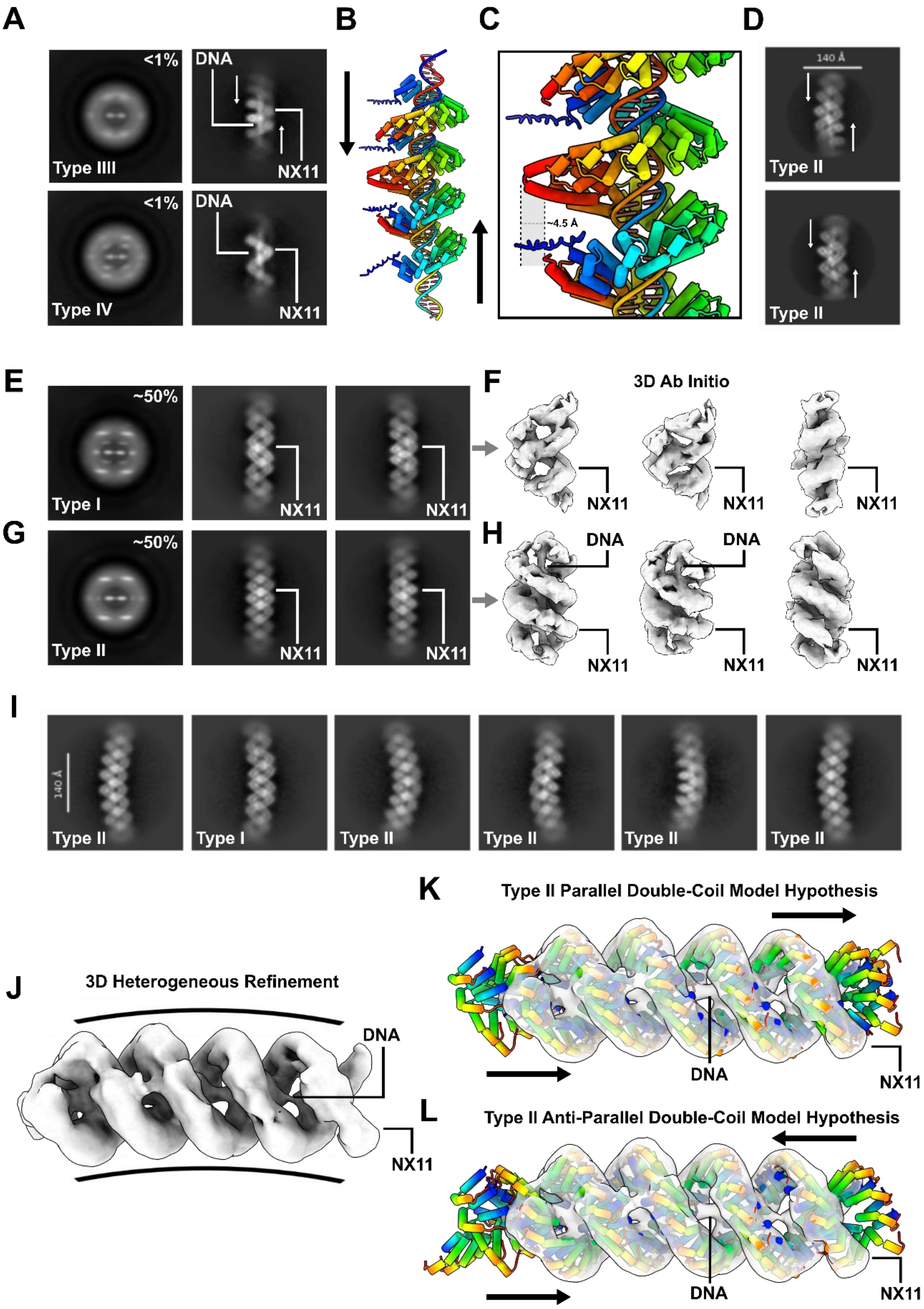

Figure S12. Cryo-EM analysis of NX11–DNA assemblies. (A) Representative 2D class averages of two rare assembly types (<1% of total particles). *Top*: Type III assemblies, which display unique power spectra resembling the intended design model but exhibit "switching" of NX11 assembly directionality. *Bottom*: Type IV assemblies, which also show unique power spectra and appear related to Type III, but with a longer apparent helical pitch, suggestive of an intermediate assembly state prior to formation of a double-coil geometry. **(B)** AlphaFold3 prediction of NX11 in complex with the DNA template used in this study, revealing bi-

directional assembly propagation along the DNA. Assemblies terminate when colliding fibers of opposing directionality meet. **(C)** Zoomed-in view of the predicted termination point between colliding NX11 fibers, highlighting a ~4.5 Å difference in height of NX11 C-termini relative to adjacent fibers along the DNA major groove. **(D)** Select 2D class averages of Type II assemblies showing bi-directional assembly consistent with AlphaFold3 predictions, including subtle differences in fiber height. This may underlie the periodic height variation observed in AFM measurements of NX11–DNA assemblies. **(E)** Representative 2D class averages and power spectra of Type I assemblies, viewed along two principal axes, showing apparent helical pitches of 34 Å and 68 Å. **(F)** Ab initio 3D reconstruction of Type I assemblies, which appear flattened along one axis due to poor angular sampling; no DNA density is observed. **(G)** Additional 2D class averages and power spectra of Type I assemblies from multiple tilted views. **(H)** Ab initio 3D reconstruction of Type II assemblies, showing clear double-coil architecture with DNA density at the fiber core. **(I)** Evidence of flexibility in Type I and Type II assemblies, with continuous variation in fiber geometry. **(J)** Heterogeneous refinement performed in C1 symmetry using ab initio maps, yielding reconstructions consistent with Type II double-coil assemblies but also revealing bends along the fiber. (K–L) Schematic hypotheses for Type II assembly states. **(K)** *Parallel Double-Coil* model, in which both fibers propagate in the same direction. (L) *Anti-Parallel Double-Coil* model, in which opposing fibers propagate toward each other, terminating upon collision. Taken together, these data highlight the presence of multiple distinct assembly states (Type I, II, III, IV) with substantial structural heterogeneity. Type I and II assemblies account for ~50% of the total particles each, whereas Type III and IV represent <1%. Across all 2D class averages, there is a consistent absence of discernible α-helical secondary structure, suggesting that assembly occurs in a highly flexible and non-specific manner with pitches that deviate markedly from the design model.

> **Reviewer 2:** The controls are poorly designed in the immunogenicity studies. For example, NX11 alone, JuncX3/5 alone, DNA alone, plain NX11-DNA250 complex, a commercially established positive control and a negative control must be added alongside the studied samples.

We thank the reviewer for raising this important point regarding the design of controls.

NX11 alone, DNA alone, and the naked NX11–DNA complex are not expected to elicit a CSP-specific immune response in absence of an immunogen. We expect JuncX3/5 peptide alone is also unlikely to generate a strong response, as we expect it is weakly immunogenic. To ensure robustness of our conclusions, we have included a platform-independent immunogen (the icosahedron nanoparticle I53.dn5_majorX5) as a positive control, which elicited a similar response to the fiber complexes. While a naive (untreated) mouse group was not included in the original study, we have now added this negative control. Importantly, the naive group confirms that the antigen-specific immune responses observed in all experimental groups are statistically significant (****, $p < 0.0001$). With this addition, we believe the study now incorporates a complete set of appropriate controls.

Changes made:

- <u>Included sentence to Results section (lines 307-311)</u>: "*Because NX11, DNA, or the naked NX11–DNA complex are not expected to elicit PfCSP-specific immune*

responses in the absence of an antigen, and unassembled peptide would likely be weakly immunogenic, we concentrated on comparing the antigen-displaying constructs and included benchmar*k and naïve groups to confirm the specificity of the response*."

- **Fig. 4d** is updated to include a naive mouse negative control group, including statistical analysis.

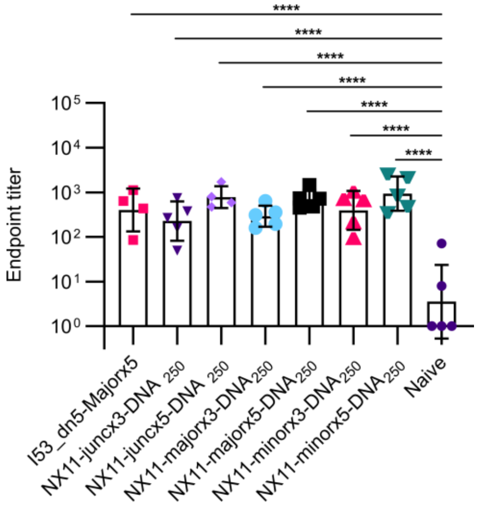

Figure 4d. Endpoints titers (OD=1.0) of anti-PfCSP antibody in the sera of immunized mice (n=5 per group), measured by ELISA. Statistical significance: **** $p < 0.0001$ (unpaired two-tailed Student's t-test)**.**

> **Reviewer 2:** Furthermore, the authors do not present any attempts to characterize the in vivo stability of the NX-DNA complexes. Establishing the structural integrity of the studied complexes is crucial for the validating the results that organizing the NXs matters.

We thank the reviewer for highlighting the importance of in vivo stability. We agree that understanding the structural integrity of NX-DNA complexes in biological settings will be an important step for future translational development. However, directly assessing nanoscale assembly states *in vivo* is technically challenging with current methods.

Importantly, the goal of this study was not to provide a comprehensive immunogenicity evaluation, but rather to establish a proof-of-concept: that defined nanoscale organization of NXs can be structurally validated *in vitro* and is sufficient to elicit robust immune responses. We have now added a sentence to the discussion section to acknowledge this limitation and to emphasize that future studies will be needed to address stability in vivo.

Changes made:

- Added to the Discussion section (lines 340-342): "*Direct assessment of NX–DNA complex stability in vivo remains technically challenging, and we anticipate that future studies will be needed to address this important aspect of translational development*."

> **Reviewer 2:** DLS data is not properly presented. Why is the y-axis Amplitude %? This should be volume distribution or preferably scattering intensity (intensity distribution).

We apologise for the confusion. The label “*Amplitude (%)*” actually represents the intensity distribution.

Changes made:

- Updated the y-axis label in **Fig. 2d** and **Fig. 4c** to: “*Intensity (%)*”

> **Reviewer 2:** All measure distributions in Figures 2 and 4 should be accompanied by Z-average size and PDI values as well as the measured autocorrelations.

We agree with the reviewer that for full transparency and accuracy these values should be reported.

Changes made:

- Added **new Fig. S2**, showing the autocorrelation data from **Fig. 2d** to Supplementary Information.
- Added **new Fig. S14**, showing Z-AVG diameter, PDI values and measured autocorrelations to the Supplementary Information.

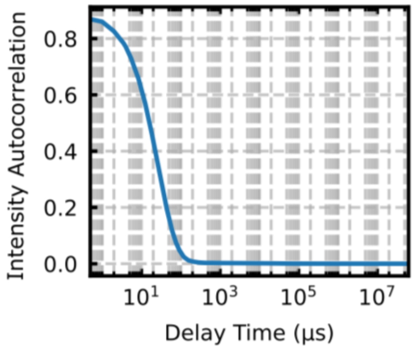

Figure S2. Intensity autocorrelation vs. delay time for data presented in Figure 2d. Z-AVG diameter was 18.3 nm and the PDI was 0.12.

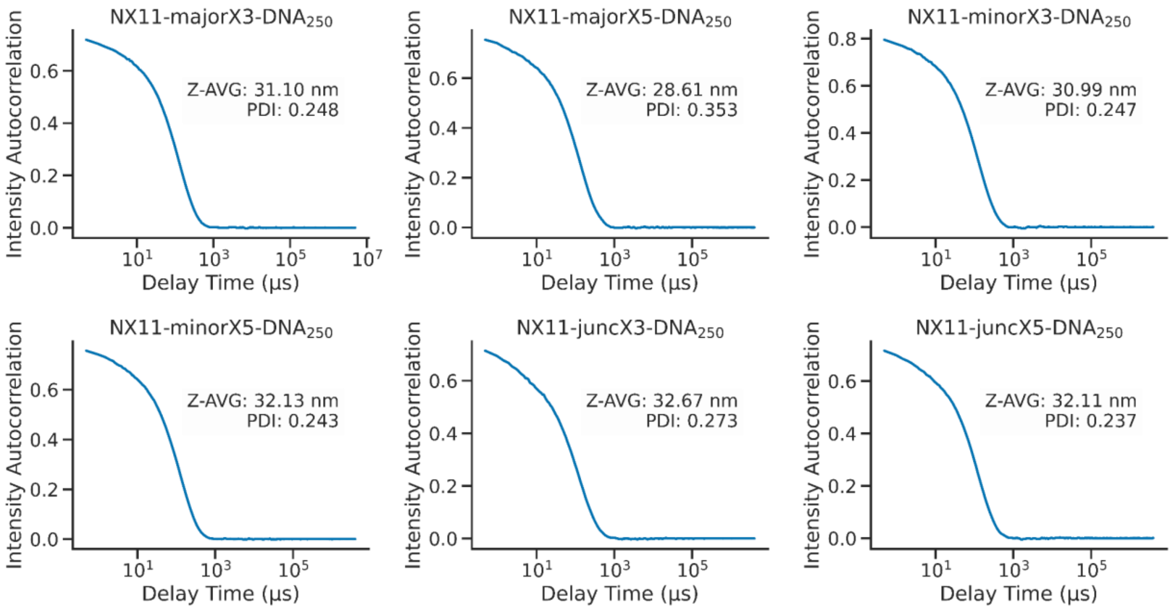

Figure S14. Intensity autocorrelation vs. delay time for data presented in Figure 4c.

> **Reviewer 2:** If assemblies templated by DNA with lengths shorter than 50 bp are susceptible to side-by-side dimerization, how conclusive is the DLS data obtained for NX11-DNA48?

The reviewer raises a good point.

It is true that the NX11-DNA_{48} DLS data in **Fig. 2d** may contain contributions from side-by-side dimers. DLS does not have the resolution to directly distinguish between monomeric or dimeric fibers, as the expected differences in hydrodynamic diameter are too small to resolve. The DLS data in **Fig. 2d** is presented to demonstrate the lack of higher order aggregates.

> **Reviewer 2:** Can the authors elaborate on why this phenomenon was only observed for short DNA templates, since the weak yet repetitive interactions along the DNA fibre" would expectedly be also present in longer DNA-protein hybrids.

This phenomenon is primarily influenced by the flexibility of the DNA template, which is governed by its persistence length (approximately 100 bp or ~40 nm contour length). DNA templates of 50 bp or shorter remain relatively stiff, allowing side-by-side interactions to stack effectively. In contrast, we suspect longer DNA templates exhibit greater flexibility, introducing steric hindrances that reduce the likelihood of ordered side-stacking interactions to form. Additionally, deformations in fiber formation such as those observed in AFM data (Fig. 3) can introduce heterogeneity in stacking interactions, a factor we suspect becomes more pronounced as DNA length increases.

Changes made:

- Added sentence (222-225): "*For shorter templates, which are below the persistence length of DNA, the duplex remains relatively rigid and can engage in stackable contacts*

that favor dimer formation, whereas longer templates introduce flexibility that reduces the likelihood of such ordered stacking."

Reviewer 2: Fig. 2f: How long is Benzonase active at the tested conditions, i.e., how informative is for instance the ON incubation?

Benzonase remains highly active even after prolonged incubation at 37°C, as confirmed by the manufacturer. According to Sigma-Aldrich, extensive stability testing has shown that Benzonase retains >90% activity for several months under these conditions[1] . This suggests that the overnight (ON) incubation would not result in significant loss of nuclease activity.

Changes made:

- Added to the Methods section (lines 444-445): "*Benzonase retains >90% activity for several months under these conditions (ref. 56)*."
- Added new ref. 56: *"Benzonase® Nuclease Q&A."* MilliporeSigma (Sigma-Aldrich), https://www.sigmaaldrich.com/US/en/technical-documents/technical-article/protein-biology/protein-lysis-and-extraction/answers-to-questions-about-benzonase-nuclease. Accessed 10 Oct. 2025.

Comments from Reviewer 3 & 4:

Reviewers 3 & 4: The data in Figure 3c shows very few data points and none above 2 nm. Yet, judging from the AFM images some of the showed structures are higher. In panels e/f the height profile should also include the bare substrate at the beginning and end of the profile to allow comparison to panel c. Now the data fluctuates between +/- 0.5 nm, which is close to the observed difference between DNA and NX11-DNA samples. Could the authors comment on this?

We thank the reviewer for this insightful observation. We agree that including the full height distribution and the substrate reference enhances the clarity of the data presentation.

Changes made:

- Updated **Fig. 3c** to include all individual data points in the boxplots (as also applied to **Fig. 3d** and **3h**).
- Added **new Fig. S7a**, which compares the line profiles of DNA and NX11–DNA samples with those of the respective bare substrates. This demonstrates that the observed height differences between the samples are significant relative to the underlying substrate roughness.

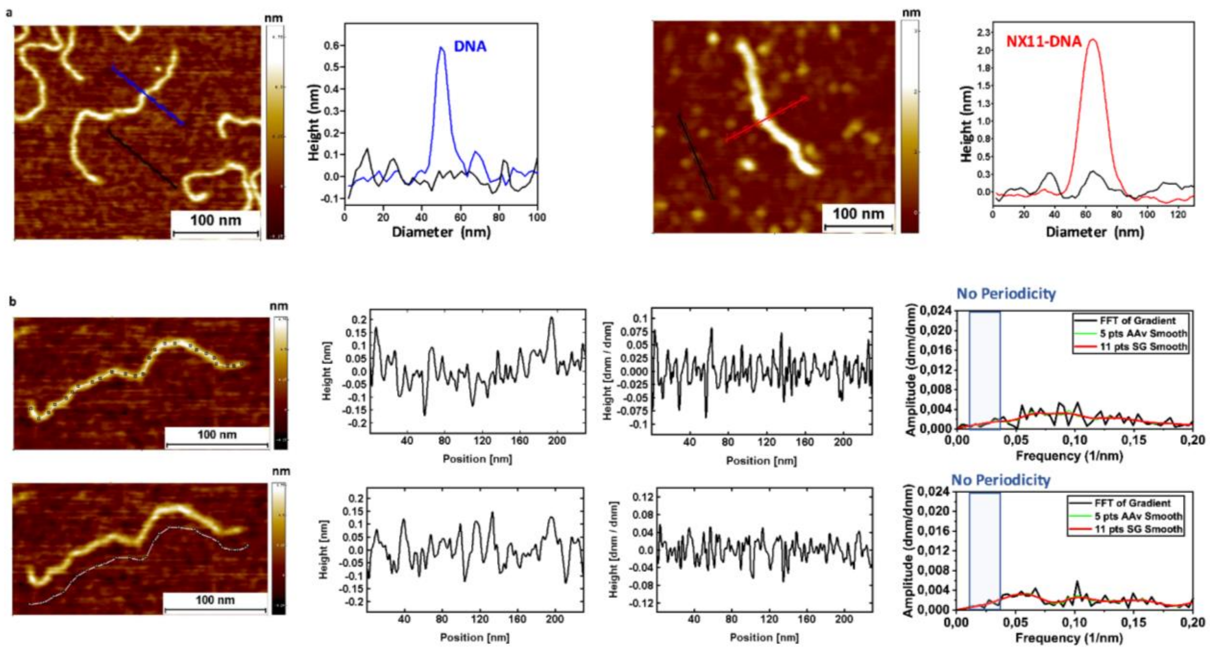

Figure S7. (a) Line profiles on the mica substrate for both DNA and NX11-DNA samples. (**b)** FFT analysis on DNA and bare mica without proteins.

Reviewers 3 & 4: Page 6, line 246-249: "...DNA250 into complexes at a 1.1× excess, and the assembly was confirmed by EMSA (Fig. S9)". Figure S9b in contrast refers to "...assembled with DNA100 at 3-fold excess." Please clarify the discrepancy.

We thank the reviewer for noticing this discrepancy. The correct value is 1.1× excess, and we have updated the **Fig. S9b** caption accordingly.

Changes made:

- **Fig. S9b** caption revised to: *"EMSA of NX11 variants displaying malaria antigens assembled with DNA_{100} (NoLimits, Thermo Fisher Scientific) at 1.1-fold excess."*

> **Reviewers 3 & 4:** The analysis of NX1121Q.5-DNA48 complexes shows two main particle populations. With which frequency does either of them occur, is there a preference for one of the populations? How do the authors imagine the formation of two NX11 fibrils wrapping the DNA and what causes it?

We thank the reviewers for these insightful questions. The two predominant populations are observed at comparable frequencies, with Type I particles representing 795,040 (58%) and Type II particles 568,888 (42%) of the total 1,363,928 classified particles. Thus, while both modes are well represented, there is a modest preference for Type I.

With respect to the two fibrils wrapping around the DNA, we propose that for Type II these arise from the intrinsic bi-directional propagation of NX11 along the DNA template. As shown in the **new Fig. S15K–L**, two possible double-coil models are consistent with the data: (i) a parallel double-coil, where both fibrils propagate in the same direction, and (ii) an anti-parallel double-coil, where fibrils of opposing polarity collide and terminate. AlphaFold3 predictions support this bidirectionality, and the observed heterogeneity in 2D classes and reconstructions suggests that both arrangements may coexist, reflecting the structural plasticity of NX11–DNA assemblies.

Changes made:

- Added **new Fig. S12**, which includes:
  - Schematic models and hypotheses for parallel and anti-parallel double-coil Type II assemblies (**Fig. S12K–L**).
  - *Ab initio* reconstructions of Type I and Type II assemblies **(Fig. S12F, H, J**), highlighting the double-coil architecture in Type II.
- Modified sentence in the Materials & methods section (line 520-523): *"Approximately 1.2 million particles were picked using the filament tracer, which were subsequently sorted into 100150 2D class averages, representing 1,363,928 particles in total, of which 795,040 (58%) were Type I and 568,888 (42%) were Type II."*

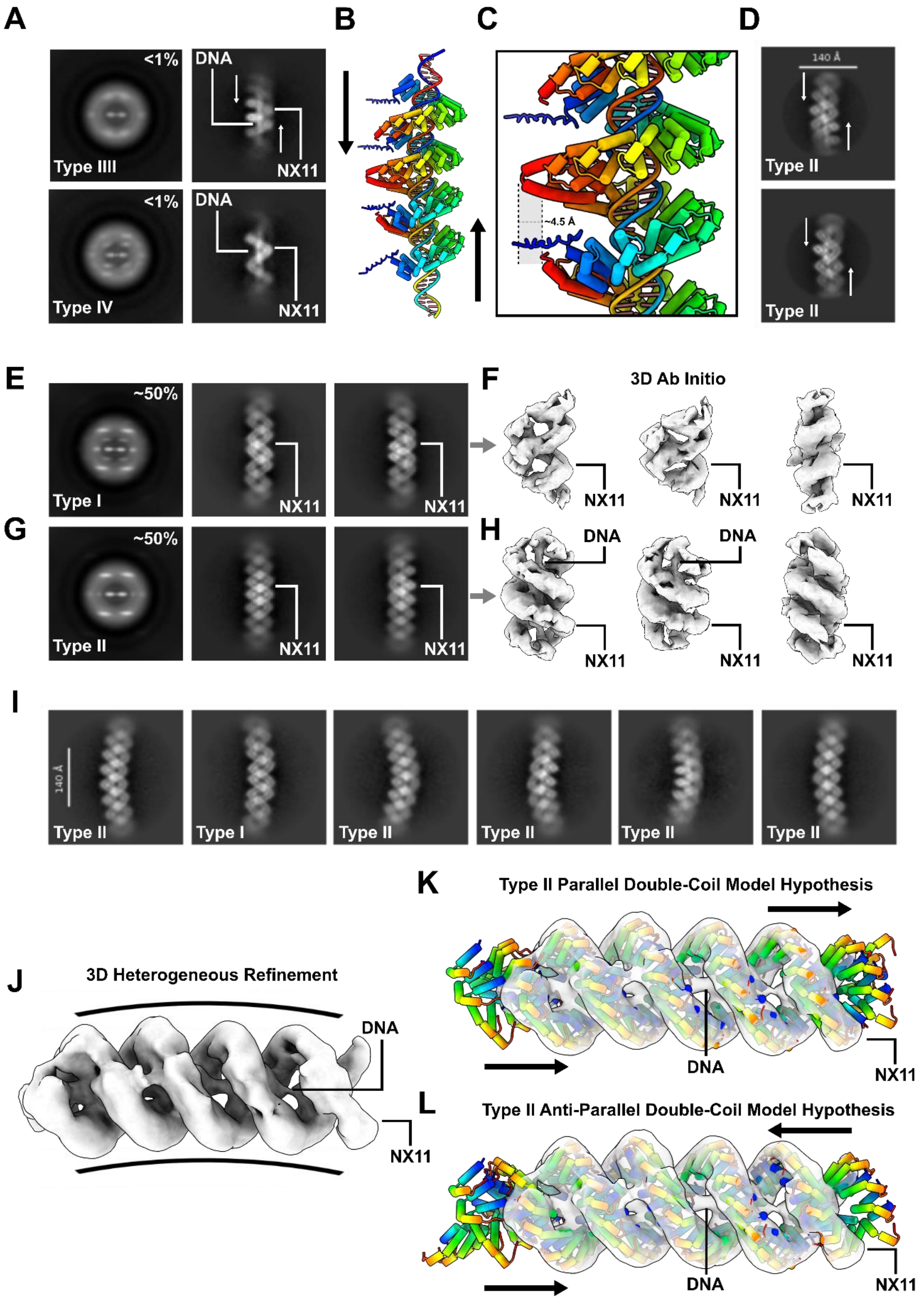

Figure S12. Cryo-EM analysis of NX11–DNA assemblies. (A) Representative 2D class averages of two rare assembly types (<1% of total particles). *Top*: Type III assemblies, which display unique power spectra resembling the intended design model but exhibit "switching" of NX11 assembly directionality. *Bottom*: Type IV assemblies, which also show unique power spectra and appear related to Type III, but with a longer apparent helical pitch, suggestive of an intermediate assembly state prior to formation of a double-coil geometry. **(B)** AlphaFold3 prediction of NX11 in complex with the DNA template used in this study, revealing bi-directional assembly propagation along the DNA. Assemblies

terminate when colliding fibers of opposing directionality meet. **(C)** Zoomed-in view of the predicted termination point between colliding NX11 fibers, highlighting a ~4.5 Å difference in height of NX11 C-termini relative to adjacent fibers along the DNA major groove. **(D)** Select 2D class averages of Type II assemblies showing bi-directional assembly consistent with AlphaFold3 predictions, including subtle differences in fiber height. This may underlie the periodic height variation observed in AFM measurements of NX11–DNA assemblies. **(E)** Representative 2D class averages and power spectra of Type I assemblies, viewed along two principal axes, showing apparent helical pitches of 34 Å and 68 Å. **(F)** Ab initio 3D reconstruction of Type I assemblies, which appear flattened along one axis due to poor angular sampling; no DNA density is observed. **(G)** Additional 2D class averages and power spectra of Type I assemblies from multiple tilted views. **(H)** Ab initio 3D reconstruction of Type II assemblies, showing clear double-coil architecture with DNA density at the fiber core. **(I)** Evidence of flexibility in Type I and Type II assemblies, with continuous variation in fiber geometry. **(J)** Heterogeneous refinement performed in C1 symmetry using ab initio maps, yielding reconstructions consistent with Type II double-coil assemblies but also revealing bends along the fiber. (K–L) Schematic hypotheses for Type II assembly states. **(K)** *Parallel Double-Coil* model, in which both fibers propagate in the same direction. (L) *Anti-Parallel Double-Coil* model, in which opposing fibers propagate toward each other, terminating upon collision. Taken together, these data highlight the presence of multiple distinct assembly states (Type I, II, III, IV) with substantial structural heterogeneity. Type I and II assemblies account for ~50% of the total particles each, whereas Type III and IV represent <1%. Across all 2D class averages, there is a consistent absence of discernible α-helical secondary structure, suggesting that assembly occurs in a highly flexible and non-specific manner with pitches that deviate markedly from the design model.

Reviewers 3 & 4: Figure 4 shows multiple nsTEM images, which are not fully convincing. The quality seems lower than with AFM imaging. Why was AFM or cryo-EM not utilized for these samples?

Obtaining high-resolution nsTEM images for these samples is inherently challenging due to the difficulty in achieving appropriate contrast, as the particles do not stain well given their thin diameters. While AFM or cryo-EM could offer enhanced structural detail, these methods were not pursued due to bandwidth limitations. Nonetheless, particle assembly is well-supported by complementary techniques, including DLS (**Fig. 4c**) and EMSA (**Fig. S13**).

Reviewers 3 & 4: The assembly experiments and complex characterizations have been performed with 3× excess. It did not get entirely clear to me, why the excess was decreased to 1.1× for the antigen display studies. Following the argumentation given for NX11-1A/3A and looking at Fig. 2e/S3, NX11 appears with a slight smear at 1× excess which might suggest the presence of intermediate assembly species. How do complexes with 1.1× excess compare to complexes with 3× excess?

The excess was reduced to 1.1× in the antigen display studies to minimize the presence of unbound protein, which could contribute to immunogenicity. Importantly, EMSA data at 1.1× excess (**Fig. S13**) show relatively monodisperse bands, indicating that the assemblies are well-formed, similar to 3× excess, and do not exhibit large amounts of intermediate species under these conditions.

Reviewers 3 & 4: Additionally, the slightly different migration patterns of the anti-CSP NX11 designs may be attributed to variations in their net surface charge. Notably, no smearing is observed for anti-CSP NX11 at 1.1× excess in Figure S9, suggesting that intermediate species are not present under these conditions

We fully agree with the reviewers' comment.

Reviewers 3 & 4: Along the line, does the insertion of the epitopes into the loop region interfere with the assembly, for instance with the coverage of the protein on the DNA template or the coiling parameters?

This is an interesting thought. The insertion of epitopes into the loop region could potentially introduce additional flexibility or heterogeneity in the assembly, which might slightly influence the assembly properties, such as protein coverage on the DNA template or coiling parameters. However, this does not appear to significantly impact the overall assembly, as evidenced by the full coverage observed in nsTEM images and DLS (**Fig. 4b-c**) and the relatively monodisperse assemblies (**Fig. S13**).

Reviewers 3 & 4: Figure 4d, were the injected samples purified from excess/unbound protein?

The injected samples were not further purified from excess protein. Given that only a 10% excess of protein was used and the majority of the protein is bound, we proceeded without additional purification. We have clarified this now in the main text.

Changes made:

- <u>In the Method section (lines 512-513) added</u>: "*The 10% excess NX11 protein was not further purified from the complexes*."

Reviewers 3 & 4: Nucleic acid delivery is mentioned as a potential application. How strongly are the proteins bound to the DNA and how do the authors imagine the disassembly of the complexes/release of the DNA?

Natural TALEs bind DNA with low nanomolar affinity (Cuculis et al., 2019), and we expect our NX11 variants to exhibit similar binding strength. Controlled release could be achieved through pH-sensitive design for example, by introducing histidine residues at protein–protein and protein–DNA interfaces. Upon acidification in endosomal or lysosomal environments, protonation of these residues would induce charge repulsion, promoting disassembly of the NX11–DNA complex and facilitating DNA release.

Reviewers 3 & 4: The authors claim that for samples ("cylindrical objects") with a height less than 4 nm "non-linear underestimation" occurs, referencing Ref. 24. However, there is no such claim or proof made in that reference.

Thanks for noticing the wrong reference, we have corrected it.

Changes made:

- Changed ref. 24 to: "Ruggeri, F. S. *et al.* Identification and nanomechanical characterization of the fundamental single-strand protofilaments of amyloid α-synuclein fibrils. *Proc. Natl Acad. Sci. USA* 115, 7230–7235 (2018)."

> **Reviewers 3 & 4:** The authors used cantilevers with 5 N/m spring constant. It is not unreasonable to assume that this also could cause significant deformation of the DNA and protein complexes

We agree with the reviewer that the cantilever could lead to a mechanical deformation. Indeed, as widely reported, our measured height of DNA alone in air is well below 2 nm, because of the effects mentioned above as well as the deformation by the cantilever. Our aim was however to perform relative height comparison between DNA and NX11-DNA. We thus employed phase-controlled AFM imaging, maintaining a consistent phase regime (≈Δ20°), to assure similar deformation of different samples allowing accurate and precise comparison of their height.

Changes made:

- Added clarifying sentences to Results section (lines 201-202): *While tip-induced deformation can affect AFM measurements, our focus was on relative height comparison. To ensure consistent deformation across samples, we used amplitude modulation AFM under phase-controlled conditions (<Δ20°), as established in previous studies (ref. 23, 27–29). This approach ensures similar energy dissipation during tip-sample contact, enabling accurate comparison.*

> **Reviewers 3 & 4:** Electron microscopy results seem convincing and fit well with the AFM results, but some additional analysis could help to strengthen the results. (a) Performing the FFT analysis on DNA without NX11 proteins and (b) performing the FFT analysis along random paths on the mica substrate between the DNA (without proteins).

Following the suggestion, we performed FFT analysis on DNA and bare mica without proteins. As in other previous similar analyses (ref. 29), these data prove the absence of periodicity on the mica substrate and the DNA. Instead, NX11 shows a clear periodicity (Figure S5).

Changes made:

- Added sentence to Results section (lines 228-230): *"To validate that these periodic features are specific to NX11-DNA complexes and not artifacts of the substrate or DNA alone, we performed FFT analysis on bare DNA and mica surfaces without proteins (**Fig. S7b**). Consistent with previous studies (ref. 29), these controls showed no detectable periodicity. In contrast, NX11-DNA complexes exhibited clear and reproducible periodic patterns (**Fig. S8**), confirming that the observed periodicity is intrinsic to NX11 binding."*
- Added **new Fig. S7b** includes FFT analysis on DNA and bare mica without proteins.

- Added ref. 29: “Li, X., Pujari, S. P., van der Gucht, J., Zuilhof, H. & Ruggeri, F. S. Unravelling emergence of chirality in click-chemistry polymers down to the single-chain level. *Nature Communications* 16, 1–15 (2025).”

> **Reviewers 3 & 4:** From Fig. 3a, it seems that the bare DNA is also not of uniform height and it would be good to confirm that the periodicity is not something that comes from these corrugations. Furthermore, the mica substrate might also have some height fluctuations that could propagate and cause apparent height differences in the protein complexes. It is not too clearly visible, but there seem to be some corrugations of the mica, although the resolution of the image is not good enough to be sure.

We have proved above with the new Figure S16b that the mica substrate lack periodicity, with a roughness <0.1 nm when only DNA and of ~0.2 nm with DNA and protein deposited. This variability was accounted for in our total error of our height analysis (Figure 3c).

> **Reviewers 3 & 4:** Page 11, Line 413: “*cleaved the mica surface by etching*” I am not familiar with this procedure of cleaving by etching. It might be a good idea to provide a reference. Typically, mica is cleaved by exfoliating the top layers by use of adhesive tape.

We apologise for the confusion, we indeed cleaved the mica by exfoliating the top layers using adhesive tape, not via etching.

Changes made:

- Line 445-446 revised to: “*cleaved the mica surface by exfoliating the top layer using adhesive tape.*”

> **Reviewers 3 & 4:** Page 11, Line 421-424: The authors describe that the samples were imaged in a regime of “constant phase change”. Do the authors mean that the phase shift was constant? Meaning the imaging was performed by keeping the phase shift at a set value? I.e. phase modulation instead of the more common amplitude modulation? If so, why?

Yes, we measured keeping a constant phase change <Δ20°, to have similar energy dissipation during tip-sample contact. To keep this condition is not necessary to measure in phase modulation, and we used amplitude modulation AFM for accurate comparison of different samples as previously reported.

> **Reviewers 3 & 4:** Moreover, it would be good to provide the cantilever’s oscillation amplitude (in nm, if possible) and the amplitude set-point (which can be used to estimate tapping forces).

The cantilever’s free oscillation amplitude was ~35-30 nm, and an amplitude setpoint of ~25-20 nm (70-80%).

Changes made:

- Added to line 486: *"... and stiffness of 4.4 ± 0.1 N/m as estimated by the thermal sweep method. The cantilever's free oscillation amplitude was approximately 30–35 nm, with an amplitude setpoint of 20–25 nm (corresponding to 70–80% of the free amplitude)."*

Reviewers 3 & 4: Additionally, since cantilevers actual spring constants can deviate strongly from nominal values, it might be a good idea to actually calibrate the cantilever (e.g. Thermal Sweep or Sader's method) and provide results in method section.

The nominal stiffness was 5 N/m, the stiffness of the cantilever used for our measurements was 4.4 ± 0.1 N/m as estimated by thermal sweep method.

Changes made:

- Added to line 486: "... *and stiffness of 4.4 ± 0.1 N/m as estimated by the thermal sweep method. The cantilever's free oscillation amplitude was approximately 30–35 nm, with an amplitude setpoint of 20–25 nm (corresponding to 70–80% of the free amplitude)."*

Reviewers 3 & 4: The authors use the term "high-resolution" AFM to describe their measurements, but what is "high-resolution" AFM exactly? While I do agree that the resolution of the AFM images is adequate for the authors' samples, it is not particularly high resolution. Generally, AFM can achieve atomic resolution (in specific conditions) so 2 nm/pixel is not that high. I would suggest to remove the "high-resolution" modifier or justify its use appropriately.

We agree with the reviewer that the term "high-resolution" can be vague, we now specify our level of noise in the text. We perform our measurements with a noise level on the Z-direction in the order of 20-30 pm (ref. 24 and ref. 25), which is the relevant direction to evaluate the difference in diameter size of DNA alone vs NX11-DNA.

Moreover, the tip convoluted lateral size of DNA is in the order of 10 nm, so Nyquist and Shannon sampling theorems state that accurate reconstruction requires sampling at least twice the spatial frequency of the smallest feature and that 2 nm/px is already more than sufficient for visualizing our molecules. Finally, while atomic resolution is easily achievable on hard materials, it is not the same for soft deformable nucleic acids and protein. Thus, we believe the term "high-resolution" is appropriate for the imaging conditions and sample type used in our study.

Changes made:

- Added sentence (183-186): "*The Z-direction noise level during imaging was 20–30 pm, sufficient to resolve height differences between DNA and NX11-DNA (ref. 24 and ref. 25). Given the tip-convoluted lateral size of DNA (~10 nm), our imaging resolution of 2*

nm/pixel satisfies Nyquist-Shannon sampling criteria, ensuring accurate spatial reconstruction of molecular features."

- Added references:
  - Ref. 24: "Fusco, G. *et al.* Molecular determinants of the interaction of EGCG with ordered and disordered proteins. *Biopolymers* 109, e23117 (2018)."
  - Ref 25: "De, S. *et al.* Soluble aggregates present in cerebrospinal fluid change in size and mechanism of toxicity during Alzheimer's disease progression. *Acta Neuropathol Commun* 7, 120 (2019)."

> **Reviewers 3 & 4:** Ref. 32 is the same as Ref 27

We thank the reviewer for pointing out this duplication. We have removed reference 32.

> **Reviewers 3 & 4:** Fig. S5 has very low resolution and/or small text. It is impossible to read the legends in (d) and, moreover, there seem to be three lines/curves according to the legends, but it is difficult to see that clearly and identify what the curves represent.

Thanks. We have updated Figure S5 with increased resolution and larger text.

> **Reviewers 3 & 4:** Lines 348-352 have a different font and line spacing compared to the rest of the text.

We have double-checked all fonts and life spacing and corrected them where applicable.

Comments from Reviewer 5:

Reviewer 5: The sequence of the DNA is not discussed in the results or discussion. Does this matter? Are there some sequences that work better than others?

Although canonical B-form DNA is often modeled as a uniform helix, numerous crystallographic and molecular dynamics studies have shown that base sequence significantly modulates helical parameters, groove geometry, and flexibility. For example, pyrimidine-rich tracts containing TpT, CpT, TpC, and CpC steps preferentially adopt conformations with reduced twist and altered roll compared to other dinucleotide steps (ref. 43). Likewise, thymine-rich runs such as TTTT have been shown to produce a narrower minor groove than mixed sequences (ref. 44). These types of sequence-dependent groove and helicity variations are now recognized as an important component of DNA shape readout in protein–DNA recognition (ref. 45).

Methylation can further stiffen base steps and modify groove electrostatics, adding another layer of sequence-specific structural modulation. Such deviations—on the order of 1–2 Å in groove width and several degrees in twist per dinucleotide step—are sufficiently large to alter the binding of helically wrapped proteins. Because the NoLimits DNA used here has an undisclosed sequence, we cannot determine whether particular sequence motifs were present, but it is plausible that intrinsic sequence-dependent geometry contributed to the deformations observed in our NX11–DNA complexes.

Changes made:

- Expanded the Discussion (lines 323-328): "*Importantly, although the NoLimits DNA template used here has an undisclosed sequence, DNA sequence-dependent variations in B-form geometry —such as altered twist, roll, and groove width in pyrimidine-rich tracts or methylated regions— are well documented (ref. 43—45) and could plausibly contribute to the deformations we observed in NX11–DNA complexes.*"
- Added ref. 43: "Olson, W. K., Gorin, A. A., Lu, X. J., Hock, L. M. & Zhurkin, V. B. DNA sequence-dependent deformability deduced from protein-DNA crystal complexes. *Proc Natl Acad Sci U S A* 95, 11163–11168 (1998)"
- Added ref. 44: "Gavathiotis, E., Sharman, G. J. & Searle, M. S. Sequence-dependent variation in DNA minor groove width dictates orientational preference of Hoechst 33258 in A-tract recognition: solution NMR structure of the 2:1 complex with d(CTTTTGCAAAAG)(2). *Nucleic Acids Res* 28, 728–735 (2000)"
- Added ref. 45: "Rohs, R. *et al.* The role of DNA shape in protein-DNA recognition. *Nature* 461, 1248–1253 (2009)".

Reviewer 5: Also, to what extent would it be possible to engineer DNA sequence specificity into the TALE fibril?

This is a highly interesting point, and we thank the reviewer for raising this.

DNA sequence specificity can be engineered back into the NX-repeat by inserting the repeat-variable residue (RVD) mutations from natural TALE sequences to make a "nucleo-specific (NS)" repeat (**new Fig. S16**). For example, the NX4 domain can mimic the NTR domain, which natural TALEs require to initiate DNA binding, followed by several nucleo-specific (NS) repeats containing RVD mutations that recognize specific bases. Using biolayer interferometry, we demonstrated that NX4-NS20-NX1 binds its 20 bp target sequence with a K_d ~45 nM, while showing weak interactions (K_d > ~700 nM) to a random DNA sequence. (**new Fig. S17 and new Table S3**).

Changes made:

- Revised Discussion sentence (lines 347-349) to: "*Lastly, we propose that variants of NX11 could be designed to ensure that nucleation occurs at specific DNA sequences by reinstating the RVD mutations, followed by elongation through the addition of sequence-independent binders or subunits (**Fig. S16** and **Fig. S17**).*"
- Added **new Fig. S13** and **new Fig. S17** and **new Table S3**
- Added methods section for BLI to the SI
- Added amino acid sequences of designs NX4-NS14-NX1 and NX4-NS20-NX1 to **Table S1**
- Added new co-author Matthias Mulder.

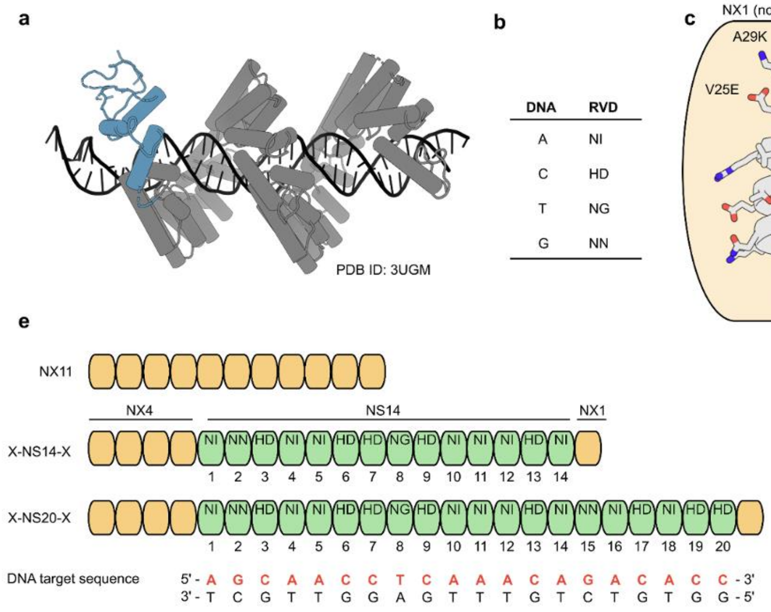

Figure S16. Design concept. **(a)** CrystalTALE PthXo1 structure bound to its target (PDB ID: 3UGM). Blue highlights the N-terminal region (NTR), and gray are the central repeat domains (CRD). **(b)** TALE residue di-variable (RVD) to DNA nucleotide specificity. **(c)** Previously designed DNA non-specific NX-repeat with mutations (S10R and N9K) that remove specific binding, and increase non-specific binding via electrostatics with DNA phosphate backbone. (**d)** NS-repeat, with its native residue divariable (RVD) site in green. Mutations A29K, V25E and C27T mutations from NX-repeat are conserved. But the RVD was re-inserted to bind to specific nucleotides A,C, T, G via different combinations of amino acids at this position. **(e)** Overview of designs generated in this study. In native TALEs the NTR is crucial to initiate DNA binding. We replaced the role of NTR by replacing it with NX4 to initiate non-specific binding to DNA. At the C-terminus a terminal NX1 was included so that potential oligomerization occurs through NX-NX interfaces previously confirmed by cryo-EM. RVD sites were chosen according to the table in panel **(b)**, two designs were generated: X-NS14-X and an

extended X-NS20-X. The DNA target sequence is specific below, with red indicating the target nucleotides.

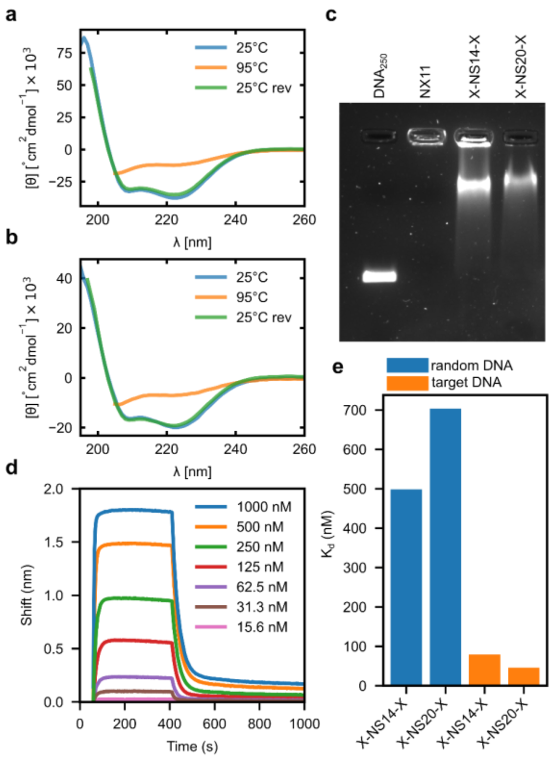

Figure S17. Stability and binding specificity of NX4-NS14/20-NX1 proteins. **(a)** Circular dichroism of **(b)** NX4-NS14-NX1 and **(c)** NX4-NS20-NX1. Molar residue ellipticity [θ] is plotted over wavelength, showing a predominantly α-helical profile. Heating to 95 °C leads to a minimal change in ellipticity, and subsequent cooling to back to 20 °C (20°C rev) gives the same profile as the original at 20 °C, demonstrating that both proteins are thermostable > 95 °C. **(d)** Example biolayer interferometry (BLI) traces of biotinylated target DNA functionalized at the probe, and various concentrations of NX4-NS14-NX1 in solution. Association phase starts at ~50 sec, and dissociation at ~400 sec. Measurements are performed in TBS + 10 mM $MgCl_2$ + 0.5% BSA + 0.5% Tween-20. **(e)** Dissociation constant (K_d) from global fits of NX4-NS14-NX1 and NX4-NS20-NX1 for BLI experiments with target DNA and random DNA.

Table S3. DNA sequences used in bio-layer interferometry experiments. Bold indicates expected binding of proteins. With the NS-repeat targeting sequence highlighted in red. Biotin was functionalized on one of the oligo's at the 5' including a 6 basepair spacer (AAAAAA) and a two bp spacer (GA) at the 3'.

**Design**	**DNA**	**DNA duplex sequence**
X-NS14-X	Target	5'-biotin-AAAAAA**ATCTAGCAACCTCAAACAG**GA-3'<br>3'- TTTTTTTAGATCGTTGGAGTTTGTCCT-5'
	Random	5'-biotin-AAAAAAATCAGACCGACATCTAATCGA-3'<br>3'- TTTTTTTAGTCTGGCTGTAGATTAGCT-5'
	Target	5'-biotin-AAAAAA**ATCTAGCAACCTCAAACAGACACCA**GA-3'<br>3'- TTTTTTTAGATCGTTGGAGTTTGTCTGTGGTCT-5'

X-NS20-X	Random	5’-biotin-AAAAAAATCAGACCGACATCTAATCCGCAACGA-3’<br>3’- TTTTTTTAGTCTGGCTGTAGATTAGGCGTTGCT-5’

Reviewer 5: I am assuming that "peptide alone" would have induced no or very poor antibody response in Figure 4D? Please elaborate on this.

Thank you for raising this point. Yes indeed the peptide alone or inserted into NX11 is expected to be weakly immunogenic (ref. 36).

Changes made:

- Included sentence to Results section (lines 307-311): “*Because NX11, DNA, or the naked NX11–DNA complex are not expected to elicit PfCSP-specific immune responses in the absence of an antigen, and unassembled peptide would likely be weakly immunogenic (ref. 36), we concentrated on comparing the antigen-displaying constructs and included benchmark and naïve groups to confirm the specificity of the response.*”
- Added ref. 36: Bachmann, M. F. & Jennings, G. T. Vaccine delivery: a matter of size, geometry, kinetics and molecular patterns. *Nat Rev Immunol* **10**, 787–796 (2010).

Comments from Reviewers 1, 3, 4 and 5:

> **Reviewer 1:** The authors have adequately addressed the issues I raised. I support the publication of the revised paper.

We thank Reviewer 1 for their thorough evaluation and support.

> **Reviewer 3:** I co-reviewed this manuscript with one of the reviewers who provided the listed reports. This is part of the Nature Communications initiative to facilitate training in peer review and to provide appropriate recognition for Early Career Researchers who co-review manuscripts.

We thank Reviewer 3 for their contribution to the review process.

> **Reviewer 4:** The manuscript "Computational redesign of TALE proteins for DNA-templated assembly of protein fibers" by Robbert J. de Haas et al. was thoroughly revised. I am fully convinced that the AFM images are devoid of artifacts that could influence the analysis and that the measured periodic fluctuations are indeed caused by NX11 proteins. I found no further issues and fully support the publication of the manuscript.

We thank Reviewer 4 for their careful assessment and support.

> **Reviewer 5:** The authors have responded productively to reviewer concerns. I have no further suggestions.

We thank Reviewer 5 for their constructive feedback throughout the review process.

Comments from Reviewer 2:

> **Reviewer 2, Point 1:** Discussion on the assembly mechanisms and defects remains unclear. Revised discussion on introducing local defects is taken out of context and does, in no instance, refer to problems that a possible mismatch in the helical twist of NX11 and the DNA cause. Instead, speculations about the mismatch are raised when talking about the different periodicities observed in AFM. As the response of 7 ± 2 nm corresponding most likely to a single NX11 molecule given to Reviewer 1, this would be important to mention in the text.

We thank the reviewer for this constructive feedback and agree that the discussion of assembly defects and the AFM periodicities could be better connected. We have reorganized the relevant text to more clearly link the helical twist mismatch to the observed defects and structural heterogeneity. Additionally, we have now included the interpretation that the minor periodicity of ~7 ± 2 nm likely corresponds to a single NX11 molecule bound to DNA, as this provides important context for understanding the assembly at the molecular level.

Changes made:

- <u>Revised the Results section</u> (lines 231-236) to include: "*The minor periodicity (~7 ± 2 nm) likely corresponds to a single NX11 molecule, consistent with the helical pitch of one NX11 unit observed in cryo-EM. The major periodicity (~40 ± 10 nm) corresponds*

to approximately 5 NX11 molecules, suggesting a larger-scale repeating structural unit that may arise from a cumulative mismatch between the NX11 superhelical twist and the helical twist of the DNA template."

- Revised the Discussion section (lines 359-363) to explicitly connect the helical twist mismatch to both the observed AFM periodicities and the structural heterogeneity that complicates high-resolution cryo-EM reconstruction: "*A slight mismatch between the NX11 superhelical twist and the DNA helical twist would progressively build up frustration along the assembly, introducing periodic defects at regular intervals. This is consistent with the ~40 nm major periodicity observed by AFM, and likely also contributes to the structural heterogeneity that has prevented high-resolution 3D reconstruction by cryo-EM.*"

> **Reviewer 2, Point 2:** The assembly process itself is suggested to be cooperative and driven by nucleation followed by elongation. How does the added experiment on assembling a fusion protein of NX11-sfGFP give information on the assembly process, especially if conducted at excesses that are too low for a full coating (at least judging from the EMSA gel given in Fig. S24 for NX11)? Also, wouldn't the fusion protein possibly interfere with the assembly process?

We thank the reviewer for this comment. The sfGFP-NX11 experiment at 0.5X molar excess was specifically designed to probe assembly behavior under sub-stoichiometric conditions, where not enough protein is available to fully coat the DNA. The intent was to determine whether the limited NX11 available would distribute evenly along the DNA (consistent with independent, non-cooperative binding) or form localized clusters (consistent with cooperative NX11-NX11 interactions contributing to assembly). The observation of discrete protein clusters rather than uniform coating supports the interpretation that NX11-NX11 interactions promote local accumulation, even though these interactions are not strong enough to drive fully cooperative assembly under these conditions.

Regarding the sfGFP fusion: we acknowledge that the N-terminal GFP fusion could alter assembly behavior, for instance by introducing steric constraints at the protein-protein interface. However, the terminus of sfGFP is flexible, as is the N terminus of NX11, which we expect would enable accommodation of the fused GFP.

The sfGFP tag served as an electron-dense label to unambiguously identify protein-bound regions in negative stain EM, which would otherwise be difficult to distinguish from DNA alone. We have revised the text to more clearly state the purpose and limitations of this experiment.

Change made:

- Revised the text accompanying the sfGFP-NX11 experiment (lines 188-190) to clarify: "*While the N-terminal sfGFP fusion may introduce steric effects that alter assembly behavior, it served as an electron-dense label to unambiguously identify protein-bound regions by negative stain EM.*"

> **Reviewer 2, Point 3:** The authors mention that "NX11-NX11 interactions may slow down the diffusion and this is a nucleation event for elongation of NX11 on the DNA fiber". Does this mean that there first exists protein-protein interaction in solution, followed by nucleation on the DNA substrate? If there are already NX11-NX11 interactions, does this mean that more than

one protein could be expected to move along the DNA filament as a unit before nucleation happens? In that case, what's the critical number of proteins for nucleation?

We thank the reviewer for pressing on this mechanistic point and apologize for the ambiguity in our previous wording. NX11 does not form protein-protein interactions in solution, as demonstrated by SEC (Fig. 2a): NX11 elutes as a monomer even at high concentration, confirming the absence of oligomerization prior to DNA binding (Results Section lines 183-186).

The nucleation event we refer to occurs *exclusively* on the DNA template: individual NX11 monomers first bind to DNA through non-specific electrostatic interactions, and when two or more DNA-bound NX11 molecules slide along the DNA, they can form NX11-NX11 interfaces. The NX11-NX11 interfaces then locally stabilize the assembly and promote elongation by favoring the binding of additional NX11 monomers at the growing ends of the cluster.

Changes made:

- Revised the Results section (lines 140-141) to clarify: "*This suggests a two-phase assembly, with a rapid nucleation driven primarily by electrostatic interactions between multiple NX11 molecules and DNA, followed by a slow re-arrangement of NX11 on DNA*".

Reviewer 2, Point 4: How representative are the DLS results for nucleation, if DNA48 was used for the experiments, which can have up to 4 NX11 units assembled?

The reviewer raises a valid point. The DLS data for NX11-DNA48 (Fig. 2d) were not intended to characterize the nucleation process, but rather to demonstrate that NX11-DNA complexes assemble into well-defined particles without higher-order aggregation. The nucleation and assembly kinetics were instead studied using static light scattering with DNA250 (Fig. 2c), which provides a much longer template (~250 binding sites) and is therefore more representative of the multi-step nucleation-elongation process.

For the short DNA_{48} template (~4 NX11 binding sites), assembly is expected to be rapid and essentially complete, with limited opportunity to observe intermediate nucleation states. We agree that this should be more clearly communicated in the text.

Changes made:

- Moved DLS results to separate paragraph and revised wording (lines 143-146): "*Analysis of smaller NX11-DNA_{48} assemblies showed these had a hydrodynamic diameter of 18.3 nm and a polydispersity index of 0.12 as measured by dynamic light scattering (DLS) (**Fig. 2d and Fig. S2**), demonstrating that NX11-DNA complexes are monodisperse and free of higher-order aggregates.*"

Reviewer 2, Point 5: Regarding the immunogenicity studies, the authors assume that NX11 alone, DNA alone and NX11-DNA complexes do not elicit an immune response, and added I53.dn5_majorX5 as positive control. These controls are still not sufficient to back up the claim that organizing the antigens makes a difference. Each of the NX-epitope variants must be tested for their respective response alone, especially since the complexes have not been

purified from excess proteins (the authors state that these could contribute to immunogenicity: the reason why the excess was reduced to 1.1x in the antigen display studies).

We believe the current set of controls — a naïve (untreated) group confirming baseline, and I53_dn5_majorX5 as a benchmark multivalent immunogen display — are appropriate for a proof-of-concept demonstration. The naïve control confirms that the observed antibody titers are antigen-specific, while the I53_dn5_majorX5 benchmark provides context for the magnitude of the response.

With respect to testing each NX-epitope variant alone (i.e., without DNA assembly): the 1.1X excess used represents a minimal amount of unbound protein. Furthermore, based on the established literature, short peptide epitopes presented on monomeric protein scaffolds are expected to be weakly immunogenic compared to multivalent display (ref. 36). We therefore consider it unlikely that the small amount of unbound NX-epitope monomer contributes meaningfully to the observed immune responses.

We have revised the text to more clearly frame the immunogenicity data as a proof-of-concept and to acknowledge the limitations of the study design, including the suggestion for more comprehensive controls in future work.

Changes made:

- Added to the Discussion section (lines 389-391): "*Including additional controls in these studies, such as unassembled NX-epitope monomers and naked NX11-DNA complexes, will be valuable for quantifying the specific contribution of DNA-templated multivalent display to immunogenicity.*"

Reviewer 2, Point 6: If in vivo stability is too difficult to study, the authors should at least investigate the stability of the complexes in simulated conditions (presence of serum, nucleases, competing DNA binding agents etc.).

We appreciate this suggestion. We note that our manuscript already includes a nuclease protection assay (Fig. 2f) demonstrating that NX11-DNA complexes resist Benzonase degradation for >18 hours under optimal nuclease activity conditions (37°C, with Mg^{2+} and Ca^{2+} cofactors). Benzonase is a highly aggressive endonuclease that degrades all forms of DNA and RNA, and this challenge is substantially more stringent than what would be encountered from serum nucleases in vivo. Furthermore, the Benzonase challenge was performed in a buffer containing divalent cations that could in principle compete for DNA binding, yet the NX11 coating remained protective.

While a comprehensive stability study under simulated physiological conditions (e.g., serum, competing polyanions) is beyond the scope of the current work, we agree that this is an important direction for future translational development and have expanded our discussion accordingly.

Changes made:

- Revised the Discussion section (lines 387-388) to: "*Direct assessment of NX–DNA complex stability in vivo remains technically challenging, and we anticipate that future*

studies including exposure to serum, competing DNA-binding agents, and physiological ionic strength will be needed to address this important aspect of translational development."
